# Investigating effective testing strategies for the control of Johne's disease in western Canadian cow-calf herds using an agent-based simulation model

**DOI:** 10.3389/fvets.2022.1003143

**Published:** 2022-11-25

**Authors:** Paisley Johnson, Lianne McLeod, Yang Qin, Nathaniel Osgood, Leigh Rosengren, John Campbell, Kathy Larson, Cheryl Waldner

**Affiliations:** ^1^Large Animal Clinical Sciences, Western College of Veterinary Medicine, Saskatoon, SK, Canada; ^2^Department of Computer Science, University of Saskatchewan, Saskatoon, SK, Canada; ^3^Rosengren Epidemiology Consulting, Midale, SK, Canada; ^4^Agricultural and Resource Economics, College of Agriculture and Bioresources, Saskatoon, SK, Canada

**Keywords:** Johne's disease, beef cattle, agent-based model, testing, disease control

## Abstract

Johne's disease is an insidious infectious disease of ruminants caused by *Mycobacterium avium* subspecies *paratuberculosis* (MAP). Johne's disease can have important implications for animal welfare and risks causing economic losses in affected herds due to reduced productivity, premature culling and replacement, and veterinary costs. Despite the limited accuracy of diagnostic tools, testing and culling is the primary option for controlling Johne's disease in beef herds. However, evidence to inform specific test and cull strategies is lacking. In this study, a stochastic, continuous-time agent-based model was developed to investigate Johne's disease and potential control options in a typical western Canadian cow-calf herd. The objective of this study was to compare different testing and culling scenarios that included varying the testing method and frequency as well as the number and risk profile of animals targeted for testing using the model. The relative effectiveness of each testing scenario was determined by the simulated prevalence of cattle shedding MAP after a 10-year testing period. A second objective was to compare the direct testing costs of each scenario to identify least-cost options that are the most effective at reducing within-herd disease prevalence. Whole herd testing with individual PCR at frequencies of 6 or 12 months were the most effective options for reducing disease prevalence. Scenarios that were also effective at reducing prevalence but with the lowest total testing costs included testing the whole herd with individual PCR every 24 months and testing the whole herd with pooled PCR every 12 months. The most effective method with the lowest annual testing cost per unit of prevalence reduction was individual PCR on the whole herd every 24 months. Individual PCR testing only cows that had not already been tested 4 times also ranked well when considering both final estimated prevalence at 10 years and cost per unit of gain. A more in-depth economic analysis is needed to compare the cost of testing to the cost of disease, taking into account costs of culling, replacements and impacts on calf crops, and to determine if testing is an economically attractive option for commercial cow-calf operations.

## Introduction

Johne's disease is a form of contagious and chronic, incurable enteritis in ruminants caused by persistent infection with the bacteria *Mycobacterium avium* subspecies paratuberculosis (MAP). The clinical stage is characterized by severe diarrhea resulting in dehydration, emaciation, and eventual death (1). Prior to the clinical stage of disease, affected animals can remain in a subclinical state for up to several years, during which time they shed the bacteria in their feces, leading to environmental contamination and the subsequent infection of other animals (2). Available diagnostic tests, such as ELISA and PCR, cannot detect latent infections and have a limited ability to detect MAP in subclinically infected animals, making it difficult to identify and remove sources of infection from within the herd (3).

In addition to challenges related to the diagnosis of infected animals, there are economic losses associated with MAP infection in beef herds including premature culling of animals, reduced slaughter value for clinical animals in poor body condition, lower calf weaning weights from infected dams, veterinary and testing costs, and the loss of reputation and genetics for seedstock operations (4–6). The absence of a successful treatment or an effective vaccine for MAP (7–9), negative economic impacts, animal welfare concerns and the potential for the prevalence of MAP to increase in western Canadian cow-calf herds in the next several years provide incentive for effective control strategies in infected herds.

Previous research on the control and management of Johne's disease has focused primarily on dairy cattle due to the higher prevalence of disease within the dairy industry (10, 11). Much of the currently available published research suggests hygienic calf rearing practices, removing the calf from the dam within 24 hours of birth, keeping a closed herd and adoption of biosecurity measures are effective methods for controlling Johne's disease in dairy herds (10, 12). However, due to vast differences in the management of beef and dairy cattle, the implementation of such control methods is not feasible on most Canadian beef operations (5, 13). Testing and culling have been identified as the primary control options in beef herds, although there is limited evidence supporting the benefits of testing and culling or specific testing strategies, such as the recommended method and frequency of testing (4, 5, 7, 14, 15). A comparison of testing strategies is needed to inform veterinarians and producers regarding best practices for controlling the disease on extensively managed cow-calf operations.

Studying Johne's disease using clinical trials or observational methods is challenging due to the prolonged preclinical stages of disease during which infectious animals appear clinically healthy. Research on the effective control of Johne's disease requires long-term follow up. Simulation tools provide a practical and cost-effective option for evaluating Johne's disease control strategies and the associated economic impact that are not limited by the practical constraints of field trials. Agent-based modeling (ABM) provides a framework to study individual animals that interact in a way that mimics typical herd management practices.

Infection can be represented for individual animals by rules governing transmission and the progression of the stages of infection. Options for disease control can be imposed on the dynamic model of the production system, including testing and culling with various herd replacement strategies, as well as management practices that influence transmission risks. ABMs allow the modeling specific connections between cows and their calves and the evaluation of management decisions guided by individual animal risk factors. Using ABMs, animals can be individually monitored from infection to the time when they start to shed MAP, and then again until they develop typical clinical signs of diarrhea and rapid weight loss that ultimately result in either death or culling. The ABM can then generate the resulting simulated prevalence of MAP based on counts of individual animals at specific stages of infection (16).

A recent review paper of age-structured simulation models to support management decisions for cattle (16) identified 21 models of MAP infection with only two describing the disease in beef herds. Both beef studies were compartmental models; one was stochastic (17) and the other deterministic (7). Six stochastic ABMs were identified for dairy cattle (18–23). An additional ABM was noted in the discussion (24) and examples of other recently published reports using ABMs (25, 26) also examined control of MAP in dairy herds. In addition to highlighting the limited application of ABMs in beef herds, the authors identified opportunities to enhance structure and documentation in reporting (16).

The objective of this study was to compare the simulated prevalence of animals shedding MAP following no testing to the prevalence with different scenarios for testing and culling in extensively managed cow-calf herds. Scenarios investigated varying the type of test (serum ELISA, individual, and pooled fecal PCR) used by beef veterinarians through regional laboratories, as well as the frequency of testing, and the number and risk profile of animals targeted for testing. An agent-based model that represented the composition and management typical of western Canadian cow-calf herds was used to estimate the effect of each testing scenario on the simulated prevalence of MAP for 10 years after the start of testing. The second objective was to compare the costs of each testing scenario to identify the options with the lowest direct testing costs that were also the most effective at reducing within-herd disease prevalence.

## Materials and methods

### Model description

A complete model description available as a Supplementary file follows the Overview, Design concepts, and Details (ODD) protocol for describing individual- and ABMs (27, 28). Highlights are briefly described here. A working and customizable version of the model with selected graphical outputs and downloadable results for scenario testing and independent validation is available at: https://www.beefresearch.ca/tools/johnes-disease-calculator/. Model parameters and herd attributes can be modified on the user interface to customize the model output for specific herd and scenario comparisons.

### Purpose

A stochastic, continuous-time ABM was developed using AnyLogic software (AnyLogic version 8.7) to provide a template for studying Johne's disease progression and testing for control in a commercial cow-calf herd in western Canada.

### Herd structure and management

The specific model version and configuration used for this analysis represented a simplified production cycle starting April 1st within a beef herd consisting of 300 cows older than 2 years of age, 50 yearling heifers and a target ratio of at least 1 bull to 20 cows (Table 1). The cow-calf production cycle was simulated over a time period of 10 years, with a month being the default unit of time measurement. The model was structured to maintain a target number of calving cows, where the numbers of females fluctuated throughout the production cycle as would be expected in a typical herd undergoing culling and replacement. Females exited the herd through culling due to failure to become pregnant, old age at 12 years (42) or Johne's disease (clinical disease or positive test results), and calves were sold post-weaning, with the exception of retained replacement heifers. Of the total number of heifer calves born into the herd, up to 50% were considered eligible to be retained as replacements. Females were brought into the herd as purchased pregnant cows only if the number of retained heifers from the previous year was not adequate to maintain the target number of pregnant cows at calving. Bulls were purchased as needed every year as yearlings, 1 month after calving and 2 months before bull exposure to maintain the target cow to bull ratio. Bulls exited the herd through culling due to old age at 6 years (29) or Johne's disease (clinical disease or positive test results). Calves were born into the model on April 1st at model initialization and each subsequent year.

**Table 1:** Model input parameters and associated references.

**Parameter**		**References**	**Other ABM simulation studies using similar values**
**Initial herd characteristics**			
Number of cows older than 2 years	300	Expert opinion	N/A
Number of yearling heifers	50	Expert opinion	N/A
Cow to bull ratio	20:1	Waldner et al. (29) Expert opinion for high risk estimate of bull purchase numbers	N/A
Percent of heifers eligible for replacements	50%	Expert opinion	N/A
Percent of cows pregnant in body condition 3.0–4.0/5.0	93.2%	Waldner et al. (30) Waldner et al. (31)	N/A
Pregnancy rates for cows with BCS 2.5/5.0 and 2.0/5.0	90%, 78%	Calculated in the model from odds ratios reported in Waldner et al. (32)	N/A
Percent of heifers pregnant in body condition 3.0–4.0/5.0	90.3%	Waldner et al. (30) Waldner et al. (31)	N/A
Pregnancy rates for heifers with BCS 2.5/5.0 and 2.0/5.0	86%, 71%	Calculated in the model from odds ratios reported in Waldner et al. (32)	N/A
**Calf preweaning mortality and weaning weight**			
Calf mortality rate for cows	5.4%	Waldner et al. (30) Waldner et al. (31)	N/A
Calf mortality rate for heifers	7.8%	Waldner et al. (30) Waldner et al. (31)	N/A
Weaning weight difference based on cow age	Input table	BCRC (33)	N/A
Weaning weight difference between males and females	23 kg (50 lbs)	Expert opinion	N/A
Weaning weight difference for moderate MAP shedder	41 kg (90 lbs)	Bhattarai et al. (6)	N/A
Weaning weight difference for heavy MAP shedder	59 kg (129 lbs)	Bhattarai et al. (6)	N/A
**Initial herd infection status and transmission risk**			
Initial percent of herd infected (not infectious)	5.0%	Johnson et al. (34) and expert opinion	Al-Mamun et al. (25) also modeled equal starting prevalence of latent and low shedders
Initial percent of herd subclinically infected	5.0%	Johnson et al. (34) Minimum within herd prevalence based on pooled fecal PCR results (20 per herd)	
Initial percent of herd clinically infected (to initiate testing)	1.0%	Expert opinion to reflect number of cases likely to prompt diagnostics	N/A
Percent of purchased bulls infected	1.0%	Johnson et al. (34) True prevalence for cows from western Canada estimated with Bayesian latent class analysis	N/A
Percent of purchased bulls subclinical	1.0%	Johnson et al. (34)	N/A
Percent of purchased cows infected	1.0%	Johnson et al. (34)	N/A
Percent of purchased cows subclinical	1.0%	Johnson et al. (34)	N/A
Probability of in utero transmission from subclinical cow	0.09^†^ (0.06,0.14)	Whittington and Windsor (35)	Kirkeby et al. (19)
Probability of in utero transmission from clinical cow	0.39^†^ (0.20,0.60)	Whittington and Windsor (35)	Kirkeby et al. (19)
Coefficient representing shedding in subclinical cows as compared to clinical	0.2	Expert opinion	Other coefficients based on expert opinion (19) Or estimated in dairy model (23)
Probability of dam to calf transmission from birth to weaning from clinical cow	0.557^†^^†^	Calibrated result But similar to report by Windsor and Whittington (36)	N/A
Probability of dam to calf transmission from birth to weaning from subclinical cow	0.2 × 0.557^†^^†^	Calibrated result But similar to report by Windsor and Whittington (36)	N/A
Frequency of infective contact = contact rate × probability of infection given infectious contact for preweaning calves (calves 0 to 7 mo)	46.89/year^†^^†^	Calibrated result	N/A
Weighted prevalence of simulated MAP shedding intensity = (# subclinical cows × 0.2 + # clinical cows) / total cows > 2 years of age	–	Calculated in real time	Robins et al. (22) & Al-Mamun et al. (18): frequency-dependent transmission Robins et al. (22) environmental dependent on infectious cows
Infection rate / year = frequency of infective contact × weighted prevalence	–	Calculated in real time	
Post weaning risk of infection modifier applied to frequency of infective contact (calves 7 mo to 1 yr)	0.50^†^ (0.30,0.70)	Windsor and Whittington (36) & Expert opinion	Biemans et al. (37) and Kirkeby et al. (19) Risk was function of age based on expert opinion
Adult risk of infection modifier applied to frequency of infective contact (cattle > 1 yr)	0.19^†^ (0.10,0.32) × (1/current age in years)	Windsor and Whittington (36) & Expert opinion	Biemans et al. (37) and Kirkeby et al. (19) Risk was function of age based on expert opinion
**Disease progression timeline:**			
Infected Duration (months) (latent/silent infection) (Not shedding)	30 (18, 54)	Weber et al. (38) Elliott et al. (39)	Biemans et al. (37) Camanes et al. (24) Al-Mamun et al. (18) Robins et al. (22)
Subclinical Duration (months) (Low to moderate shedding) (With duration of latent infection leads to most animals developing clinical signs at 5 years with a range of 2.5 to 10 years.)	24 (6, 60)	Tiwari et al. (1)	Biemans et al. (37) Camanes et al. (24) Al-Mamun et al. (18) Robins et al. (22)
Time from first clinical signs to removal from herd (months) (High shedding)	2 (1, 4)	Expert opinion Tiwari et al. (1)	Biemans et al. (37)
Maximum age (years) of clinical disease	10	Tiwari et al. (1)	N/A
**Diagnostic test performance**			
Minimum age (months) of testing	24	Expert opinion	Biemans et al. (37) Camanes et al. (24)
Sensitivity of serum ELISA, fecal PCR and pooled fecal PCR—infected (latent not infectious)	0	Whitlock et al. (40)	Biemans et al. (37) Camanes et al. (24) Verteramo Chiu et al. (23) Robins et al. (22)
Sensitivity of serum ELISA—subclinical (moderate shedder)	0.36 (0.22,0.52)	Johnson et al. (34)	Biemans et al. (37) Camanes et al. (24) Verteramo Chiu et al. (23)
Sensitivity of serum ELISA—clinical (heavy shedder)	0.825 (0.80, 0.87)	Bech-Nielsen et al. (41) Sweeney et al. (3)	Bennett et al. (7) Other estimates slightly higher (22) or lower (37)
Specificity of serum ELISA	0.99 (0.98,0.99)	Johnson et al. (34)	Biemans et al. (37)
Sensitivity of fecal PCR—subclinical and clinical (moderate and heavy shedders)	0.96 (0.80,1.00)	Johnson et al. (34)	See sensitivity analysis
Specificity of fecal PCR	0.98 (0.96,1.00)	Johnson et al. (34)	N/A
Sensitivity of pooled fecal PCR—subclinical and clinical (moderate and heavy shedders)	0.54 (0.36,0.72)	Johnson et al. (34)	N/A
Specificity of pooled fecal PCR	0.99 (0.99,1.00)	Johnson et al. (34)	N/A

† To simplify parameter calibration scenarios only the mean value was used. The impact of the distributions was then examined using the distributions in the Monte Carlo simulations and reported with the sensitivity analysis.

†† Values used in comparison of testing and culling strategies.

The environment within the model consisted of a series of three holding areas: a summer pasture used for breeding, a winter holding area where the cows calve, and a pen where the bulls are held outside the breeding season. The cow herd moved to and from the winter holding area and the summer pasture as one unit and the bulls moved from the bull pen to the summer pasture for the duration of the breeding season and then back to the bull pen as one unit.

### Agents and state charts

#### Cow agent

The primary agents in the model were individual cows, bulls and calves organized into a single herd. There was also an agent representing the testing process and a “main” agent containing the environment in which the cattle were located. The cattle agents were governed by a series of five interacting state charts that describe, characterize and evolve the state of each animal. The five state charts defined: 1) the stage of production; 2) the cow-calf production cycle; 3) movements between winter pens and summer pasture; 4) stages of Johne's disease infection; and 5) blood and fecal testing (pooled and individual) and associated culling.

#### Stage of production state chart

The production stage state chart (Figure S1 in model description supplement) describes the current classification of an animal and its evolution. Stages included preweaning or nursing calves, weaned heifers and steers, postweaning replacement heifers, bulls, breeding females, and purchased cows or heifers. To describe the cow agent in further detail, the “breeding females” state was further subdivided into replacement heifers before breeding, heifers exposed to bulls, pregnancy tested heifers, and cows. The calculation of how many heifer calves to retain was made after pregnancy testing the bred heifers and cows, and culling cows for old age. After all culling was completed and the number of retained pregnant females was determined, pregnant cows were purchased if necessary to maintain the number of calving females.

#### Production cycle state chart

**Figure 1:**
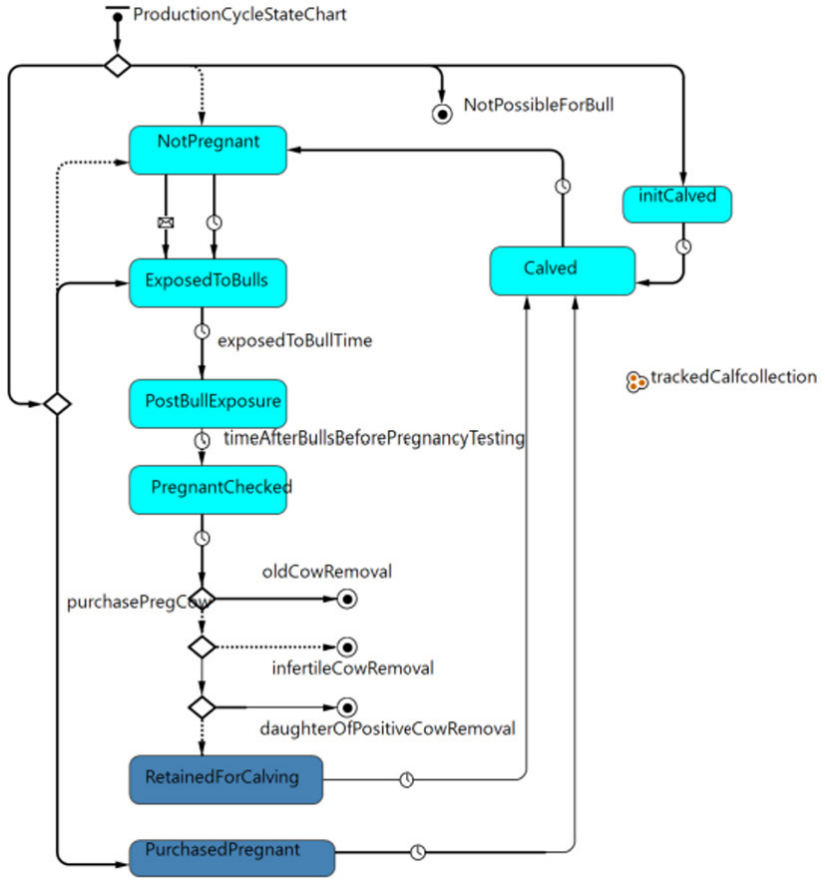
Production cycle state chart governing the movement of heifers and cows through the stages of breeding, pregnancy and calving within the model.

The cow-calf production cycle state chart (Figure 1) governed how heifers and cows moved through each stage of the breeding, pregnancy, and calving cycle. For simplicity, all cows and pregnant heifers calved on April 1st of each year. Cows were exposed to bulls 3 months after calving for 3 months. One month after the bulls were removed, females were pregnancy tested, and 5 months after a positive pregnancy test, a female calved after a total gestation of 9 months. The probability that a cow was pregnant at pregnancy testing varied based on whether the animal was a heifer or cow and also varied based on body condition score (BCS) as informed by previously published field data (32) and her MAP status. After pregnancy testing, cows were removed from the herd for old age (> 12 years) (42), if not pregnant, or if they were in the clinical stage of infection for Johne's disease.

Calves were introduced into the model at the calving event. Information about the dam was passed to the calf, including the dam's age, whether the dam was purchased and the dam's MAP status. Other information recorded at birth included calf sex and birthweight. The calf's rate of gain and probability of survival to weaning varied based on the dam's age and the calf's sex; the dam's MAP status and stage of infection also influenced the rate of gain (Table 1). The cow's information was updated with a record of the identity of the calf and the total number of calves to date for the cow. The cow and calf were connected allowing for the identification and culling of daughters from positive cows for risk-based testing programs or for culling daughters of clinically infected dams without testing.

#### Cow movement state chart

A movement state chart (Figure S8 in model description supplement) informed animal movements between the summer pasture, female winter pen and bull winter pen based on events corresponding to herd management. Cows and bulls were moved to the summer pasture together 3 months after calving. The bulls remained with the cows for 3 months and then they were returned to their home pen. The cows remained on summer pasture until simultaneous pregnancy testing and weaning.

#### Johne's disease state chart

**Figure 2:**
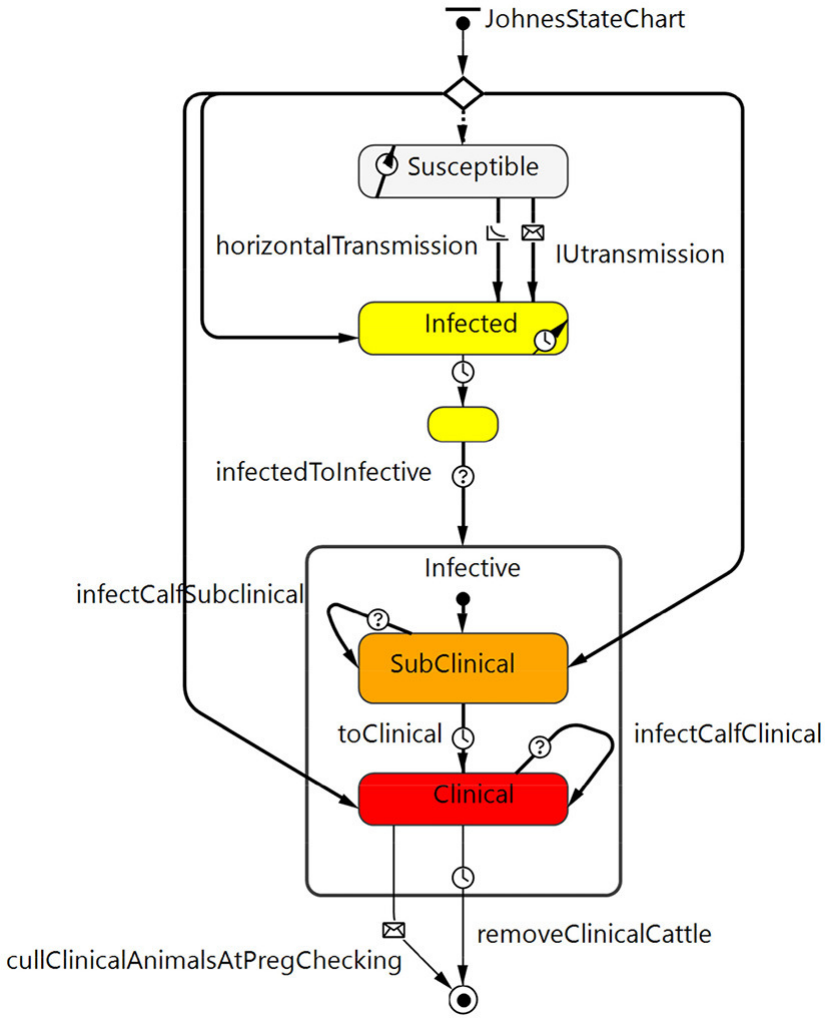
Johne's disease state chart that describes the stages of MAP infection (latent infected but not infectious through to subclinical and then clinical) and disease progression as well as transmission routes from cow to calf within the model.

The Johne's disease state chart (Figure 2) described the stages of infection with MAP and progression to clinical disease as well as MAP transmission from cow to calf (*in utero* or *via* fecal transmission from birth to weaning) and other fecal transmission of MAP either directly from animal to animal or through the environment. Disease states included susceptible, latent infection (silent, infected but not infectious), and infective. Infective animals included subclinical MAP infection (moderate shedding) and clinical disease (high shedding, with apparent clinical signs of diarrhea and weight loss). The model assumes for simplicity that Johne's positive cows are culled before they die given an active, voluntary control program.

All animals in the herd were placed in this state chart at the start of the model based on the initial prevalence (Table 1). The initial prevalence was chosen to reflect MAP-infected herds at the time the herd owner would be likely to observe the first clinical cases, recognize the problem and test. The chosen value was the minimum observed within herd prevalence from positive herds in a national survey (34). It was also consistent with the first whole herd test results from the 10 positive herds that provided calibration data for this analysis (Table 2). Existing testing data were applied to the infectious subclinical stage of the model. The initial prevalence value for the latent stage was assumed to be the same.

**Table 2:** Longitudinal prevalence data from Saskatchewan Stock Growers Association (SSGA) Johne's disease surveillance program.

**Year**	**Number of**	**Number of**	**Number of test**	**Apparent**	**True**	**95% CI for**
	**herds tested**	**animals tested**	**positive animals**	prevalence*	prevalence**	**true prevalence**
1	10	1,270	76	6.0%	7.4%	5.6%, 9.6%
2	3	346	12	3.5%	3.7%	1.5%, 7.4%
3	4	463	28	6.1%	7.5%	4.8%, 11.4%
4	6	768	38	4.9%	5.9%	3.8%, 8.5%
5	7	1,156	23	2.0%	1.5%	0.4%, 2.9%
6	7	1,196	24	2.0%	1.5%	0.5%, 2.9%
7	6	920	28	3.0%	3.1%	1.6%, 5.0%

* Apparent prevalence based on serum ELISA test.

** Estimated using Rogan and Gladen (43) method with Clopper-Pearson 95% confidence intervals (44).

Purchased cattle were also assigned initial states based on expected prevalence (Table 1). Initial prevalence estimates were based on a recent observational study reporting BLCM estimates of true prevalence for Canadian cow-calf herds (34). Purchased animals were assumed to be a random animal from the Canadian cow-calf population and not specifically from an infected herd (34).

Infection of susceptible animals occurred by direct cow to calf transmission *in utero* or through fecal contamination from infected dams from birth to weaning and among other animals through direct fecal transmission or indirectly through fecal contamination of the environment (35, 45, 46). Calves born to infective cows had a probability drawn from a pert distribution for each simulation of contracting MAP vertically *in utero* depending on if the dam was in the subclinical or clinical stage of disease (35) (Table 1). Calves could also be infected through fecal contamination from the dam before weaning based on a probability depending on the dam's stage of infection (36, 47). This probability was estimated through calibration procedures described in subsequent sections (Table 1).

The description of all other fecal transmission was an intentional simplification. As the infection was primarily transmitted through feces, a choice was made to not distinguish between animal-to-animal fecal oral transmission and transmission through fecal contamination of the environment due to the extensive management of most herds throughout the year and difficulty in meaningfully modeling risks of transmission in specific environments as reported by others for more intensively managed herds (17, 48). Susceptibility to infection varied based on animal age (Table 1). Calves <6 months of age were most susceptible, calves between 6 months and 1 year were considered to be about half as likely to be infected given exposure and calves older than 1 year were substantially less likely to be infected given exposure (36, 47). The age-related coefficients used to adjust the probability of infection given contact were drawn from pert distribution as described in Table 1, and for adults sequentially decreased by an inverse function based on age in years.

The rate of infection per year was a dynamic value based on the current simulated prevalence of infective animals as well as a calibrated parameter representing the difficult to differentiate combination of probability of infection given contact between a susceptible individual and MAP shed by infectious cattle and a contact rate [frequency of infective contact (Table 1)] (details in the Calibration section). The base value of this calibrated parameter reflected the potential for horizontal transmission from MAP shed by infectious adults to preweaning calves. The rate was then age-adjusted for postweaning calves and for adults (Table 1). The weighted prevalence of subclinical (moderate shedders) and clinical animals (heavy shedders) (Table 1) provided a measure of the current risk of exposure to MAP. Subclinical animals or moderate shedders were assumed to contribute to the weighted prevalence 20% as much as clinical animals to reflect previously reported values (Table 1).

A set of transitions associated with rates were employed to govern progression from infected to clinical disease stages based on limited data from the existing literature (1, 2, 12, 49). The mean duration associated with a timeout was drawn from a pert distribution informed by mode, minimum and maximum values reflecting both limitations in the available literature and the biological variability of the course of infection among different animals (Table 1). The rate of transition to the next state for each event within a run was based on an exponential distribution as 1/mean of the selected duration for each simulation.

All cows with clinical Johne's disease, exemplified by progressive diarrhea and severe weight loss, were removed either within a specified period after clinical signs began or at pregnancy testing, depending on which was sooner. The period from clinical signs to removal was governed by a pert distribution to account for the variability in identifying cows with clinical signs and removal from the herd (Table 1). The values were chosen to represent the range of values likely with a voluntary control program where cows would be culled after testing and before calving.

#### Fecal and blood testing state charts

The fecal and blood testing state charts (Figures S4, S5 in model description supplement) characterized the process of individual sample fecal and blood testing for Johne's disease. Based on the test results, the cow was either retained or removed from the herd. The details of testing timing, frequency and duration were governed by messages sent from state charts in a separate test agent.

The model also allowed for testing of pooled fecal samples to save money on testing costs. Fecal samples from five animals contributed to each pool as offered by the regional laboratory and supported by previous research (50). In the event of a positive test result for the pool, all the samples in the pool were individually retested to identify positive animals for culling. If the pool was negative, all animals in the pool were considered test negative.

#### Weight and BCS agent

The dynamics of weight and BCS were encapsulated as a hierarchical agent inside each cow agent, capturing continuous weight change of calves, heifers, and cows and categorical changes in BCS on a 5-point scale (51). The model employed a daily event to simulate average daily weight gain. Individual predicted weaning weights informed average daily gain and were drawn from baseline distributions derived from local calf sale weight data with individual values dependent on the dam's age, calf sex, and dam's MAP status and updated with any change in the dam's MAP status (Table 1). The target 205 day weaning weight for steer calves from mature, healthy cows was 273 kg (SD 9 kg) [600 lbs (SD 20 lbs)].

Heifer calves retained in the herd continued to gain weight until they reached a distribution of target weights at first bull exposure (65% of mature weight) and then target weight at the time of their first calf (85% of mature weight). The model was simplified such that cows did not gain weight due to growth after their second calf. Mature cow target weight was 636 kg (27 kg) [1,400 lbs (SD 60 lbs)]. Mature bull weight was not tracked in the model.

Body weight for cows and heifers varied with changes in BCS specified for cows and for heifers. BCS differed by time of year based on annual variation related to the reproductive cycle (Table 1). BCS and weight decreased from calving to exposure to bulls due to early lactational demand on stored feed and then increased between bull exposure and pregnancy testing with pasture grazing. BCS and weight also decreased when a cow transitioned to having clinical Johne's disease (Table 1). Johne's associated changes in BCS independently impacted both pregnancy success (Table 1) and final cow cull weights.

### Diagnostic test performance

The sensitivity and specificity values included in the model for serum ELISA, individual fecal PCR and pooled fecal PCR were informed from previous research on diagnostic test performance in western Canadian cow-calf herds (34). For each model run, test sensitivity and specificity values were drawn from a pert distribution (Table 1). The sensitivity of each test for detecting animals that were infected but not yet infectious (latent or silent stage of infection) was set at 0 in the model, as it was very unlikely that these animals were detectable by either blood or fecal tests (Table 1) (1, 40).

### Randomness and stochasticity

Examples of randomness and stochastics in the model included the random assignment of animal age based on a distribution at model initiation and similarly from a specific distribution for purchased animals. The initial infection status of cows and bulls and for purchased animals was also randomly allocated based on previously reported prevalences for each stage of infection (Table 1). Calf sex was randomly assigned at calving as was birth weight from a specified distribution. Survival of the calf to weaning was randomly assigned based on a probability of calf mortality that varied for cows and heifers (Table 1). At pregnancy testing, whether a cow was pregnant or not was assigned randomly based on a probability of pregnancy specific for cows and heifers based on BCS (Table 1).

Stochastics were particularly important in infection transmission and subsequent progression of the infection and clinical disease. Transmission of MAP from dam to calf *in utero* was a random event governed by a probability drawn from a reported distribution (Table 1); preweaning transmission was a random event from infected cows to their calves (Table 1). Whether animals were infected from other fecal transmission and the environment was directly dependent on exponentially distributed rates of infection which varied based on animal age (Table 1) and the dynamic weighted real-time prevalence of infective animals in the herd.

The progression of infection was dependent on a series of time delays where the means were randomly drawn from a series of pert distributions informed by information from the current literature and rates of individual events dependent on exponential distributions informed by the selected mean duration (Table 1). These distributions reflected the uncertainty in the science particularly for beef herds and also the known biological variability based on the initial dose of infectious organisms and age-based susceptibility of the animal at the time of exposure.

### Input data

Model inputs are shown in Table 1. Data included in the model were informed by the current literature, calibration, as well as expert opinion and included factors related to initial herd characteristics, pregnancy, calf weaning weight and mortality, initial herd infection status, transmission risk and diagnostic test performance. Examples of parameters that are similar to those published for other existing ABM models of MAP transmission and control were highlighted (Table 1).

### Key model outputs

Simulated MAP infection prevalence was the key output that emerged from the model and was the result of a combination of initial conditions, testing choices, testing performance and herd replacement decisions. The prevalence of infected (but not yet infectious—latent stage of infection) as well as infective cows (subclinical and clinical or moderate and heavy shedders) >1 year of age was reported each year at the time of testing before culling and at the end of the year.

The number of animals in each production category along with the number of animals born, weaned, sold, retained, infected, and tested at the end of each year were also provided in the model output. Twice-yearly outputs were used in a scenario with no MAP infection to validate that the base model created a cow-calf herd with a population that followed expected seasonal fluctuations, but that maintained a stable population of calving cows with expected production parameters, replacement rates and age distributions. All values were examined over the 10-year time period evaluated in these experiments. The importance of development and validation of the baseline production system model has previously been noted for other ABMs (18).

### Model validation

To further validate that the underlying cow-calf model maintained a stable breeding population over time, weaned the expected number of calves at the expected range of weaning weights, culled the expected number of animals for each reason of interest, and followed expected distributions for mature animal weights and body condition scores, appropriate annual descriptive metrics were generated for the scenario with no MAP infection (Table A in Supplementary material). Selected graphical outputs were also included in the public interactive version of the models to allow the user to verify the implementation of model customizations.

Additional annual metrics reported numbers of animals tested with each method for each scenario, number of animals culled based on test results and for clinical MAP infection, number of true positives appropriately classified by the test, number of true negatives correctly classified by the test, and distribution of animals based on age and stage of infection. Animal weight and BCS were also graphed based on age, time of year and stage of infection.

Animations of individual cows and calves were selected using the graphical user interface during individual model runs to observe their evolution through a simulation with respect to mortality, aging, advancing through the production stages, infection, pregnancy and calving. By clicking on individual animals in the animation it is also possible to see their origin, dam, individual age, reproductive history, list of calves, infection status, weight, and BCS. The animations and live graphics on the graphical user interface serve as an additional option to monitor herd movements and calving, culling, and infection dynamics during each model run.

Finally, a publicly available version of the model was presented to veterinarians and producers through the national producer organizations for input and discussion (52). The model has been through a number of versions based on external input and the consistency of key results demonstrating the robustness of the core structure.

### Model calibration

Automated calibration experiments using the widely implemented OptQuest global optimization routine were created in the commercial software program (AnyLogic version 8.7) to identify the value of the parameter for the frequency of infective contact for fecal transmission of MAP, and the probability of dam to calf transmission from birth to weaning (53–55).

The experiments were used to identify parameter values that best described the longitudinal prevalence data provided by the Saskatchewan Stock Grower's Association (SSGA) for previously confirmed MAP-infected Saskatchewan cow-calf herds. These data were collected as part of a voluntary Johne's Disease Surveillance Program (56) and shared anonymously with permission from the herd owners. The prevalence data were collected over a period of 7 years from 10 cow-calf herds and represented a total of 43 annual complete herd serological testing events on 6,119 samples collected from beef cows >2 years of age (Table 2). The number of cows tested in the first year ranged from 74 to 232 per herd.

The calibration scenarios assumed MAP was introduced into the herd at least 6 years prior to the first test based on herd owner surveys. The reference data were derived from samples tested with a commercial serum ELISA (IDEXX, Westbrook, ME) to detect MAP antibodies in blood from beef cows at least 2 years of age. A sample to positive ratio (S/P) >0.60 was considered evidence of infection. The manufacturer reported test sensitivity and specificity were 68 and 99%, respectively. True prevalence was estimated for the test results using the Rogan and Gladen (43) method with Clopper-Pearson 95% confidence limits in a publicly available calculator (44). The reference data were summarized relative to year 1 of the first sample collection for each herd.

The calibration experiments reflected the reported pattern of testing and culling from the participating herds and were not intended to simulate a baseline with no control measures. The individual herds that shared data from the SSGA program tested between 3 and 6 times during this 7-year period, with 7 of the 10 herds testing 4 times or on average every second year (Figure A in Supplementary material). Five of the participating herds also reported culling daughters of positive dams during this period and this management practice was included in the calibration simulation.

The calibrated parameters resulted from the best fit between the simulated prevalence and the estimated true prevalence reported for the described testing and culling pattern for this group of herds. For determining the simulated prevalence in the calibration experiment resulting from testing and culling, ELISA sensitivity and specificity varied over a pert distribution as did the duration of time spent in each disease state (Table 1). Other distributions used for subsequent scenario comparisons (Table 1) were held fixed at their mean for calibration.

**Figure 3:**
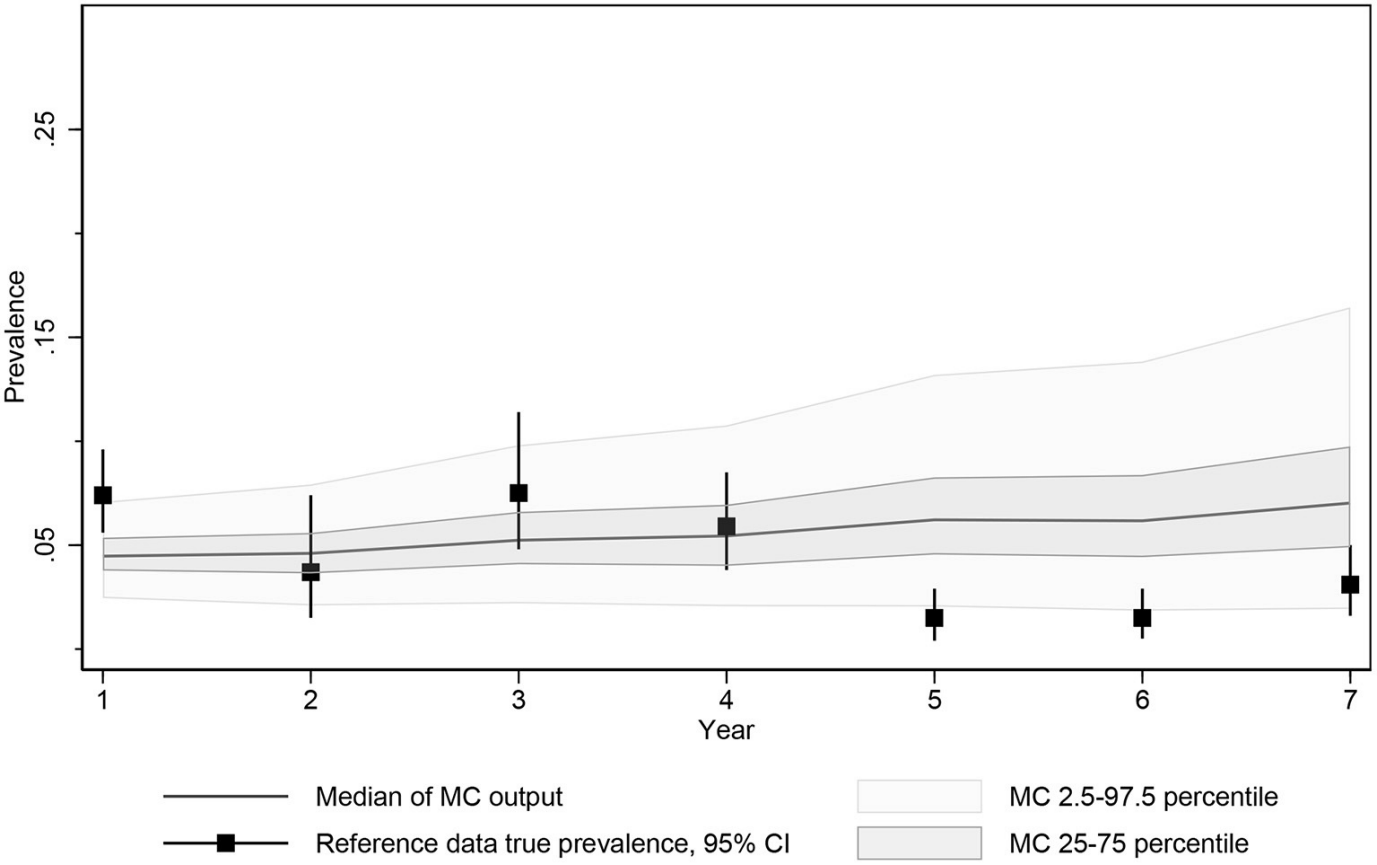
Median, 2.5 and 97.5 percentiles and interquartile range of MC true prevalence simulations for model calibration compared to estimated true prevalence reference data from 10 Saskatchewan cow-calf herds.

The global optimization employed in calibration adjusted the parameter value to minimize an objective function representing the discrepancy between model output and empirical data. Calibration was performed using OptQuest's automated experiment-optimization and was run for 4,000 iterations (each examining a different proposed value for the two target parameters), with 25 realizations per iteration (Figure 3) for a total of 100,000 realizations. Each realization provided an opportunity for unique stochastics and a random draw from other key parameter values with distributions.

The objective function being minimized during calibration consisted of the square root of the sum of the squared differences between the actual simulated prevalence measured at the time of each test and the estimated true prevalence reported by the SSGA program at each test time point. The calibration function was further weighted by doubling the influence of the first and final data points to ensure that the resulting function approached the expected prevalence at the start of the monitoring period and captured any success observed by testing and culling with the program.

### Testing scenarios

**Figure 4:**
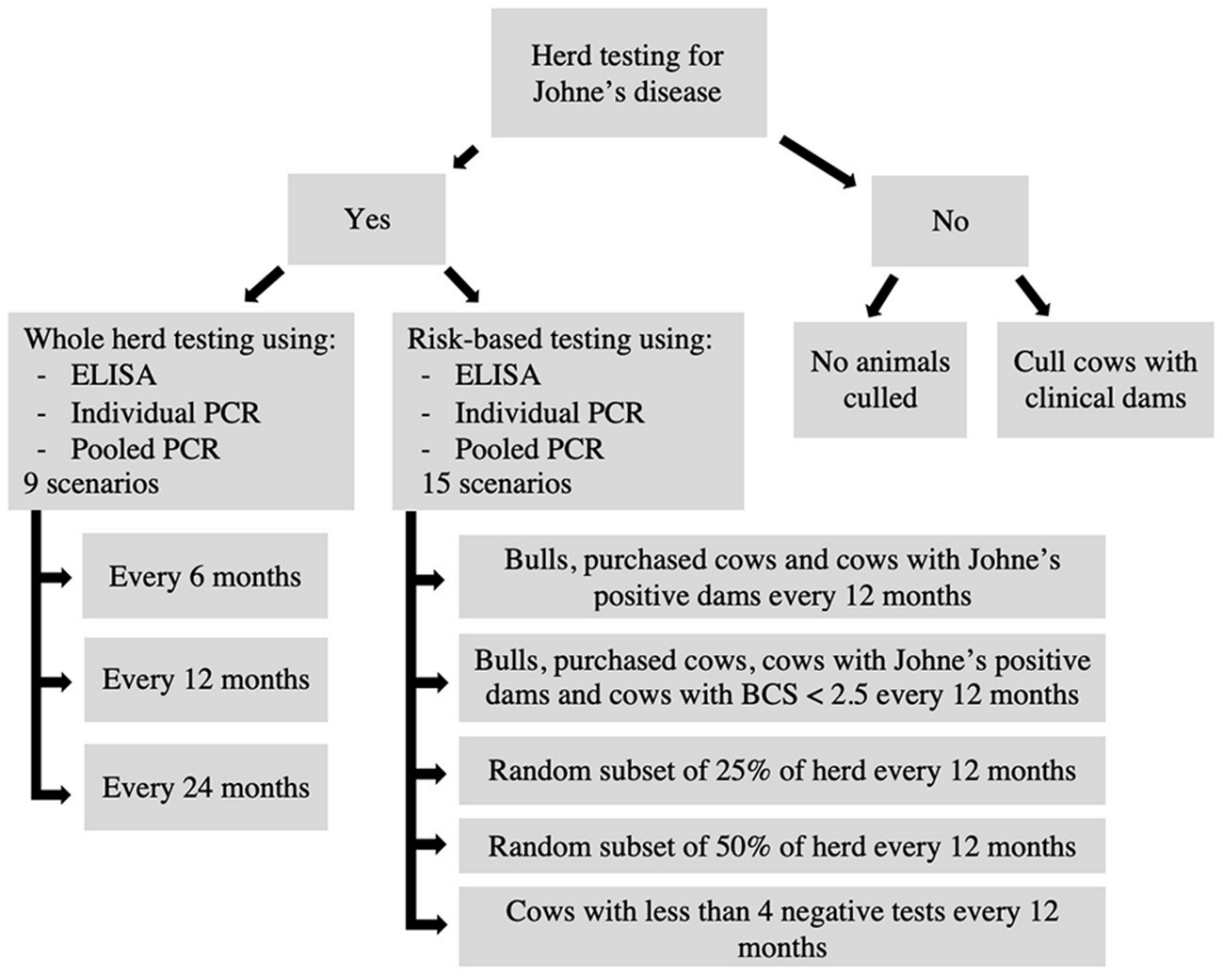
Flow chart outlining the 26 testing scenarios compared using the model.

The testing scenarios evaluated using the model are shown in Figure 4. Each testing scenario consisted of a unique combination of factors, including the decision to test, type of test used, testing frequency, type of animals tested and type of animals culled. General herd characteristics such as herd size and replacement strategy remained the same across all scenarios along with initial infection prevalence and diagnostic test performance (Table 1). In total, 26 testing scenarios were evaluated (Figure 4). A baseline scenario with no testing and no culling was evaluated as well as a scenario with no testing but culling of animals with clinical dams (Figure 4).

Whole herd testing scenarios using serum ELISA, individual fecal PCR and pooled fecal PCR at frequencies of 6, 12, and 24 months were evaluated to compare the impact of testing frequency (Figure 4). A series of risk-based testing scenarios were also evaluated using serum ELISA, individual fecal PCR and pooled fecal PCR at a testing frequency of 12 months (Figure 4). Risk-based scenarios involved testing a subset of animals in the overall herd based on high-risk criteria such as whether the animal was a bull and purchased into the herd or if a cow were purchased, had a Johne's positive dam, or had a BCS <2.5 out of 5. Cattle with fewer than 4 previous negative tests were also identified for testing (57). Other scenarios involved testing a random subset of either 25 or 50% of the herd.

### Analysis of model output

#### Within-herd prevalence of animals with infectious MAP

The simulated prevalence of infectious MAP for those in the subclinical (moderate shedders) and clinical stage (heavy shedders) of MAP after the 10-year period were summarized with the median, 95% prediction interval (2.5th and 97.5th percentiles), and interquartile range (IQR) of 5,000 realizations for each testing scenario. The percentage of realizations with a prevalence of 0% at 10 years was also reported. While these values are not intended to make exact predictions of expected prevalence, they do provide a framework for making relative comparisons among the expected performance of testing scenarios and variability.

The testing scenarios that resulted in at least 75% of the observed infectious prevalence of less that the starting prevalence of 5% after 10 years were selected for further comparison. The median and 2.5 and 97.5 percentiles of the infectious MAP prevalence simulations were graphed over 10 years for each comparison in Stata (StataCorp. 2021. Stata Statistical Software: Release 17. College Station, TX: StataCorp LLC.), and histograms were produced to visually assess the distributions of prevalence after 5 and 10 years for the various scenarios.

#### Cost of testing

The annual testing cost for each scenario was calculated using the mean number of animals tested per year for 5,000 realizations of the model output as well as a sample collection fee and a laboratory sample processing fee per animal. The sample collection fee was set at $5.00 CAN per head for blood samples and $2.50 CAN per head for fecal samples and covered expenses for veterinary service, supplies and courier costs.

The sample processing fees were based on the regional diagnostic service laboratory fees for Johne's disease testing. Serum ELISA tests cost $13.50 CAN per sample for submissions with fewer than 90 samples and $10.50 CAN per sample for submissions with 90 or more samples. Individual fecal PCR tests cost $46.00 CAN per sample for fewer than 100 samples and $37.50 CAN for 100 or more samples. Fecal PCR testing in pools of five samples cost $62.50 CAN plus the cost of retesting individual samples from positive pools.

Yearly testing cost per infective prevalence reduction was calculated by dividing the yearly testing cost by the initial prevalence of infective cows minus the 10-year infective prevalence.

#### Sensitivity analysis

Additional scenarios were examined as part of a sensitivity analysis to evaluate the impact of starting prevalence, prevalence in purchased cattle, and transmission parameters on the relative performance of serum ELISA, individual fecal PCR, and pooled fecal PCR with testing once per year. Parameters chosen for additional scenarios were either calibrated values or values that had the potential to very influential on the final prevalence and that were held constant to facilitate calibration and communication of model findings. The range of values examined was informed by the literature (34, 36, 58), expert opinion, and prior models (5, 21). The 10-year simulated prevalence of cattle with infectious MAP for these scenarios were reported with medians and 95% prediction intervals (2.5th−97.5th percentiles) to demonstrate any differences in the trend across the primary testing options for extreme values of tested parameters. While not a traditional global sensitivity analyses, these comparisons were not strictly one-way sensitivity analyses as they concurrently reflected variability in the predicted prevalence associated with all other parameters represented as distributions in the model (Table 1).

Spider and tornado plots were created using R (R Foundation for Statistical Computing, Vienna, Austria) to determine the sensitivity of the mean predicted 10-year prevalence to variability in all model inputs represented by distributions (Table 1) for whole herd testing scenarios with each diagnostic test at a frequency of 12 months. These plots demonstrate the variability in the mean prevalence conditional on other inputs when an input is held to fixed values, allowing assessment of the relative influence of important inputs on the model output. The center of the graphs represents the expected value of the simulated MAP prevalence when all inputs are at their mean. Model inputs most likely to influence the probability of MAP transmission and progression within individual animals and diagnostic test performance were included: probability of *in utero* transmission for subclinical and clinical cows, relative susceptibility of postweaning calves and adults, duration of latent and subclinical disease stages, the time from detection of clinical signs until removal from the herd, and diagnostic test specificity and subclinical and clinical sensitivity for each test type.

The sensitivity of PCR was highlighted as area of particular uncertainty in the literature (59). Given that the original study from which the currently used value was derived was based on a relatively small number of PCR positive cows from infected herds (34), a separate analysis was undertaken. This analysis included the only other previously reported sensitivity values for the commercial PCR test used in the regional laboratory (VetAlert Johne's Real-Time PCR kit (Tetracore, Rockville, MD): 77.6% (95%CI 73.2–82.0) (60) and 60–72% (61) Simulation results from this second pert distribution of 77.6% (60%–82%) were compared to those from Table 1.

## Results

### Model calibration

True prevalence estimates were summarized from 10 MAP-positive Saskatchewan cow-calf herds reporting serum ELISA testing, typically every 2 years, as part of a provincial control program using data for up to 7 years from the start of testing and culling (Table 2, Figure A in Supplementary material). True prevalence estimates ranged from 1.5 to 7.5%.

Of the automated calibration-optimization scenarios examined, the resulting best fit frequency of infective contacts per year was 46.89 and probability of dam to calf transmission from birth to weaning was 0.577. The estimates of simulated prevalence of infectious animals resulted from the scenario allowing for limited adult transmission and were identified at iteration 3,024 from a total of 4,000 completed calibration iterations and 100,000 individual simulations (25 repeated simulations/completed calibration).

The median prevalence of the Monte Carlo output generated by these calibrated parameters resulted in a predicted prevalence of 5.0% (25th−75th percentile, 3.4–7.2%) after testing and culling in year 7 reflecting the limited control expected from an imperfect approximately biannual test and culling program. Given that the date of MAP introduction was unknown and the testing and culling histories for individual herds varied slightly during this period, the difficulty in matching the exact average of the herds testing in this year was not unexpected, but the 95% confidence intervals of the true prevalence estimated from the reported data at the time of testing and 95% prediction intervals for the calibrated model overlapped for all 7 years (Figure 3).

### Predicted prevalence of infectious map (subclinical moderate shedders and clinical heavy shedders)

**Figure 5:**
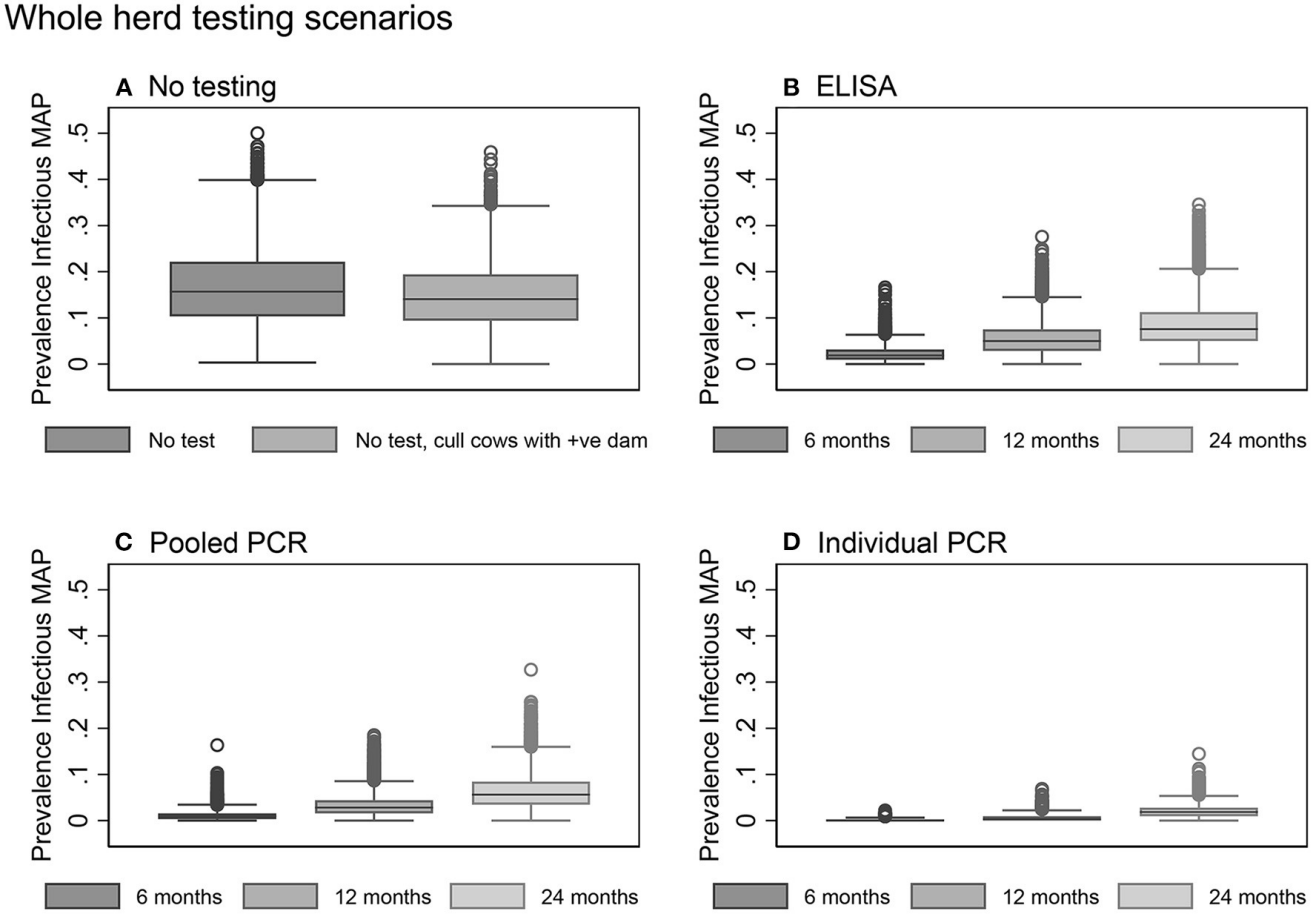
Box plots showing the within-herd prevalence after 10 years for baseline scenarios **(A)** and whole herd testing scenarios using serum ELISA **(B)**, pooled fecal PCR **(C)**, and individual fecal PCR **(D)**.

The mean herd prevalence tripled from 5.0% at the start of the model to year 10 with no testing or culling to control Johne's disease (Table 3). Not testing the herd but culling daughters of clinical dams was found to be slightly more effective than no testing and no culling (Table 3, Figure 5A).

**Table 3:** Median for 10-year prevalence for 5,000 realizations of the model output, percentage of realizations with a 0% 10-year prevalence, median number of animals tested per year, median testing cost per year (and 95% prediction intervals), and median yearly testing cost per unit decrease in prevalence.

**Testing scenario**	Median 10-year infective prevalence (%) (Percentiles 2.5^th^–97.5^th^) (IQR)**	**Percentage of realizations 10-year infective prevalence = 0.0%**	**Median number of animals tested per year (Percentiles 2.5^th^–97.5^th^)**	**Median cost of testing per year (Percentiles 2.5^th^–97.5^th^)**	Median yearly testing cost per unit of reduction in infective prevalence***
No MAP infection (validation control)	0.0	100%	0.0	0.0	N/A
Individual PCR whole herd 6 months	0 (0, 0.9) IQR (0, 0.3)	58%	478 (469, 485)	$19,100 (18,768, 19,416)	$3,856
Individual PCR whole herd 12 months	0.3 (0, 2.5) IQR (0, 0.9)	26%	241 (236, 246)	$9,656 (9,456, 9,836)	$2,086
Pooled PCR whole herd 6 months	0.9 (0, 4.4) IQR (0.3, 1.6)	11%	99 pools (97, 100) 77* (42, 127)	$10,770 (9,314, 12,271)	$2,623
Individual PCR cows with < 4 negative tests	1.3 (0, 4.1) IQR (0.6, 1.9)	6.1%	187 (182, 193)	$7,484 (7,264, 7,720)	$1,963
ELISA whole herd 6 months	1.9 (0, 7.3) IQR (0.9, 3.1)	2.9%	474 (466, 482)	$7,349 (7,218, 7,469)	$2,144
Individual PCR whole herd 24 months	1.9 (0.3, 5.9) IQR (0.9, 3.4)	2.0%	118 (114, 121)	$4,700 (4,564, 4,824)	$1,379
Pooled PCR whole herd 12 months	2.8 (0.3, 10.7) (IQR 1.6,4.4)	1.3%	52 pools (51, 53) 53* (28, 85)	$6,297 (5,136, 7,593)	$1,896
Individual PCR 50% of herd	3.1 (0.3, 10.7) IQR (1.9, 5.3)	0.8%	119 (114, 125)	$4,804 (4,596, 5,016)	$1,469
Pooled PCR cows with < 4 negative tests	3.8 (0.6, 12.3) IQR (2.2, 6)	0.4%	43 pools (41, 44) 44* (24, 68)	$5,186 (4,241, 6,354)	$1,549
ELISA whole herd 12 months	5 (0.9, 15.7) IQR (2.8, 7.5)	0.3%	238 (232, 243)	$3,691 (3,596, 3,763)	$769
Pooled PCR whole herd 24 months	5.6 (0.9, 16) IQR (3.4, 8.5)	0.2%	25 pools (25, 26) 31* (17, 47)	$3,324 (2,684, 3,882)	$-418
ELISA cows with < 4 negative tests	6.3 (1.3, 17) IQR (3.8, 9.4)	0.1%	186 (181, 192)	$2,885 (2,801, 2,970)	$-421
Pooled PCR 50% of herd	6.9 (1.3, 19.7) IQR (4.4, 10.6)	0%	26 pools (25, 27) 33* (18, 51)	$3,463 (2,749, 4,307)	$-511
ELISA whole herd 24 months	7.6 (1.6, 20.1) IQR (5, 11.3)	0.1%	116 (113, 120)	$1,804 (1,745, 1,855)	$-287
Individual PCR 25% of herd	7.6 (1.6, 20.8) IQR (4.7, 11.3)	0.1%	59 (55, 63)	$2,857 (2,648, 3,070)	$-447
ELISA 50% of herd	9.1 (1.9, 23.7) IQR (6, 13.5)	0%	118 (112, 123)	$1,826 (1,738, 1,913)	$-257
Pooled PCR 25% of herd	10.7 (2.2, 26.3) IQR (6.9, 15.5)	0.1%	13 pools (12, 14) 18* (9, 28)	$1,815 (1,390, 2,272)	$-225
Individual PCR high risk + low BCS animals^†^	11 (2.2, 24.8) IQR (7.2, 15.6)	0%	59 (43, 78)	$2,846 (2,076, 3,526)	$-354
Individual PCR high risk animals^†^	11.9 (2.5, 26) IQR (7.8, 16.9)	0%	54 (37, 72)	$2,580 (1,804, 3,299)	$-284
ELISA 25% of herd	12.2 (2.5, 28.9) IQR (7.9, 17.2)	0%	59 (54, 63)	$1,082 (1,001, 1,164)	$-122
Pooled PCR high risk + low BCS animals^†^	12.5 (2.8, 28.8) IQR (8.2, 17.7)	0%	14 pools (10, 18) 17* (7, 32)	$1,813 (1,183, 2,789)	$-202
Pooled PCR high risk animals^†^	13.2 (2.5, 29.5) IQR (8.8, 18.5)	0%	12 pools (8, 16) 14* (5, 29)	$1,526 (928, 2,452)	$-159
ELISA high risk + low BCS animals^†^	13.3 (2.8, 30) IQR (8.8, 18.6)	0%	59 (43, 78)	$1,068 (797, 1,312)	$-107
ELISA high risk animals^†^	13.9 (2.8, 30.7) IQR (9.1, 19.4)	0%	54 (37, 73)	$979 (688, 1,234)	$-95
No testing but culling cows and heifers with clinical dams	14.1 (3.1, 30.8) IQR (9.4, 19.4)	0%	0	$0	NA
No testing or culling	15.7 (3.8, 36.1) IQR (10.3, 22.2)	0%	0	$0	NA

* Number of samples in positive fecal pools retested individually with PCR.

† High risk animals include bulls, purchased females and females with Johne's disease positive dams.

** IQR—interquartile range (25^th^−75^th^ percentile).

*** A negative value indicates cost per unit of prevalence increased during the testing period.

Overall, testing scenarios involving the use of individual PCR resulted in a lower 10-year within-herd prevalence compared to the same scenarios involving the use of pooled PCR or ELISA (Table 3, Figure 5).

Whole herd testing with individual fecal PCR every 6 or 12 months were the most effective options, achieving median 10-year prevalences of 0.0 and 0.3%, respectively (Table 3, Figure 5D). Whole herd testing every 6 months with individual PCR resulted in 58% of the 10-year prevalence realizations of 0%; 26% of realizations were 0% for whole herd testing with individual PCR every 12 months (Table 3). Testing the whole herd every 24 months with individual PCR also reduced MAP prevalence in most realizations (Table 3, Figure 5D).

Whole herd testing every 6 or 12 months using pooled fecal PCR resulted in a decreased prevalence after 10 years to 0.9 and 2.8%, respectively (Table 3, Figure 5C).

Whole herd testing with serum ELISA every 6 months reduced the median MAP prevalence after 10 years to 1.9%, and testing every 12 months held it steady at 5.0%. Whereas, with ELISA testing at a frequency of 24 months, prevalence increased to 7.6% after 10 years (Table 3, Figure 5B).

**Figure 6:**
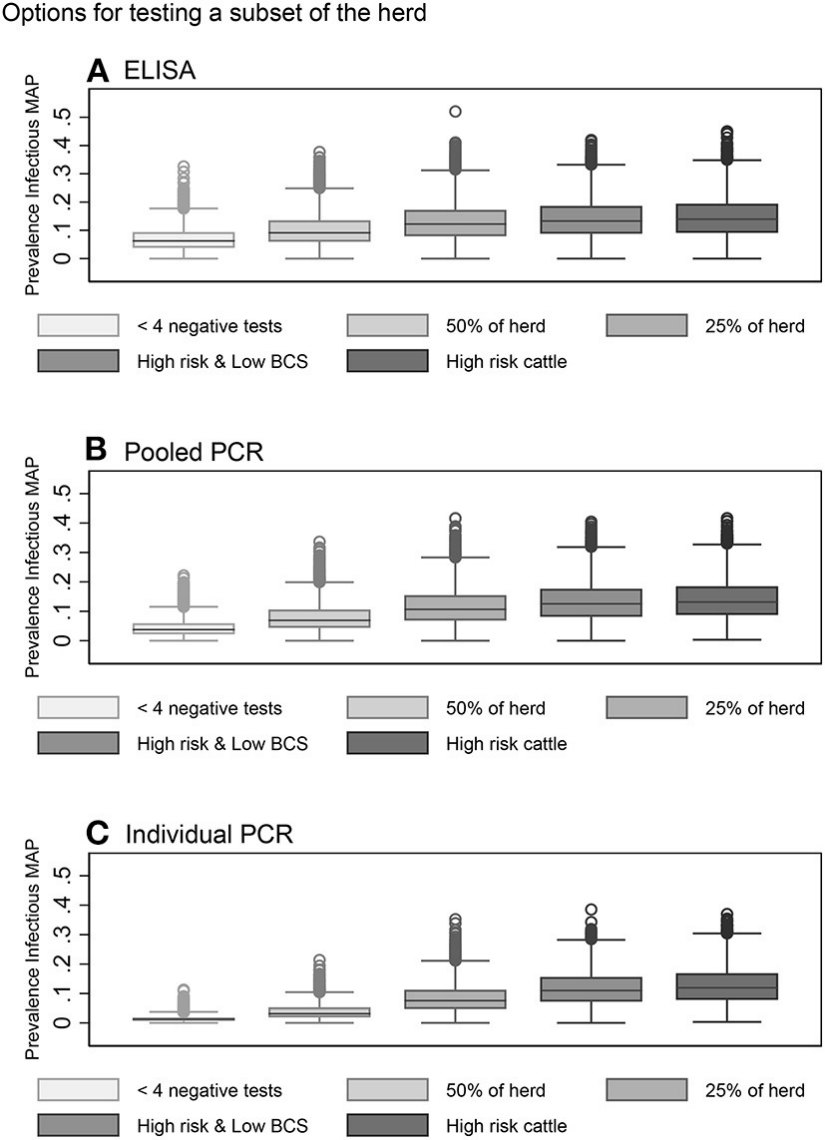
Box plots showing the within-herd prevalence after 10 years for risk-based testing scenarios using serum ELISA **(A)**, pooled fecal PCR **(B)** and individual fecal PCR **(C)**.

In general, risk-based testing scenarios were less effective than whole herd testing scenarios. Restricted testing of cows with fewer than 4 negative test results every 12 months was the most effective risk-based testing scenario across all three diagnostic tests (Table 3, Figures 6A–C). The mean 10-year infective prevalence (1.3%) and realizations with 0% 10-year infective prevalence (6.1%), was fourth best of all testing and culling options examined.

Testing a random subset of 50% of the herd every 12 months with individual PCR also resulted in a decrease in within-herd prevalence for more than half of realizations after 10 years (Table 3, Figure 6C). Testing a random subset of 25% of the herd, high risk animals including bulls, purchased cows and cows with positive dams, and high-risk animals in addition to animals with low BCS were the least effective scenarios at reducing disease prevalence across all three diagnostic tests (Table 3, Figures 6A–C).

**Figure 7:**
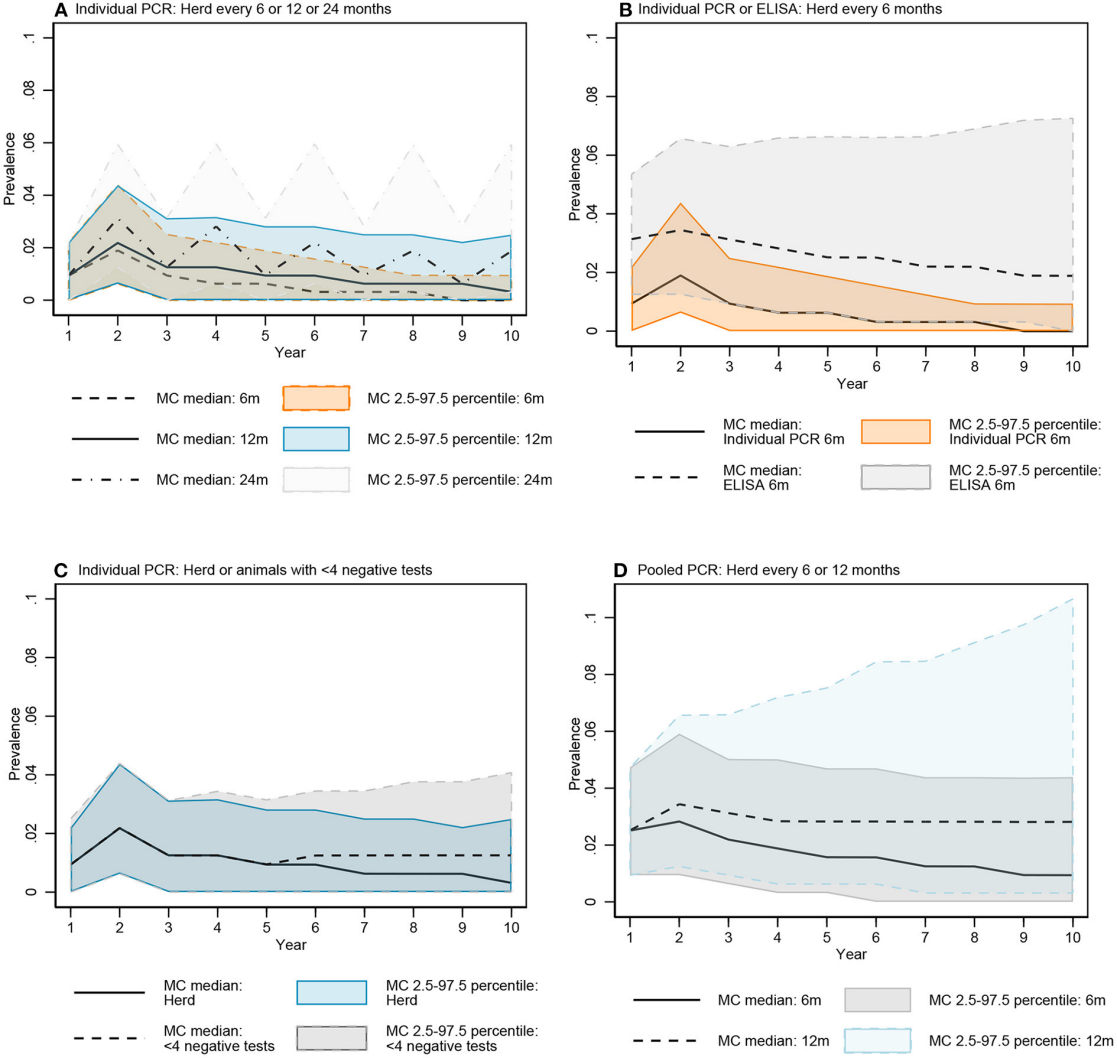
Comparisons of the simulated MAP prevalence over time for key testing scenarios: Individual fecal PCR testing every 6, 12, and 24 months **(A)**, individual PCR and ELISA every 6 months **(B)**, individual fecal PCR for the whole herd and animals with <4 negative tests every 12 months **(C)**, and pooled fecal PCR every 6 and 12 months **(D)**. Data represent simulated MAP prevalence after testing and culling model processes for each year.

**Figure 8:**
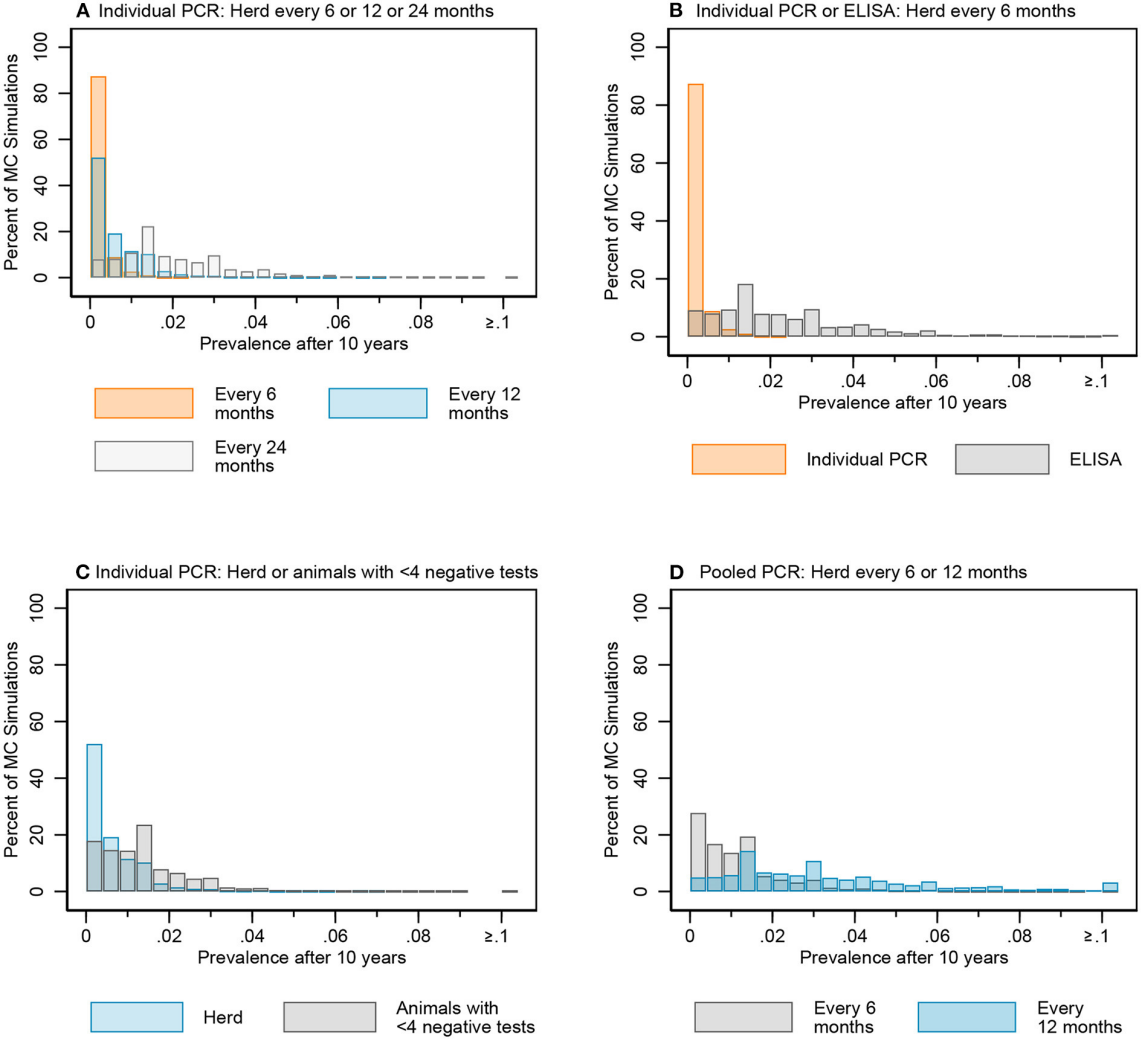
Distribution of simulated infectious MAP prevalence after 10 years for key testing scenarios: individual fecal PCR testing every 6, 12, and 24 months **(A)**, individual PCR and ELISA every 6 months **(B)**, individual fecal PCR for the whole herd and animals with <4 negative tests every 12 months **(C)**, and pooled fecal PCR every 6 and 12 months **(D)**.

The median of the Monte Carlo (MC) prevalence simulations for whole herd testing scenarios with individual PCR every 6 and 12 months followed a similar trend over the 10-year testing period (Figure 7A). There was a greater difference between prevalence in MC prevalence simulations for 6- and 12-month testing frequency at year 10 compared to year 5 (Figure 8A, Figure B in Supplementary material).

The median prevalence for whole herd testing every 24 months with individual PCR was higher and had more variability over the 10-year period compared to its 6- and 12-month frequency comparators (Figure 7A). There was again more overlap in MC simulations at year 5 compared to year 10 (Figure 8A, Figure B in Supplementary material).

The median MC prevalence was lower for testing every 6 months with individual PCR compared to ELISA (Figure 7B) over the 10-year testing period. Similarly, there was greater overlap in simulated MC prevalence at year 5 compared to year 10 (Figure 8B, Figure B in Supplementary material).

In the comparison between whole herd testing with individual PCR every 12 months and restricted individual PCR testing of cows with fewer than 4 negative tests every 12 months, the median prevalence for testing cows with fewer than 4 negative tests at year 5 increased above that of whole herd testing and remained higher for the duration of the 10-year period (Figure 7C). Similar to the comparison of the previous scenarios, the overlap was higher at year 5 vs. year 10 (Figure 8C, Figure B in Supplementary material).

The median prevalence for whole herd testing with pooled PCR every 6 months was lower than that of whole herd testing with pooled PCR every 12 months throughout the duration of the 10 years, and declined over that time while prevalence for every 12-month pooled PCR testing remained relatively stable (Figure 7D). The percentage of overlap was highest in the middle of the bar graph, with the 6-month frequency scenario more often having a lower prevalence compared to the 12-month scenario at both 5 and 10 years (Figure 8D, Figure B in Supplementary material).

### Yearly testing cost

Whole herd testing with individual fecal PCR at a frequency of 6 months was the most expensive testing scenario, followed by whole herd testing with pooled PCR at a frequency of 6 months (Table 3). Whole herd testing with individual PCR every 12 months was the third most expensive testing scenario (Table 3). Testing high risk animals and high risk in addition to low BCS animals every 12 months were the least expensive testing scenarios overall across all diagnostic tests (Table 3).

### Yearly testing cost per unit of infective prevalence reduction

Of the 7 scenarios that resulted in decreased prevalence at least 75% of the time over 10 years, testing every 24 months with individual PCR had the lowest yearly testing cost per unit of infective prevalence reduction (Table 3). Whole herd testing with pooled PCR every 12 months had the second lowest yearly cost per infective prevalence reduction, followed by restricted testing of cows with <4 negative tests every 12 months with individual PCR (Table 3).

### Sensitivity analysis

Changing the initial within-herd prevalence or the expected prevalence of infectious as well as latent animals purchased to levels expected in infected herds did not change how the diagnostic tests impacted herd prevalence at 10 years relative to each other (Table 4). Because most replacements were home raised in the tested scenarios, as would be expected in most western Canadian commercial herds, the impact of increasing the risk in incoming animals on the final prevalence was less than altering transmission risks or initial conditions.

**Table 4:** Median 10-year infective prevalence for 5,000 realizations of the model output for whole herd testing scenarios every 12 months with each of the diagnostic tests varying initial prevalence of MAP, prevalence of MAP in purchased cattle, and calibrated transmission parameters.

**Baseline testing scenarios compared to alternatives**	**Mean 10-year infective prevalence (%) (Percentiles 2.5^th^−97.5^th^)**
Initial percent of cattle infected (latent)−5% and initial prevalence of cattle subclinical (infectious)−5%*	
No testing−1% initial prevalence	5 (0.3, 19.8)
ELISA−1% initial prevalence	1.6 (0, 7.2)
Pooled PCR−1% initial prevalence	0.6 (0, 4.7)
Individual PCR−1% initial prevalence	0 (0, 1.3)
No testing−10% initial prevalence	23.3 (6.6, 43.3)
ELISA−10% initial prevalence	8.2 (2.2, 19.8)
Pooled PCR−10% initial prevalence	4.7 (0.9, 14.1)
Individual PCR−10% initial prevalence	0.9 (0, 3.5)
Initial percentage of purchased cattle infected (latent)−1% and initial percentage of subclinical (infectious) cattle−1%*	
No testing−5% prevalence purchased cattle	17.6 (4.4, 37.7)
ELISA−5% prevalence purchased cattle	6 (1.3, 17)
Pooled PCR−5% prevalence purchased cattle	3.5 (0.6, 12)
Individual PCR−5% prevalence purchased cattle	0.6 (0, 3.1)
No testing−10% prevalence purchased cattle	19.7 (5.7, 40.4)
ELISA−10% prevalence purchased cattle	7.6 (2.2, 19.4)
Pooled PCR−10% prevalence purchased cattle	4.4 (0.9, 14.1)
Individual PCR−10% prevalence purchased cattle	0.9 (0, 4.1)
**Frequency of infective contact—calibrated parameter (46.89/year)** *	
No testing−2 times transmission risk	34.3 (15.4, 54.2)
ELISA−2 times transmission risk	18.6 (7.5, 31.8)
Pooled PCR−2 times transmission risk	13.9 (4.7, 25.6)
Individual PCR−2 times transmission risk	4.7 (0.6, 11.6)
No testing—½ times transmission risk	3.8 (0.3, 12.2)
ELISA—½ times transmission risk	0.9 (0, 3.5)
Pooled PCR—½ times transmission risk	0.3 (0, 2.2)
Individual PCR—½ times transmission risk	0 (0, 0.6)
Probability of dam to calf transmission before weaning—calibrated parameter (0.577)*	
No testing—high dam to calf transmission (0.95)	18.3 (4.4, 40)
ELISA—high dam to calf transmission (0.95)	6 (0.9, 17.9)
Pooled PCR—high dam to calf transmission (0.95)	3.4 (0.3, 13.8)
Individual PCR—high dam to calf transmission (0.95)	0.6 (0, 3.2)
No testing—low dam to calf transmission (0.25)	14.1 (2.8, 32.8)
ELISA—low dam to calf transmission (0.25)	4.4 (0.6, 13.5)
Pooled PCR—low dam to calf transmission (0.25)	2.2 (0.3, 8.5)
Individual PCR—low dam to calf transmission (0.25)	0.3 (0, 1.9)

* Baseline results for testing scenarios reported in Table 3.

Changing the rate of horizontal transmission from infectious cows to other animals had a greater impact on herd prevalence at 10 years than changing the probability of transmission from an infectious dam to her calf (Table 4). However, in all scenarios the relative ranking of the impact of the diagnostic tests on predicted herd prevalence was consistent.

**Figure 9:**
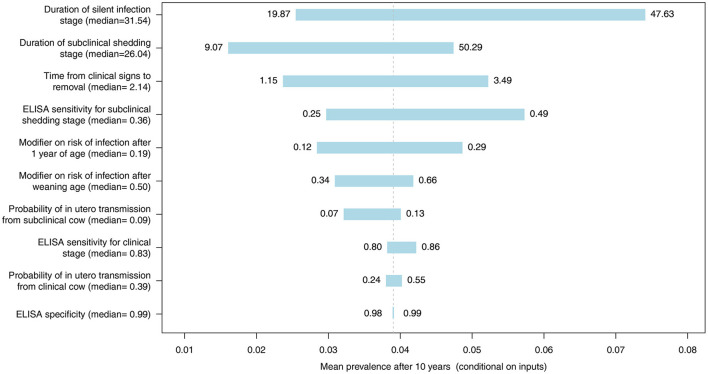
Tornado plot demonstrating the influence of all model inputs represented by distributions on the variability in the mean MC simulated prevalence after 10 years of whole herd testing using ELISA. Inputs included the probability of *in utero* transmission to calves from clinical and subclinical cows, relative susceptibility of postweaning calves and adults, ELISA specificity, ELISA sensitivity for the subclinical shedding and clinical stages, the duration of the silent and subclinical shedding stages (months), and the time from detection of clinical signs to removal from the herd (months). Labels on bars represent the lower (1st percentile) and upper (99th percentile) values of model inputs used in the sensitivity analysis.

**Figure 10:**
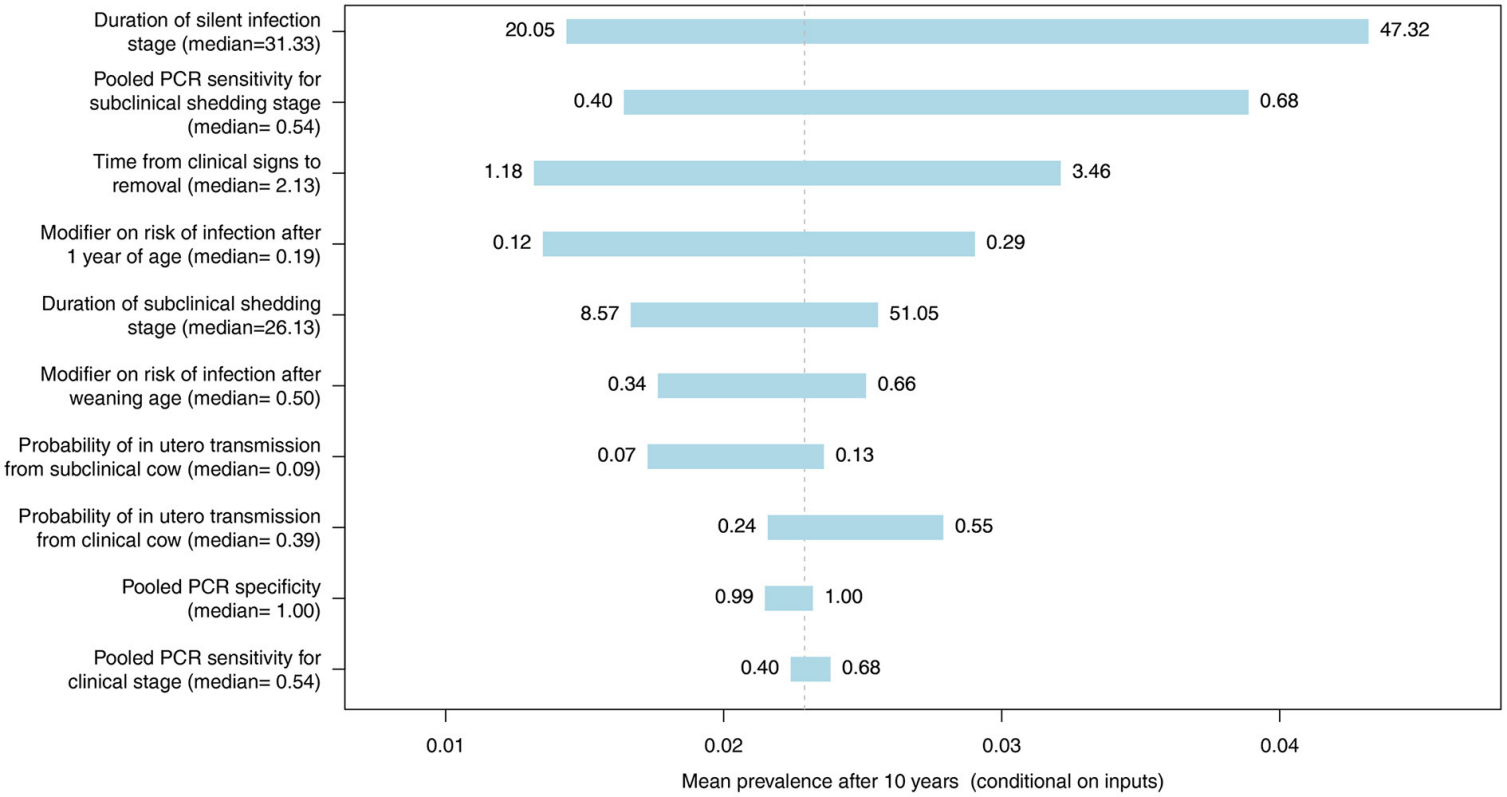
Tornado plot demonstrating the influence of all model inputs represented by distributions on the variability in the mean MC simulated MAP prevalence after 10 years of whole herd testing with pooled PCR every 12 months. Inputs included the probability of *in utero* transmission to calves from clinical and subclinical cows, relative susceptibility of postweaning calves and adults, pooled PCR specificity, pooled PCR sensitivity for the subclinical shedding and clinical stages, the duration of the silent and subclinical shedding stages (months), and the time from detection of clinical signs to removal from the herd (months). Labels on bars represent the lower (1st percentile) and upper (99th percentile) values of model inputs used in the sensitivity analysis.

**Figure 11:**
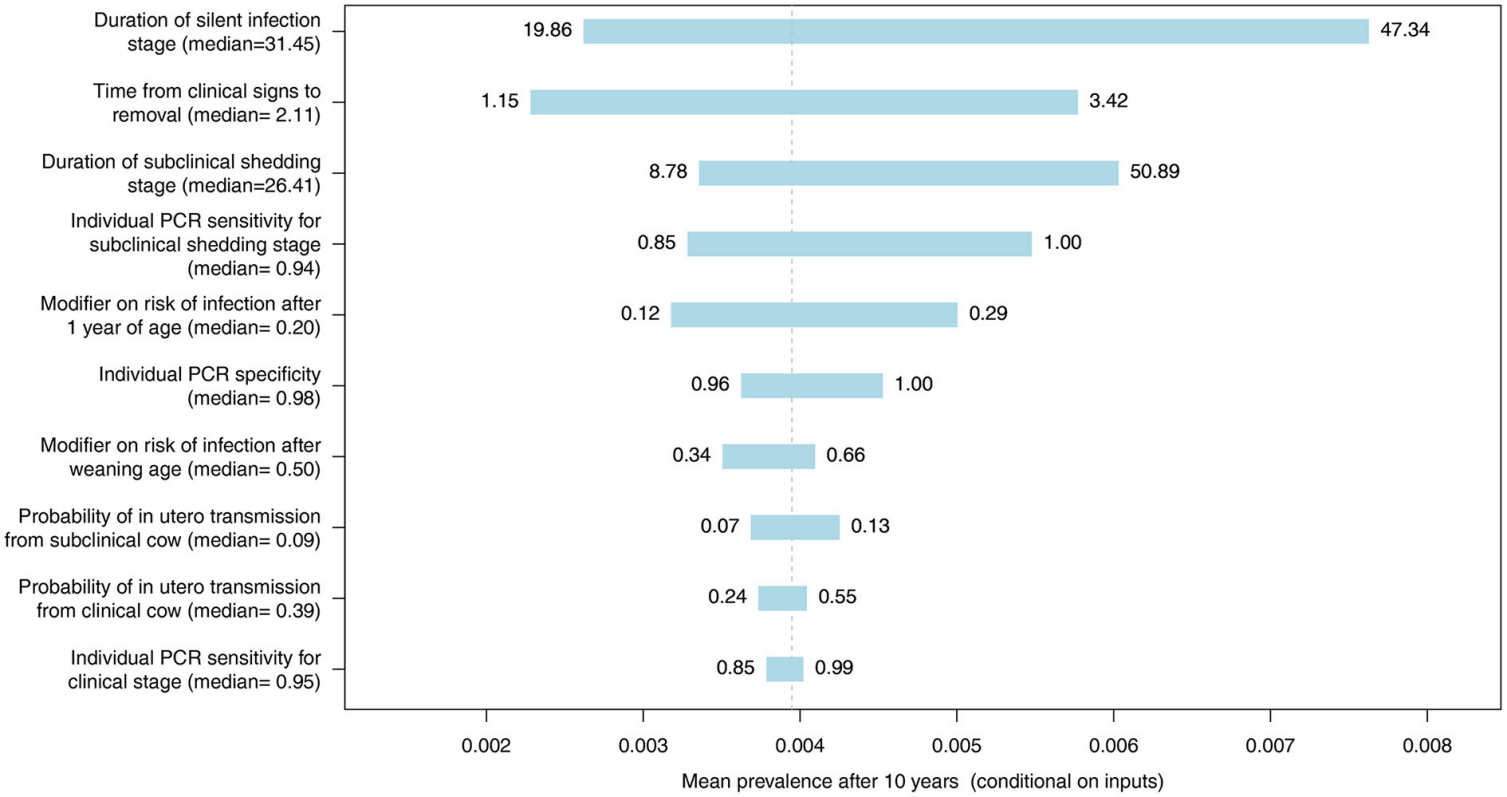
Tornado plot demonstrating the influence of all model inputs represented by distributions on the variability in the mean MC simulated MAP prevalence after 10 years of whole herd testing with individual PCR. Inputs included the probability of *in utero* transmission to calves from clinical and subclinical cows, relative susceptibility of postweaning calves and adults, individual PCR specificity, individual PCR sensitivity for the subclinical shedding and clinical stages, the duration of the silent and subclinical shedding stages (months), and the time from detection of clinical signs to removal from the herd (months). Labels on bars represent the lower (1st percentile) and upper (99th percentile) values of model inputs used in the sensitivity analysis.

The most influential parameter, of those where distributions were used in the model, was the duration of silent infection regardless of which diagnostic test was used (Figures 9–11, Figures C–E in Supplementary material). The next four most influential were the duration of shedding, time from clinical signs to removal, coefficient modifying the susceptibility of animals >1 year of age, and the sensitivity of the diagnostic test during the subclinical stage (Figures 9–11, Figures C–E in Supplementary material). However, the relative ranking of all of these parameters varied between the ELISA, individual PCR and pooled PCR tests.

The result for the scenario where individual PCR with a sensitivity 78% (60–82%) was used once per year for 10 years was a median of 0.9 (2.5th percentile 0.0, 97.5th percentile 4.4; IQR, 0.6, 1.9). This was slightly higher than baseline estimate of 0.3 (0, 2.5) with the regional sensitivity estimate (Table 3). However, it was almost exactly equivalent to the next best performing test, the pooled PCR at every 6 months, and the IQR did not overlap with that of the pooled PCR used every 12 months.

## Discussion

The agent-based simulation model presented here provides a tool to inform the decision to test and cull to control Johne's disease in western Canadian beef cow-calf herds. Several studies have used ABMs to examine options for management of Johne's disease in dairy herds (18–24, 26), but most have been reported within the last decade (16, 18–20, 22–24, 26). Earlier models were primarily compartmental and deterministic in design (16, 18–20, 22–24, 26, 48), with the exception of Kudahl et al. (21). While some of these ABMs examine transmission pathways (18, 22, 25, 26), others have evaluated control measures (18–24, 37). Testing costs and associated impacts of MAP on herd productivity were also considered by some (19, 20, 22, 23). Two dairy ABMs examined more than one type of testing (22, 23). No models considered PCR tests. Only two identified ABMs compared different frequencies for serum ELISAs (22, 24). Other strategies for milk ELISAs were also reported for targeted testing based on animal parity, number of previous positive tests, days in milk and whether the dam was positive (23, 25). The current model in addition to depicting extensively managed cow-calf herds, provides a unique concurrent examination of a range of testing types, frequencies and risk-based testing options.

The present model reflects a typical western Canadian cow-calf herd with respect to herd size, structure and management practices. Previous dynamic simulation models for Johne's disease in beef herds reflect production practices that are common in European beef herds (7, 17, 62). There are differences between the smaller, relatively more intensively managed beef herds found in most European countries and the extensively managed herds from North America targeted in this model (17, 48). The need for region specific models for MAP control in dairy herds has been previously reported (24, 37). There are also distinct differences in the structure and management of beef and dairy herds that must be accounted for in model design, as well as most likely pathways for MAP transmission.

Beef cattle in western Canada and most areas of the western United States are typically managed almost exclusively outdoors, grazed on extensive pastures or cover crops reducing the opportunity for focused areas of environmental contamination that can be readily modeled or targeted through management interventions. Calving occurs seasonally in late winter and spring in most areas of North America and typically outside on pasture or in paddocks. While the calving area is a focus of concern for control efforts (4, 12), the opportunities to precisely model a single meaningful environmental reservoir are limited. In contrast, most dairy herds are managed indoors for at least some part of the year, and most dairies in Europe and North America calve year-round. Many use individual animal calving pens creating focal points for environmental transmission. One recent model of MAP transmission in Irish dairy herds that did consider the impact of compact spring calving (37) highlighted the differences from year-round calving and the need for testing and culling prior to calving to reduce exposure to highly infectious cows present during the calving season. Previous research in beef cattle had identified Johne's disease suspect animals in the calving area as a strong risk factor for herd status (63).

The challenges of obtaining data for modeling MAP in beef herds have been previously recognized (17). Data from recent regional observational studies in cow-calf herds were used in the current cow-calf model to inform many input parameters. For example, the initial within-herd prevalence as well as the initial prevalence in purchased animals were based on a 2019 cross-sectional study to estimate the prevalence of Johne's disease in Canadian cow-calf herds (34). Johnson, McLeod (34) also used the Bayesian latent class model technique to estimate the sensitivity and specificity of ELISA, individual fecal PCR and pooled fecal PCR in Canadian cow-calf herds. The resulting estimates were used as input parameters in the current study. Although previous studies used other data from the literature to inform model input parameters, the data was commonly older, derived from parameter estimates reported by previous models, or not specific to the population of interest or the precise test protocols targeted by the model (7, 17, 22, 24, 25, 37, 64). The present model integrates current and emerging research and surveillance data to inform Johne's disease management and control decisions on commercial cow-calf operations for western Canada; however, the parameters can be changed as necessary to adapt the model for other regions.

Inherent stochastics and the distributions included for parameter values reflect the roles of chance, inherent biological variation and uncertainty due to limitations in the existing research that could impact predictions of MAP transmission, disease progression in individual animals and diagnostic test performance. For example, the rate of MAP transmission from direct fecal transmission or the environment was sampled from an exponential distribution for individual events within a simulation. The probability of *in utero* infection was drawn from a pert distribution, as was the relative susceptibilities of calves after weaning and animals more than 1 year of age. Disease progression between stages of infection and disease was based on time delays randomly drawn from pert distributions. Transitions were implemented as stochastic processes for each animal where the selected value was the mean of an exponential distribution. Diagnostic test sensitivity and specificity values were also randomly drawn from distributions for each simulation. Furthermore, the assignment of animal age and infection status at model initialization were random, as were calf sex and calf survival to weaning. Pregnancy status was an emergent combination of a random probability and the impact of Johne's disease on body condition score.

One beef model and most other previous dairy ABMs examining control options reported elements of randomness and stochasticity as well. Humphry, Stott (17) described stochastics in transmission. The model by Robins, Bogen (22) described age and disease at initialization, successful calving, mortality, MAP transmission and progression and diagnostic test performance as stochastic or random processes. Other models discussing sources of stochasticity also note random initial starting conditions and stochastics associated with transmission rates (24, 25, 37). The present model is somewhat unique in that no other models were identified that incorporated uncertain parameter values as distributions in all simulations. Rather most addressed uncertainty in important values through targeted local and global sensitivity analyses.

While many of the previous models used similar types of epidemiological input parameters to the current model, there were also some key differences. Most identified differences from other models were in pathways for fecal exposure to the calf. The current model captured vertical transmission from dam to calf *in utero* as well as via fecal-oral transmission either directly from the dam or indirectly from other infectious cattle through the environment. The range of probabilities for *in utero* transmission was based on stage of infection as reported in a meta-analysis (35). Many previously reported models considered the potential for *in utero* transmission as a fixed probability (17–21, 23), with some referencing the same meta-analysis (35) as either the sole (19, 20) or an influential source (24, 37).

The probability of transmission from the dam to the calf for the current model was derived from a calibration optimization exercise as existing data were based on dairy herds where calves typically remain with their dam for hours or days. The resulting probability was consistent with values suggested from experimental and observational studies (36). Beef calves remain with their dams until weaning at 6–7 months providing greater opportunity for dam to calf transmission. This value, referred to in a recent review paper (16, 36) as pseudovertical transmission, was not included in the previous two beef models (7, 17). Dairy ABMs that included this risk typically focused on transmission at the time of birth and transmission through milk or colostrum (19–25, 37).

Indirect transmission of infection was captured as a risk from the global environment and was dependent on the prevalence of infectious animals and a contact parameter. The frequency of infective contact parameter estimated the rate of MAP infection for preweaning calves given contact with MAP from infectious cattle. This parameter was also based on a calibration exercise to estimate a value that would best reflect the longitudinal prevalence data from infected herds in western Canada (56). This estimate of the transmission risk associated with contact with other cattle and the environment is the most context specific of all model parameters and was not one where current literature could provide appropriate estimates. This value was then adjusted by previously reported estimates of the differential susceptibility of postweaning calves and adults (36) to reflect the rate of horizontal transmission in these age groups given contact with MAP from infectious cattle.

The value of the contact parameter will be highly dependent on the intensity of the management system and resulting opportunities for contact among animals and with contaminated environments. Due to the number of management groups, pastures and pens used by a typical beef herd in western Canada throughout the production cycle and the varying density of animals within these areas and associated environmental conditions, there was no attempt to specifically model risk from a single or series of specific environmental reservoirs as has been reported in many of the dairy ABMs (19, 20, 24, 37).

The source of the most analogous transmission parameters in the dairy ABMs varies greatly. The transmission parameters range from assumptions, to values extracted from other observational studies in dairies, to calibrations that are either not specifically described or were reported as manual exercises, to values calibrated in other models and, in one case, to optimization of selected parameters using a random-forest classifier (18, 19, 22–24). One compartmental model (65) included calibration of unknown transmission rate parameters for specific environments by comparing model prevalence over time to published field data corrected for test characteristics. The resulting transmission rates were later cited in a series of ABMs (24, 26, 37). Another leveraged a unique dataset from 102 random farms with no control actions against MAP, generated a 3D parameter space and then visually identified the set of parameters that most closely resulted in a stable prevalence (19).

Although a calibration-based parameter estimation technique does suffer from limitations, we believe that it was a technique well suited for estimating the infective contact parameter and associated probability of infection from dam to calf before weaning for this model. The automated procedure used in this instance offers substantial advantages over manual calibration exercises in its optimization algorithms and capacity to manage stochastics and uncertainty in other model inputs. For the 4,000 iterations testing different parameter values proposed by the optimization function, 25 repetitions were completed for each iteration to capture the impact of model stochastics and distributions of other parameters defined by the literature. While the authors (66–70) and others (24, 71–73) have contributed computational statistics and machine learning techniques that offer greater sophistication and can excel in supporting automated parameter estimation *via* sampling in higher dimensional parameter spaces, the current results were well supported by the optimization-based approach used here.

Most traditional calibration procedures matching to a single data source are best suited to estimating a single parameter. However, published longitudinal MAP data was very difficult to identify for infected beef herds. One unique longitudinal data source was developed in collaboration with industry and used in the actual calibration experiment for the present study. To further validate the calibration outcome, the results of simulations for both the no testing and other limited ELISA-based testing scenarios were compared to two recent cross-sectional studies reporting within herd prevalence for cow-calf herds with evidence of MAP infection but limited or no previous history of testing and culling (34, 58). The range of apparent prevalence reported for positive herds in these studies ranged up to 15–45% and provided strong independent support for the findings of the present study.

While calf to calf transmission has been documented in dairy herds (74), the relative importance of this pathway was discounted in a previous modeling study (65). However, calf-to-calf transmission continues to be incorporated into some ABMs (23, 24, 37). In beef herds, where calves prior to weaning at 6–7 months are typically grazed with large groups of cow-calf pairs, any potential impact of calf-to-calf transmission on MAP control efforts would be indistinguishable from the greater risk of transmission from either the dam or other infected cows or bulls in the management group. Calf-to-calf transmission was not explicitly modeled here as it would not have been feasible to estimate parameters for a third relatively minor contributor to the MAP transmission.

The period during which cattle are susceptible to infection is widely acknowledged as being an important source of uncertainty in modeling (48). Most researchers deemed animals to be resistant to MAP infection after 1 year of age (7, 15, 21, 23, 24, 37, 64) while some, including the present model and one of the beef papers, considered resistance to increase with age or acknowledged the potential for adult infections (22, 25, 75–77). The potential for adult transmission was retained in the current model based on the differences in productive lifespans for beef and dairy cattle. Cows >10 years of age are relatively common in western Canadian cow-calf herds (42), while for dairy herds the productive lifespan has been reported to be < 5 years (78). As such there is more opportunity for beef cows infected after 1 year of age to start to shed later in life regardless of whether they eventually develop clinical signs or not.

Model disease states were commonly split into the following categories: susceptible, latent, subclinical (low shedding), and clinical (high shedding), with the infection pressure stemming from the number of shedding animals in the herd, as in the present model, or density of bacteria in the environment. Some models allowed for transient shedding in groups of calves (24, 37) and others also accounted for factors that have been shown to influence infection and disease onset, such as age at exposure (7, 15) or stressful events including calving or changes in feeding (21). In the current model, a distribution of transition times was used to inform progression of infection to shedding MAP and then apparent clinical disease. Other dairy ABMs reported latent periods consistent with the minimum values used in the present model, and values consistent with the distribution of subclinical duration (24, 37). Shorter latent periods have previously been associated with higher herd prevalence in dairy herds (11). Other dairy models reported rates for these transitions that when converted to time to event were consistent with the values used in this model (18–20, 22, 25).

A variety of different diagnostic tests were used in previous models to identify infected animals. These tests included milk and serum ELISAs, and fecal culture used either separately or in combination. The identified ABMs included a single test sensitivity value for each stage of infection and an overall test specificity value based on previous research (7, 15, 23–25, 37, 64, 75). In the present model, three diagnostic testing options were considered: serum ELISA, fecal PCR and pooled fecal PCR. Each test was described by a pert distribution for sensitivity and specificity for each stage of infection (infected, subclinical and clinical) and an overall distribution specificity value (34). This feature more completely reflects the uncertainty regarding the variation in test performance depending on the stage of infection than using a single value.

Fecal PCR has rarely been reported as a testing option in simulation studies (50). The peer-reviewed data describing the sensitivity of the commercial PCR test protocol are limited, but the reports of PCR sensitivity for the protocol used in regional diagnostic laboratories are lower than the values used here (60, 61). However, when the values reported in the other papers were used in place of the estimates generated for this population and laboratory, the individual PCR continued to perform better than its closest competitor, pooled PCR, in 75% of simulations. Other previously described parameters, most of which were included as robust distributions in the model, were substantially more influential that PCR sensitivity.

The development of simulation models for the evaluation of control practices relies on available data from previous research. However, there are still many gaps in the literature related to the epidemiology and prevalence of Johne's disease, especially in beef cattle. Therefore, assumptions must be made for certain parameters where existing quantitative data is lacking. In the current model, assumptions were made about the minimum age at which animals were likely to become infectious as well as the relative amount of infectious material shed from subclinical as compared to clinical cows in this environment. In examples of the most recently published ABMs some of the most common disease associated parameters influenced by assumptions or expert opinion included persistence of bacteria in the environment and impact of cleaning (24, 37), reduction of exposure due to calf rearing improvements, chance of getting infected from the environment (22), relative MAP shed in different disease states (21), and time in various disease states (19). In previous beef models, Humphry, Stott (17) assumed the bacterial survival rate in the winter months to be 10 times higher than the bacterial survival rate in the summer. Model construction provides an opportunity to identify areas where current knowledge is lacking as a focus for future research.

The objective of the present model was to identify effective strategies with the lowest direct costs of testing for reducing the within-herd MAP prevalence in beef herds. Bennett, McClement (7) describe a model designed to determine the effects of testing, culling and improved management practices on Johne's disease in UK beef herds, as well as the associated costs of implementing control measures. Bennett, McClement (5) later examined the impact of improving the sensitivity of the ELISA test studied, but did not explore other strategies. A dairy ABM examining the role of testing and culling with serum ELISA found increasing testing frequency from every 2 years to every year was associated with increasing success (24). One compartment model reported that with twice yearly testing with serum ELISA, the probability of fadeout within 25 years increased (64). Another model using serum ELISA and fecal culture in series, examined the impact of testing and culling based on parity (23), but did not find an overall economic benefit. Other ABMs examined the impact of milk ELISA which is not an option for extensively managed beef herds (19, 21, 25). The current beef model is unique in the combination of test types compared as well as the frequencies of testing and strategies for adopting risk-based testing.

The choice of testing strategy for the control of Johne's disease is highly dependent on a producer's motivation and goals for disease control (79). The results from the current model show that after a 10-year period, 7 testing scenarios reduced the within-herd disease prevalence such that 75% of simulations were below the starting prevalence of 5%. Whole herd testing with individual PCR at a frequency of either 6 or 12 months, and whole herd testing with pooled PCR at a frequency of 6 months were the scenarios that resulted in a median 10-year prevalence of 1% or lower. Additionally, restricted testing of cows with fewer than 4 negative tests with individual PCR every 12 months, whole herd testing with individual PCR every 24 months, and whole herd testing with ELISA every 6 months resulted in a 10-year prevalence below 2%. By contrast, the within-herd prevalence of Johne's disease increased from 5% to a median >3 times the original prevalence over a 10-year period when no testing or culling was performed.

Overall, individual PCR was the most effective diagnostic test across all scenarios for reducing disease prevalence, followed by pooled PCR and then ELISA. This finding was robust to inclusion of a broader range of diagnostic test sensitivities as reported in the literature. Furthermore, increased testing frequency was shown to have a positive impact on reducing disease prevalence over a 10-year time period. Finally, whole herd testing scenarios were more effective compared to risk-based testing scenarios. If the focus of the test and cull strategy is to substantially reduce within-herd disease prevalence regardless of the cost, then the results of this study suggest that testing the whole herd using individual PCR at an increased frequency of 6 months is the most effective option based on the much higher frequency of instances where the prevalence was 0% in year 10.

Scenarios that were both most likely to be effective at reducing disease prevalence from the initial subclinical prevalence of 5% over 10 years and were the least costly included testing the herd with individual PCR every 24 months, pooled PCR on the whole herd every 12 months, and testing cows with fewer than 4 negative tests every 12 months using individual PCR. These scenarios could be considered where the goal is to lower the within-herd prevalence of disease while also factoring in the cost of testing. However, of these options, testing cows with fewer than 4 negative tests every 12 months using individual PCR rank most favorably with respect to the predicted prevalence 10 years after the start of the testing program. The decision to consider cows with at least 3 previous consecutive negative tests as “test-negative” was suggested by a 20-year longitudinal study in a Pennsylvania dairy herd (57).

Sensitivity analysis suggested that testing remained effective at reducing disease prevalence compared to no testing after 10 years with varying initial prevalence values, expected prevalence in purchased cattle and calibrated values for transmission risks. Furthermore, the relative performance of the diagnostic tests remained the same, with individual PCR resulting in the lowest prevalence over time followed by pooled PCR and then ELISA.

Previous models designed for beef herds recommended a combination of testing and culling and management improvements to reduce transmission (5, 7). Several dairy models have also highlighted the importance of management changes as the preferred strategy to testing and culling or as an important component in addition to testing and culling (21). Caution is necessary when considering these results, because as previously noted the assessment of the impact of management changes was typically based on more assumptions in these models than the assessment of testing and culling strategies. Camanes, Joly (24) reported that test frequency and culling a proportion of moderately positive animals were the two most influential test and cull parameters for long term control efforts. Reducing calf exposure to possible MAP infection sources was also highlighted as an effective strategy (24). The present study also found a positive impact on disease prevalence with increased testing frequency. The sensitivity analysis in the present study also suggests a substantial impact of disease transmission parameters and the potential benefits of management interventions, if practical options can be identified to reduce transmission risk, especially for young calves.

The current study provides the direct cost of testing on a yearly basis for each scenario and identifies testing options that are both effective and least cost for situations where testing has been deemed appropriate. Testing a herd for Johne's disease requires considerable financial investment (7, 62) and is a frequently highlighted as a barrier to testing and culling (5, 7, 80). However, there are substantial economic losses associated with Johne's disease for infected beef herds due to reduced productivity, early culling, replacement costs, comorbidities and veterinary expenses (4–6).

A producer's decision to test for Johne's disease is dependent upon a number of factors, including initial within-herd disease prevalence, overall goals of the specific operation and negative economic consequences associated with herd infection (79, 81, 82). Seedstock producers would likely be more inclined to test for Johne's disease, regardless of the cost, as their business and reputation is dependent upon the health status of their herd (82). Commercial operations are more concerned with the economics related to the sale of calves at weaning, such as calf weaning weights, and therefore have less incentive to test if disease prevalence is sufficiently low that productivity and profitability are not substantially affected (6). A more in-depth economic analysis comparing the cost of each test and cull scenario to the cost of the disease within the herd is needed to determine if testing and culling is a financially attractive option on commercial cow-calf operations.

One of the outcomes of this study was to rank the relative effectiveness of testing strategies under management conditions typical for an extensively managed commercial herd in western Canada. As is the case with any model, simplifications and generalizations within the model design affect its ability to truly replicate real-world situations. This model is therefore meant to act as an estimation tool and guide for the relative evaluation of Johne's disease control options rather than to offer precise predictions for specific herd scenarios. As noted in the introduction of one of the first models to describe MAP in beef herds, the creation of a model forces an exploration and explicit description of the disease processes and outcomes (17). This description of what we know and where we are still uncertain can be of value in managing the disease and also targeting future research questions even when the resulting predictions are limited by the underlying data.

## Conclusion

The overall goal of this model is to assist veterinarians and producers in making complex Johne's disease testing and control decisions that are supported by current scientific evidence. Decisions regarding whether to test, which tests to use, how many animals to test and how often are complicated by the imperfect nature of the tests, variation across previous research and the extended time course of the disease. Moreover, decisions related to testing are also highly dependent on a producer's motivation to control Johne's disease in their herd with operation-specific goals and economic impact being the main driving factors. The present model accounts for imperfect diagnostic test performance when comparing testing scenarios and quantifies some of the uncertainty due to chance in model predictions. Furthermore, it considers the long and variable period from exposure to disease when comparing intervention effects on disease rates, and uncertainty associated with age susceptibility and transmission risks. The input values in the publicly available version of the model can be customized by other users to reflect the characteristics, management practices and initial infection status of individual cow-calf herds to better inform testing options that are best suited for a specific herd.

## Data availability statement

The original contributions presented in the study are included in the article/Supplementary material, further inquiries can be directed to the corresponding author.

## Author contributions

PJ, CW, and LM wrote the initial draft of the manuscript and performed statistical analysis. CW and YQ developed the ABM. CW, PJ, and LR developed the testing scenarios that were analyzed using the model. CW, LM, JC, KL, LR, and NO edited the manuscript and provided feedback and revisions. All authors contributed to the article and approved the submitted version.

## Funding

This work was supported by the Saskatchewan Ministry of Agriculture (20180128), Beef Cattle Research Council (ANH.11.18), Natural Sciences and Engineering Research Council (548206-18), and with joint funding from Alberta Beef Producers and Saskatchewan Cattlemen's Association (Project ANH11-18).

## Conflict of interest

Author LR is the owner of the company Rosengren Epidemiology Consulting. The remaining authors declare that the research was conducted in the absence of any commercial or financial relationships that could be construed as a potential conflict of interest.

## Publisher's note

All claims expressed in this article are solely those of the authors and do not necessarily represent those of their affiliated organizations, or those of the publisher, the editors and the reviewers. Any product that may be evaluated in this article, or claim that may be made by its manufacturer, is not guaranteed or endorsed by the publisher.

## Abbreviations

MAP: Mycobacterium avium subspecies paratuberculosis
ELISA: Enzyme-linked immunosorbent assay
PCR: Polymerase chain reaction
BCS: Body condition score.
