# Supplementary material for: Investigating effective testing strategies for the control of Johne's disease in western Canadian cow-calf herds using an agent-based simulation model
Source: Front Vet Sci. 2022 Nov 25;9:1003143. doi: 10.3389/fvets.2022.1003143 (PMC9732103; doi:10.3389/fvets.2022.1003143)
Supplement: Supplementary file 1 [file Data_Sheet_1.pdf]

Investigating effective testing strategies for the control of Johne's disease in western Canadian cow-calf herds using an agent-based simulation model

Model description supplement

Paisley Johnson, Lianne McLeod, Yang Qin, Nathaniel Osgood, Leigh Rosengren, John Campbell, Kathy Larson, Cheryl Waldner

An Interactive Tool to Inform Johne's Disease Control in Beef Herds

The model description follows the ODD (Overview, Design concepts, Details) protocol for describing individual- and agent-based models (1), as updated by Grimm, Railsback (2).

1 Purpose and Patterns

The objective of this project was to develop an agent-based model (ABM) to compare the relative effectiveness of various diagnostic testing scenarios for the control of Johne's disease in western Canadian cow-calf herds as measured by infection prevalence, clinical disease occurrence, resulting culling and replacement numbers, herd productivity (calves weaned and weaning weights) and testing numbers to determine testing costs. Johne's disease is a contagious, chronic and incurable disease of ruminants caused by *Mycobacterium avium* subspecies paratuberculosis (MAP).

The base model represents a simplified production cycle within a cow-calf herd and the outputs emulate the patterns we would expect for typical numbers of animals of different production stages and ages, pregnancy outcomes, calf survival and weaning weights from western Canadian beef herds (3, 4). The model of Johne's disease in the absence of testing represents the expected patterns of the time course of infection prevalence based on data shared by herds enrolled in a long-term surveillance and control program from western Canada (https://skstockgrowers.com/Johne's-disease-surveillance-program/).

2 Entities, state variables, and scales

The primary entities in the model are cows organized into a single herd. There is also an agent representing the testing process and a main agent containing the environment in which the cattle are located. The only collectives in the model are tracked listings of calves born to individual cows. Cows are directly connected to their calf. The environment consists of a series of three holding areas: a summer pasture used for breeding, a winter holding area where the cows calve, and a pen where the bulls are held outside the breeding season.

The state variables that differentiate among the cow agents include sex, age as continuous measure, weight in pounds, and body condition score (BCS) on a 5-point scale. Other characteristics of the agents are captured in specific state charts (detailed in sections 3 and 5) describing their production group, stage of reproductive management, location, stage of infection with MAP, and testing status.

The model is built with time on a continuous scale with a month as the primary unit of time. The model runs over a period of 10 years. Space is not explicitly described other than for locating cows and bulls within specific holding pens. The model ignores spatial and location-based interactions between cows as it does not differentiate between infection resulting from contact with contaminated feces in the environment as compared to direct contact with feces from infected cows.

3 Process Overview and Scheduling

An ABM was developed to provide a customizable template for studying Johne's disease progression and testing for control in a commercial cow-calf herd in western Canada. The model creates a spring calving cow-calf herd that is followed through a series of production cycles over a ten-year period. A spring calving herd was chosen for two reasons. First, based on a recent survey herds contributing to this project, March was the most common month to start calving (5). Second, herds that calve in the winter and early spring are potentially most at risk for infectious disease transmission due to the relatively higher intensity of management required to calve in very cold temperatures in western Canada. The model is structured to maintain a dynamic but stable population of calving cows where the numbers of females fluctuated throughout the production cycle as would be expected in a typical herd undergoing culling and replacement. Females exited the herd through culling due to failure to become pregnant, old age at 12 years or Johne's disease (clinical disease or positive test results), and calves were sold post-weaning, with the exception of retained replacement heifers. Similarly, a dynamic population of breeding bulls was culled for age at 6 years or Johne's status and replaced through purchase of yearling animals.

Cow agent

The **cow agent** is the core component of the ABM and includes individual-level characteristics such as parameters, state variables, functions, actions and rules to trigger the actions (captured in state charts). There are five sets of interacting state charts featuring: 1) production stage classification and sex with incoming purchased animals, sales of weaned calves and bull management; 2) the cow-calf production cycle including exposure to bulls, pregnancy testing, female culling and calving; 3) cattle movements between pens and summer pasture; 4) MAP infection and development of clinical disease and culling; and 5) blood and fecal testing (pooled and individual) and associated culling. In addition to state charts, weight and BCS dynamics are encapsulated as a hierarchical agent inside each cow agent, capturing continuous weight change of calves, heifers and cows and categorical changes in BCS.

Stage of production state chart (Biological processes)

The production stage state chart, as shown in Figure S1, describes the classification of animals, including preweaning or nursing calves, weaned heifers and steers, postweaning replacement heifers, bulls, breeding females, and purchased cows or heifers. To describe the cow agent in further detail, the 'breeding females' state is subdivided into replacement heifers, bred heifers (exposed to bulls), pregnancy tested heifers, and cows. Branches inside

weaned heifers locate the events related to the decision to retain heifers after weaning. This decision is timed to occur after testing the bred heifers and cows for pregnancy status to best inform necessary replacement numbers. After all culling was completed and the number of retained pregnant females was determined, pregnant cows were purchased if necessary to maintain the number of calving females.

Agents move between states based on a specified timeout that responds to conditions and message transitions. At model initialization, given specified distributions of age and gender, the agents are divided into four categories: bulls, postweaning calf, pregnancy tested heifers and cows. At model start up on April 1st, female agents between 2 to 12 years, between 1 year to 2 years, and between 7 months to 1 year enter cows (pregnant), pregnancy tested heifers (pregnant), and replacement heifers' states, respectively. Male agents at model start (between 2 and 6 years of age) enter the bull state. Calves are born into the model on April 1st and transit into the preweaning calf state and all females > 2 years of age transition to the cow state. Females retained in the herd transition from preweaning calf to weaned heifer (to 7 months) to postweaning calf (12 months), replacement heifers (13 to 15 months), bred heifers (16 to 19 months), pregnancy tested heifers (20 to 24 months) and finally cows (>24 months), as the animal ages. The purchased cow and heifers state is used to receive purchased cows and heifers. For simplicity, if heifers are purchased as replacements, only pregnant heifers are purchased shortly after pregnancy testing.

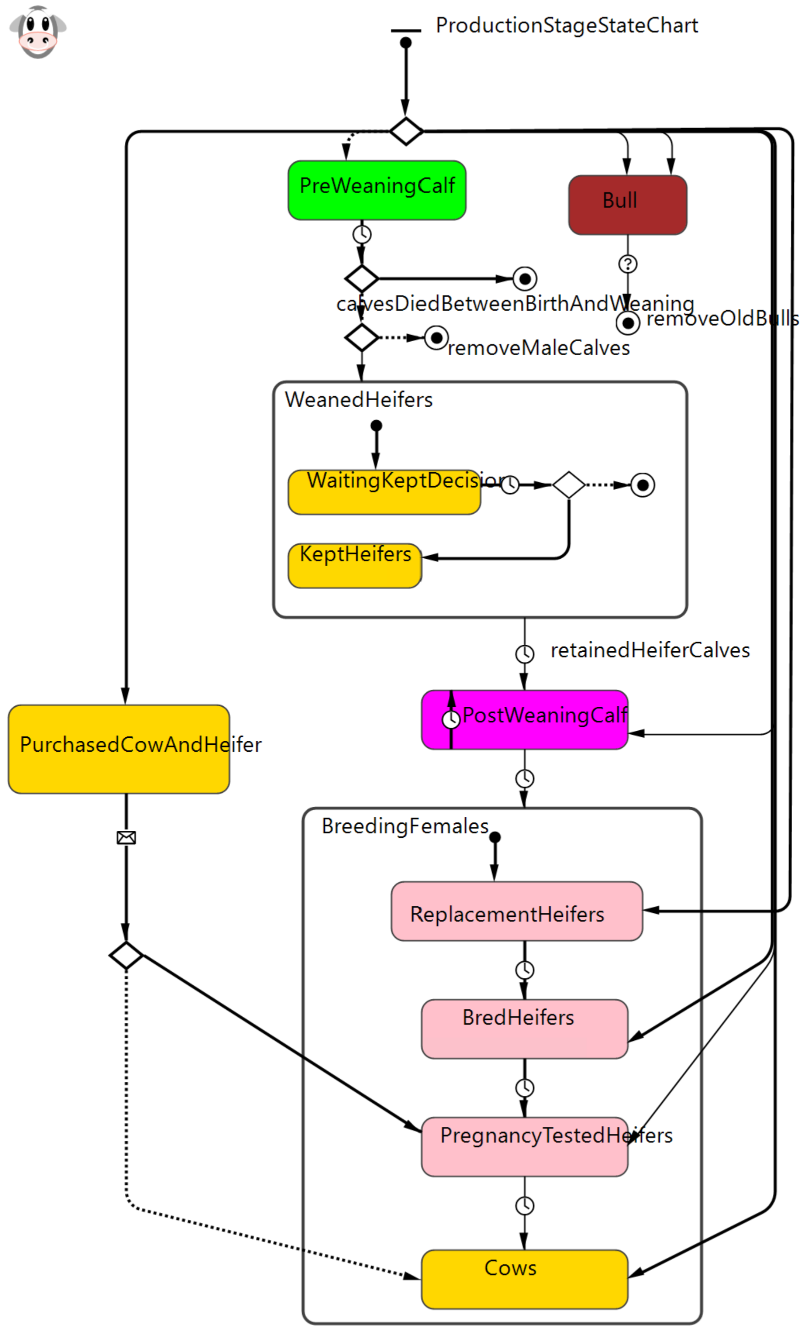

Figure S1. Production stage state chart

Production cycle state chart (Farming-related processes)

The cow-calf production cycle is represented using a state chart which governs how heifers and cows move through each stage of the breeding, pregnancy and calving cycle (Figure S2). All cows and pregnant heifers calve on April 1st each year. Having all of the cows calve on the same day is one of the primary simplifying assumptions of the current model. Having all animals age at the same time makes it easier to maintain a stable population over time and failure to have a distributed calving season does not impact the testing options for Johne's control tested in the model.

Cows are exposed to bulls 3 months after calving for 3 months. One month after the bulls are removed, the cows are pregnancy tested. Five months after the cows are pregnancy tested, they calve after a total gestation of 9 months. The probability that a cow is pregnant at pregnancy testing varies based on whether the animal is a heifer or cow (4) and also varies based on her BCS as informed by existing field data (3, 6) and her MAP status.

After pregnancy testing, cows are removed from the herd for old age (>12 years), if they are not pregnant, if they have clinical Johne's disease, and whether the cow is the daughter of a Johne's positive cow, if that management option is activated in the model. The decision to cull cows >12 years was based on data from previous research on the age distribution of cow herds in western Canada (7) and the need to simplify maintenance of a stable herd in which to run experiments related to management of Johne's disease.

When a cow gives birth in the calved state, there is a birth event where a calf is introduced into the model. Within the birth event information is passed to the calf including the mother's age, whether the dam was purchased, and the dam's infection status. Other information recorded for the calf at birth includes calf sex and weight at birth. The calf's subsequent rate of gain (see subsequent sections) and probability of survival to weaning varies based on whether the dam is a heifer or cow and the calf's sex (3, 4, 6, 8) and whether the cow was purchased or born on farm (default is no difference). The cow's information is also updated with the identification of the calf and the total number of calves for the cow. This allows for the identification and the option of culling of daughters from positive cows.

Purchased pregnant cows or heifers directly enter the purchased pregnant state as they join the herd. Subsequently, the purchased cows and heifers will transition to the calved state, and then follow the rest of the cows in the herd.

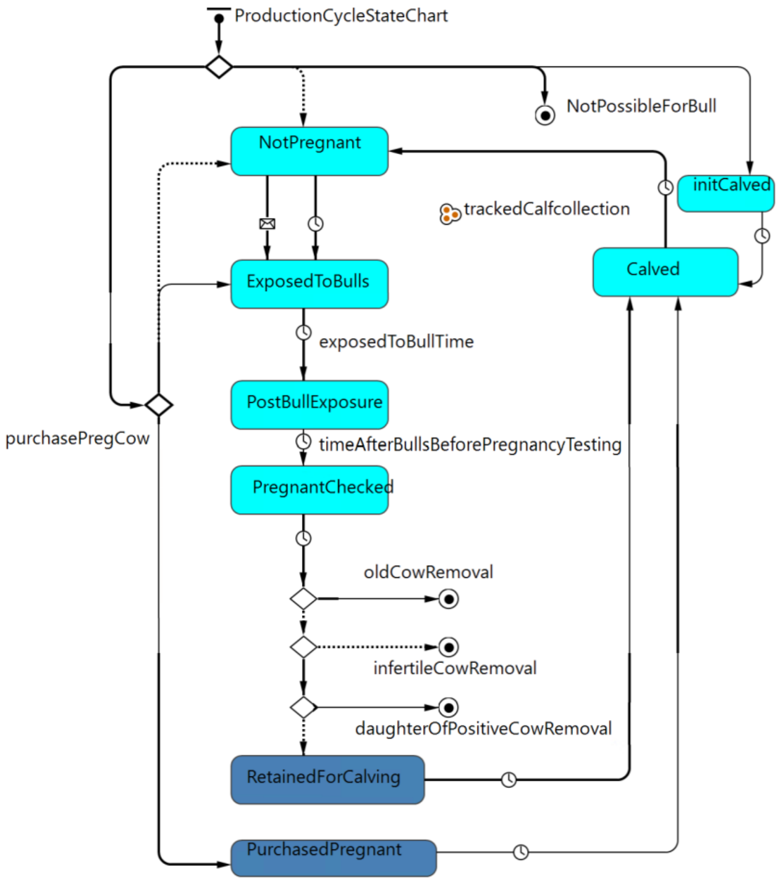

Figure S2. Production cycle state chart

Johne's disease state chart (Pathogen-related processes)

The Johne's disease classification representation is shown in Figure S3, describing the stages of infection with MAP and progression to clinical disease as well as MAP transmission from cow to calf (in utero or via fecal transmission from birth to weaning) and other fecal transmission of MAP either directly from animal to animal or through the environment. Disease states included susceptible, latent infection (silent, infected but not infectious), and infective. Infective animals included subclinical MAP infection (moderate shedding) and clinical disease (high shedding, with apparent clinical signs of diarrhea and weight loss). The model assumes for simplicity that Johne's positive cows are culled before they die given an active, voluntary control program.

All animals are assigned to this state chart at the start of the model based on initial prevalence of infection parameters in the model. This value was based on the prevalence of MAP in infected herds participating in the Canadian cow-calf surveillance network (C3SN) (5, 9). The initial prevalence was chosen to reflect MAP-infected herds at the time the herd owner would be likely to observe the first clinical cases, recognize the problem and test. The chosen value was the minimum observed within herd prevalence from positive herds. It was also consistent with the first whole herd test results from the 10 positive herds that provided calibration data

for this analysis. Existing testing data were applied to the infectious subclinical stage of the model. The initial prevalence value for the latent stage was assumed to be the same.

Purchased cattle are also assigned based on expected prevalence in randomly purchased animals. Initial prevalence estimates were based on a recent observational study reporting BLCM estimates of true prevalence for Canadian cow-calf herds (5, 9). Purchased animals were assumed to be a random animal from the Canadian cow-calf population and not specifically from an infected herd (5, 9). Sensitivity analyses were also undertaken to explore the impact of purchasing animals from herds with moderate or high MAP prevalence (5, 9).

Infection of susceptible animals occurs directly based on direct cow to calf transmission *in utero* or through fecal contamination from infected dams from birth to weaning and among other animals through direct fecal transmission or indirectly through the environment (10-12).

Calves born to infective cows have a probability drawn from a pert distribution for each simulation of contracting MAP vertically *in utero* through their dams depending on if the dam was in the subclinical or clinical stage of disease (11). Calves could also be infected through fecal contamination based on a probability before weaning depending on whether the dam was clinical or subclinical (13, 14). The probability for transmission from the dam to her calf from birth to weaning was estimated through calibration procedures described in subsequent sections.

The description of all other fecal transmission was an intentional simplification. As the infection is primarily transmitted through feces, a choice was made to not distinguish between animal-to-animal fecal oral transmission and transmission through fecal contamination of the environment due to the extensive management of most herds throughout the year and difficulty in meaningfully modeling risks of transmission in specific environments as reported by others for more intensively managed herds (15, 16). Susceptibility to infection varied based on animal age. Calves less than 6 months of age were most susceptible, calves between 6 months and 1 year were considered to be about half as likely to be infected given exposure and calves older than 1 year were substantially less likely to be infected given exposure (13, 14). The age-related coefficients used to adjust the probability of infection given contact are drawn from pert distribution as described in Table 1, and for adults sequentially decrease by an inverse function based on age in years. For calves infected through the fecal infection pathway, but where their dam was also infective, the route of transmission is recorded as having been due to infection from the dam.

The rate of infection per year is a dynamic value based on the actual current prevalence of infective animals as well as a calibrated parameter representing the difficult to differentiate combination of probability of infection given contact between a susceptible individual and MAP shed by infectious cattle and a contact rate (frequency of infective contact) (details in the Calibration section). The base value of this calibrated parameter reflects the potential for horizontal transmission from MAP shed by infectious adults to preweaning calves. The rate is

then age-adjusted for postweaning calves and for adults as described previously. The weighted prevalence of subclinical and clinical animals provides a measure of the current risk of exposure to MAP. Subclinical animals or moderate shedders are assumed to contribute to the weighted prevalence 20% as much as clinical animals to reflect values similar to those reported in other models.

A set of transitions associated with rates were employed to govern progression from infected to clinical disease stages based on limited data on the expected durations of each stage from the existing literature (17-20). The mean duration associated with a timeout was drawn from a pert distribution informed by mode, minimum and maximum values reflecting both limitations in the available literature and the biological variability of the course of infection among different animals. The rate of transition to the next state for each event within a run was derived from an exponential distribution of 1/mean of the selected duration for each simulation.

All cows with clinical Johne's disease, exemplified by progressive diarrhea and severe weight loss, are removed either within a specified period after clinical signs began or at pregnancy testing, depending on which is sooner. The period from clinical signs to removal is governed by a pert distribution to account for the variability in identifying cows with clinical signs and removal from the herd (Table 1). The values were chosen to represent the range of values likely with a voluntary control program where cows would be culled after testing and before calving.

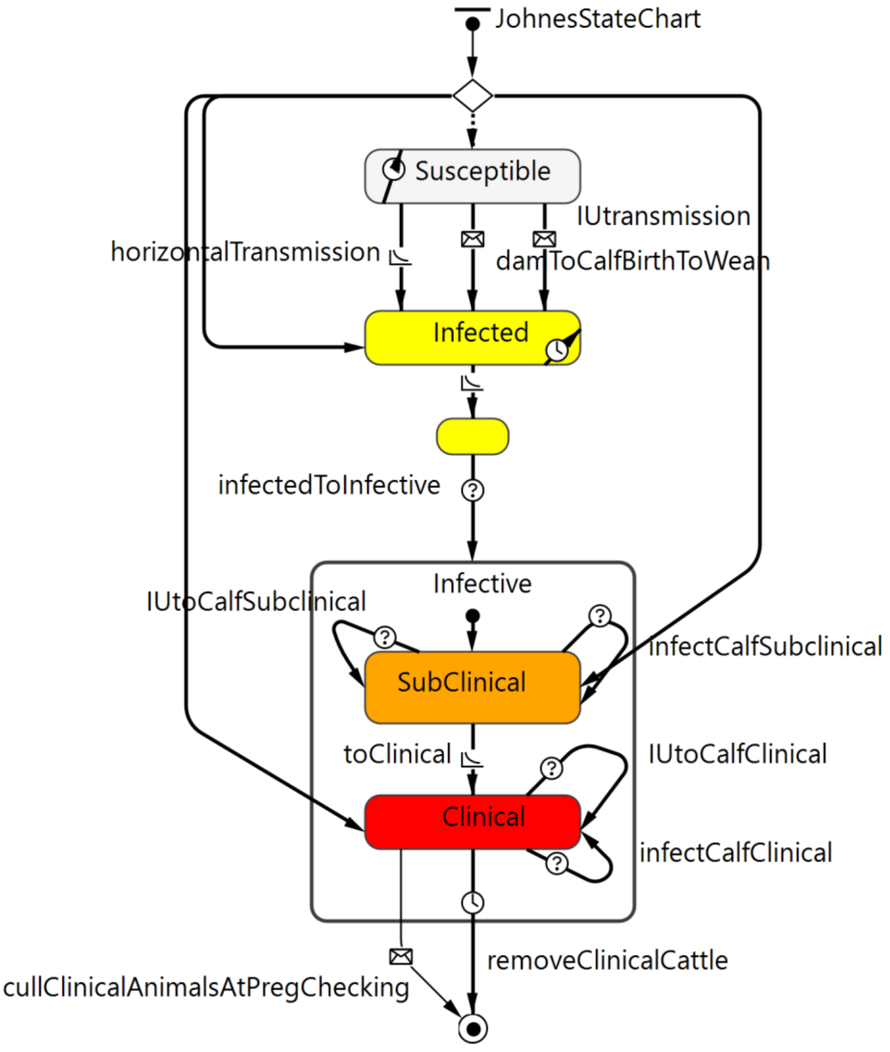

Figure S3. Johne's disease state chart

Fecal and Blood Testing State charts (Farming-related processes)

The fecal testing state chart and blood testing state chart describe the process of individual sample fecal and blood testing for Johne's disease, as shown in Figure S4. Based on test results, an agent is determined either to remain in or to be removed from the herd. The details of testing timing, frequency and duration are governed by a test control component in the Test class through message transitions.

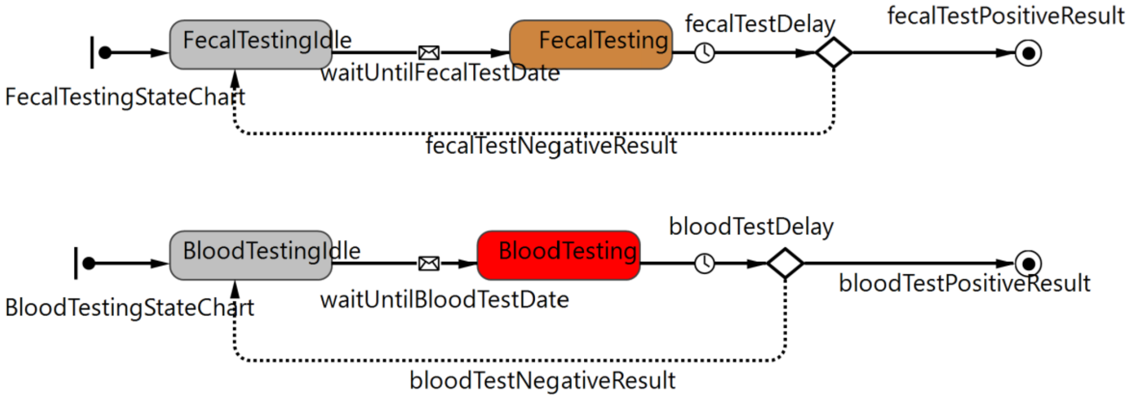

Figure S4. Fecal and blood test state charts

Pool-based fecal testing state chart (Farming-related processes)

The model also allows for testing of pooled fecal samples to save money on testing costs in herds where the expected prevalence of MAP infection is low through a third testing state chart (Figure S5). This state chart in cow also functions based on messages sent from state charts in the testing agent where the pools are established and messages are sent regarding the timing, frequency, and duration of testing. The size of pool can be set through a parameter. However, most labs work with a pool size of 5 samples.

In the first section of the state chart, a pool of fecal samples from eligible animals is sent for testing. Once the result of the pooled test is available, all the samples in the pool are directed to be individually retested through the individual fecal test mechanism if the pool is positive to identify the positive animals for culling. If the pool is negative all animals in the pool are considered to be negative.

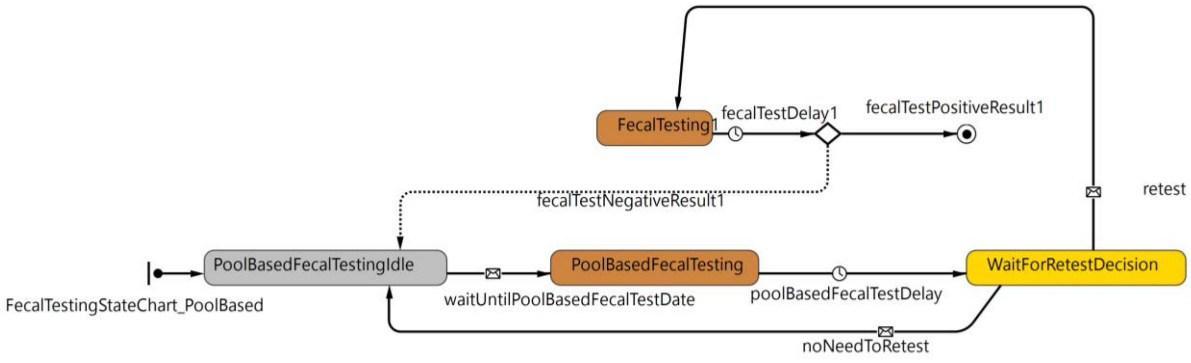

Figure S5. Pool based fecal test state chart

Fecal and blood test action charts (Farming-related processes)

The action charts visually show functions for applying the diagnostic tests and determine the test results of fecal (Figure S6) and blood (Figure S7) tests based on probabilities determined by the sensitivity and specificity of the disease at each stage and the true disease status of each animal.

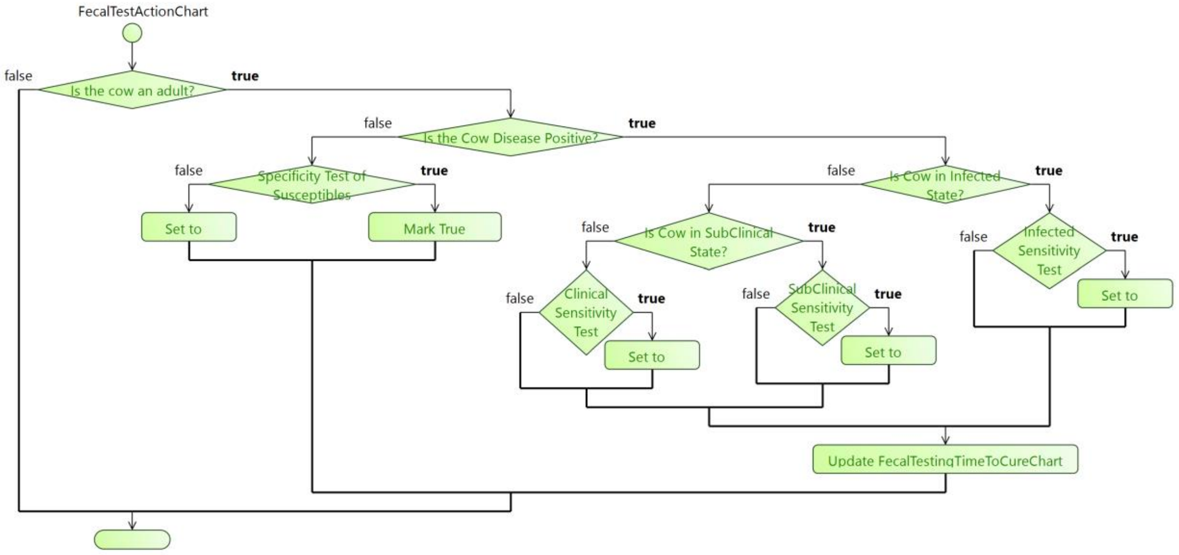

Figure S6. Fecal test action chart

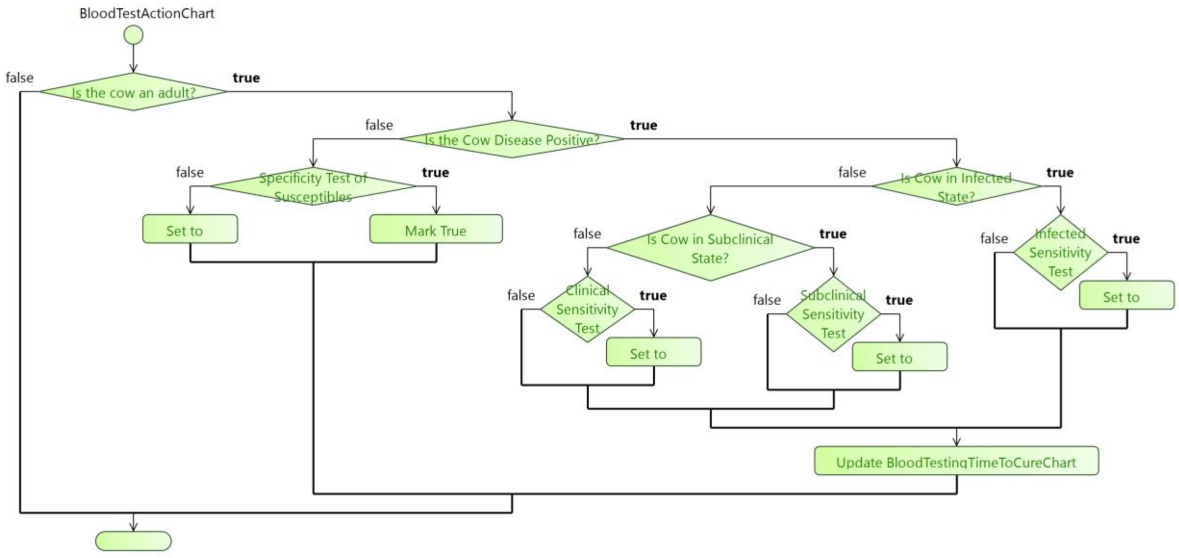

Figure S7. Blood test action chart

Cow movement state chart (Farming-related processes)

A state chart was also used to control movement of animals between summer pasture, female pen, and bull pen based on events corresponding to herd management (Figure S8). The movements for the herd are visually displayed using animation with specific locations as attractants in Main class. Cows and bulls are moved to summer pasture together 3 months after calving. The bulls are with the cows for 3 months and then returned to their home pen. The cows remain on summer pasture until pregnancy testing and weaning.

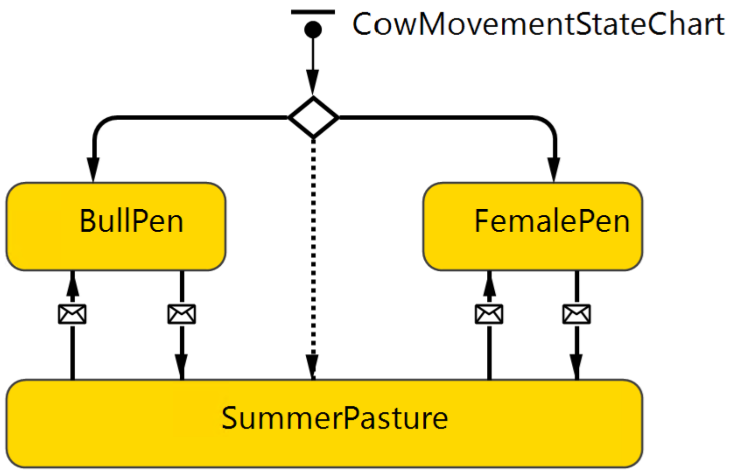

Figure S8. Cow movement state chart

Weight and BCS agent (Biological processes)

The model incorporates the underlying dynamics of weight and BCS as an encapsulated agent inside the cow agents. The weight and BCS dynamics refer to an endogenous dynamic mechanism, representing the evolution in factors related to the weight, such as age, sex, dam history, and Johne's disease status, and BCS, such as stage of the production cycle and Johne's disease status.

The model employs a daily event to simulate average daily weight gain and weight as a continuous variable for both female and male calves. Birth weight is drawn from a distribution specific for whether the calf is from a heifer or a cow. Weight dynamics for an individual calf depends on its characteristics including its sex, dam's age, and dam's Johne's disease status and updated with any change in the dam's status (detailed below). Individual predicted weaning weights informed average daily gain. The target 205 day weaning weight for steer calves from mature, healthy cows was 600 lbs (SD 20 lbs).

Heifer calves retained in the herd continued to gain weight until they reached a distribution of target weights at first bull exposure (65% of mature weight) and then target weight at the time of their first calf (85% of mature weight). The model was simplified such that cows did not gain weight due to growth after their second calf. Mature cow target weight was 1400 lbs (SD 60 lbs). Mature bull weight was not tracked in the model.

The weight of heifers and cows also depends directly on changes in BCS. There is a change of body weight for each change in BCS specified for cows and for heifers. Body weight is tracked in cows and heifers as it relates to the cull value. BCS independently impacts both pregnancy success and cull value. BCS varies by time of year based on annual variation related to the reproductive cycle. BCS and weight decrease from calving to exposure to bulls and then BCS also increases between bull exposure and pregnancy testing. BCS also decreases when a cow transitions to having clinical Johne's disease. Johne's associated changes in BCS independently impacted both pregnancy success (Table 1) and final cow cull weights.

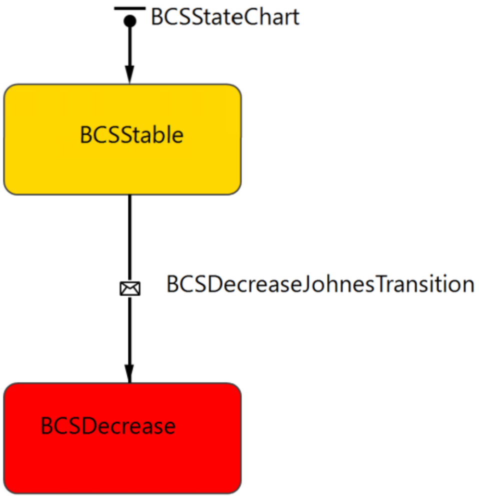

Figure S9. BCS decrease for Johne's disease state chart

Test agent (Farming-related processes)

Testing strategies including options for testing the all animals at a specified frequency (or frequencies), testing for high-risk groups, testing for low-risk groups, and pool-based fecal testing, are modeled using an agent-based model, as shown in Figure S10 to Figure S12. The differences between the testing strategies are the eligible animals and the process for determining frequency. All testing strategies can reset testing frequency after a specified period for a second round of testing. To simplify the publicly available version of the model, only the single risk group frequency option (user specified fixed interval between tests) and pooled-based fecal testing options are visible.

The single risk group testing option and user-controlled high/low risk testing share common steps of the testing mechanism, including waiting to begin testing, specifying criteria to select animals for testing, sending a message to start testing, and waiting for the next round of testing. The agent enters the state chart at beginning of the simulation and waits for testing to start. Following the timeout transition for the start of testing, the agent moves to a state for selecting eligible animals based on several criteria, then sends messages to start testing.

The default setting for the single risk group testing selects all eligible animals in the herd using a minimum and maximum age and maximum BCS for cows. All bulls are tested by default but can also be added as target group to the risk-based testing options. Other options allow further selecting a subset of cows for testing to reduce testing cost and effort. Risk based sampling selects animals at potentially higher risk including whether a cow is purchased or whether a cow is daughter of a positive dam plus or minus cows within a specific range of ages or BCS. Cows can also be selected for testing depending on whether a cow has less than certain number of negative tests based on the idea that a cow that has tested negative more than a certain number of times is unlikely to be truly infected. Finally, a

random subset cows defined by a chosen percentage of eligible cows can be selected for testing.

The main differences between single risk group testing (default) option and user-controlled high/low risk testing are the eligibility criteria for selecting animals to test allowing further customization of the frequency and duration of testing. There is a fixed testing frequency that can be changed once throughout a simulation. For both user-controlled high-risk testing and low risk testing, the testing frequency is fixed for each; however, the two testing strategies are applied with different criteria to select eligible animals. As an example, a user could choose to test all high risk animals and only a random subset of low risk animals. Similarly, they could test high risk animals every year, and low risk animals every two years.

In pool based fecal testing, the testing agents enter a state to create sampling pools from cattle that are eligible for testing based on existing selection machinery within the static testing state chart. Then, from the eligible animals, the model creates random pools of 5 cows. After the creation of the pools and determination of whether there are any infective cows within the pool, the pools are tested using the pooled fecal test state chart in the cow agent using a pre-defined sensitivity and specificity for pooled samples. After a timeout transition waiting for the initial test results, positive samples are retested with the individual animal fecal test. For the pools having negative results, individual samples in the pools report animal status as negative.

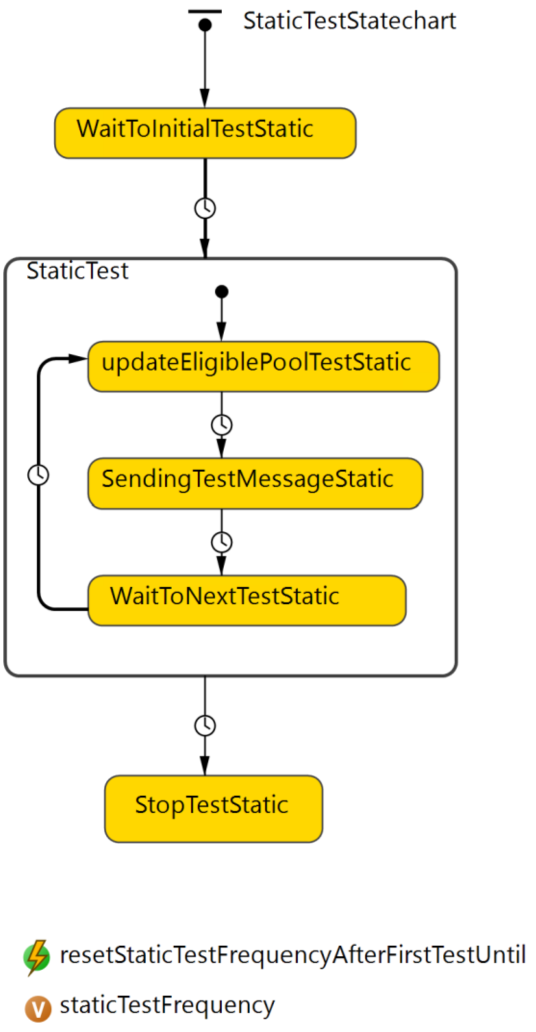

Figure S10 Default (single risk group) testing state charts

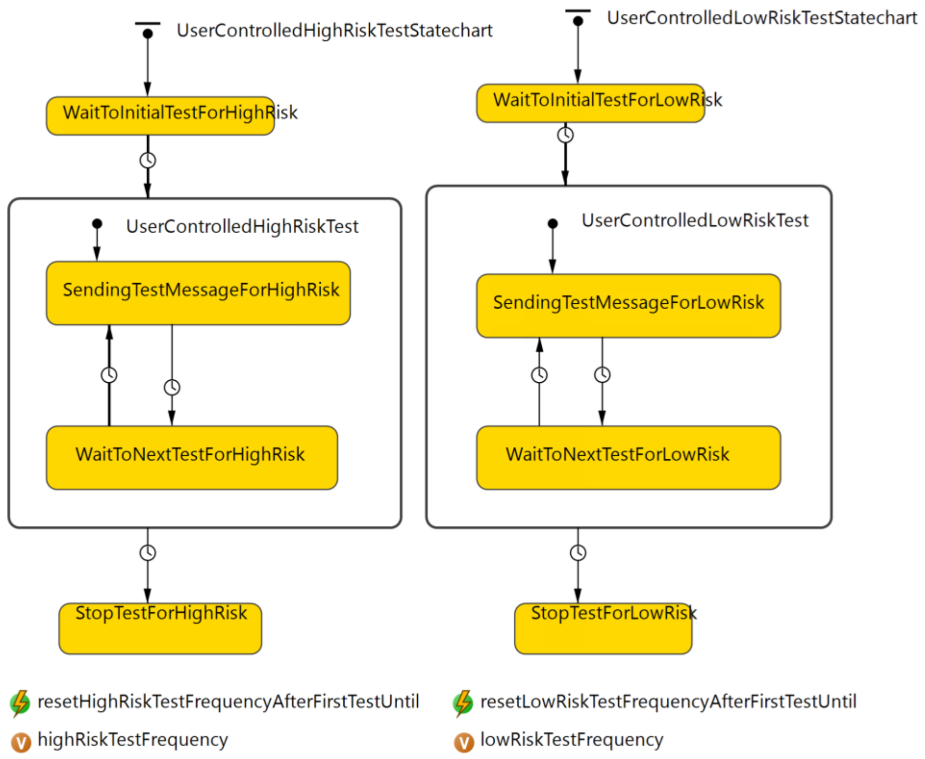

Figure S11. User controlled test for high-risk group and low-risk group state charts

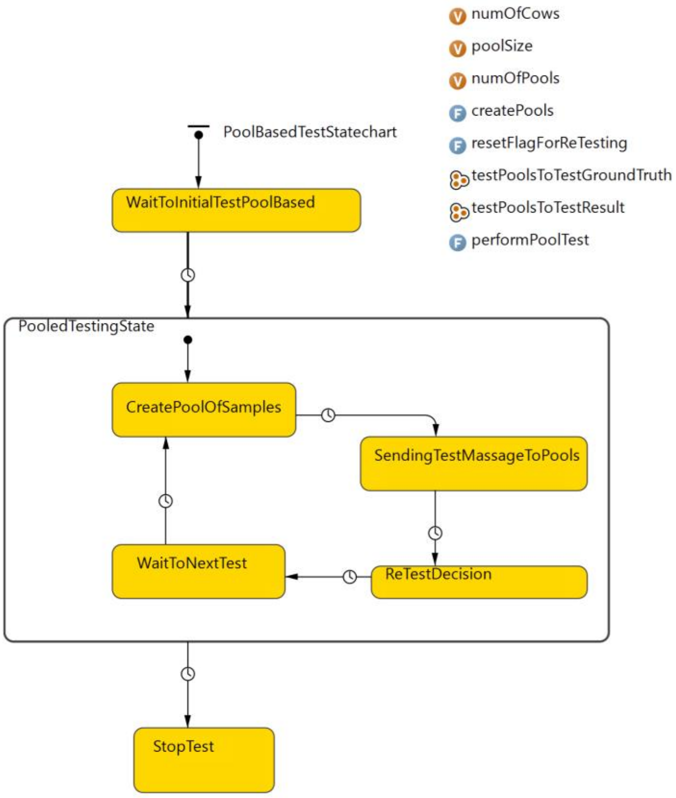

Figure S12. Pool based fecal testing state chart

Main

Model parameters are placed in Main class, as well as a visualization of cow location and movement.

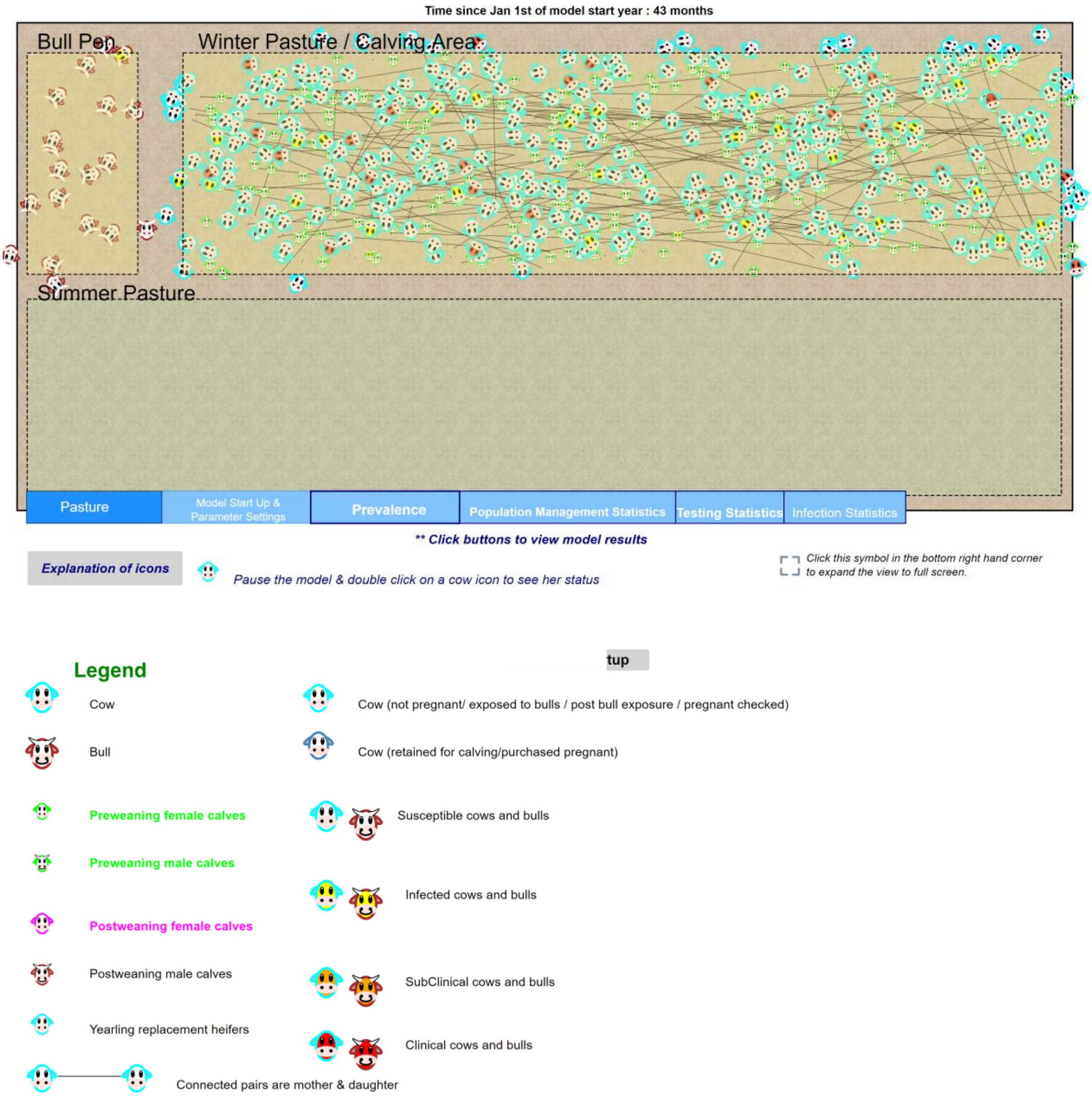

Figure S13. Illustration of cow and bull status and movement during the production cycle

Events and functions to maintain or increase herd size (Farming-related processes)

The default model status is to automatically maintain a consistent number of cows at calving. To automatically maintain the herd size, events and functions located in main class replace all cows culled or lost for any reason, such as cows culled due to old age, nonpregnancy, and Johne’s disease (either by clinical signs or testing). There is also an option to increase herd size once in a specified year to a new target herd size.

There are two sources for cow replacements: home raised heifers or purchased cows and heifers. The model provides options to choose the percentage of total home raised heifers that are potentially available to be retained for replacements at weaning and the relative percentage of pregnant cows or pregnant heifers to be purchased to meet herd size goals.

Each year immediately following culling, the number of pregnant cows, pregnant heifers and replacement heifers to be retained for calving is compared to the desired number for that year. The deficit is determined and filled first by home raised replacement heifers if that option is permitted (% of total heifer calves retained > 0) and to the maximum proportion of all heifer calves to be kept as replacements accounting for the expected percentage that will not be pregnant after their first breeding season. If there is still a deficit in the number of pregnant females retained for calving, then the remainder is filled by purchased pregnant cows or heifers. If there are too many pregnant animals available after culling because too many replacement heifers were retained the previous year, bred heifers are sold and tracked recognizing their market value is higher than heifers that are culled for other reasons. All culling functionality for cows is based on individual animal status (age, pregnancy and Johne's disease) and occurs within the cow agent.

Bulls are also culled every year based on age (maximum 6 years) or if they either test positive or have clinical Johne's disease based on transitions and messages within the cow agent. Bulls are purchased as yearlings based on an event in main as needed every year 1 month after calving and 2 months before first bull exposure to maintain a total number of bulls in the desired age range to meet the specified cow to bull ratio.

Events to capture model output data

Herd inventory numbers are captured by an event in a dataset June 30th and December 31st of each year. The prevalence of infected (but not infective) and infective cows greater than 1 year of age are saved each year at testing time before culling.

Other annual datasets created by an annual event December 31st of each year include calves born (male, female and total); calves died between birth and weaning; total number of calves weaned; male and female calves sold at weaning; retained heifer calves; pregnant replacement heifers purchased; mature cows purchased; purchased yearling bulls; replacement heifers sold or culled; mature females culled by age (> or <= 10) and BCS (>= or <2.5/5.0); cows sold due to old age > 12 years; mature cows sold because not pregnant; pregnant heifers sold for inventory adjustment; mature females sold due to positive MAP test results; mature females sold due to having clinical signs of Johne's disease; number tested by blood, individual fecal, or pooled fecal tests; average weight of weaned heifers and steers; and prevalence of infective animals after culling.

4 Design concepts

Basic principles

Johne's disease is challenging to study using experimental or observational methods due to the prolonged incubation period during which infected animals remain in a latent or subclinical state (17). Disease simulation models provide a potentially practical and cost-effective option for exploring questions related to Johne's disease. However, most previous models have been designed to address disease management in dairy herds. This agent-based model aims to address a gap in current research as few models have been developed for Johne's disease in beef cattle, none of which are specific to western Canadian beef herds.

Several studies have used ABMs to examine options for management of Johne's disease in dairy herds (21-28), but most have been reported within the last decade (21-25, 27-29). Earlier models were primarily compartmental and deterministic in design (16, 21-25, 27-29), with the exception of Kudahl, Østergaard (26). While some of these ABMs examine transmission pathways (21, 22, 27, 30), others have focused specifically on evaluating control measures (21, 23-28, 31). The direct costs associated with testing were considered by (27, 28), while some also report the associated impacts of MAP on herd productivity (24, 25, 27, 28). Two dairy ABMs examined more than one type of testing (27, 28). None of the models considered PCR tests, and only a few of the identified ABM reports compare the relative effectiveness of different testing frequencies for at least one the tests considered in the present configuration (23, 27). Other strategies for targeted testing based on individual animal parity, number of previous positive tests, days in milk and whether the dam was positive were also reported (28, 30). The current model in addition to being unique as depicting extensively managed cow-calf herds, provides a unique concurrent examination of a range of testing types, frequencies and risk-based testing options.

The present model reflects a typical western Canadian cow-calf herd with respect to herd size, structure and management practices. Previous dynamic simulation models for Johne's disease in beef herds reflect production practices that are common in European beef herds ((15, 32, 33). The need for region specific models for MAP control in dairy herds has been previously reported (23, 31).There are distinct differences in the structure and management of beef and dairy herds that must be accounted for in model design, as well as pathways for MAP transmission. There are also differences between the smaller, relatively more intensively managed beef herds found in most European countries and the extensively managed herds from North America targeted in this model (15, 16).

Beef cattle in western Canada and most areas of the north central and western United States are typically managed almost exclusively outdoors, grazed on extensive pastures or cover crops reducing the opportunity for focused areas of environmental contamination that can be readily modeled or targeted through management interventions. Calving occurs seasonally in late winter and spring in most areas of North America and typically outside on pasture or in paddocks. While the calving area is a focus of concern for control efforts (19, 34), the opportunities to precisely model a single meaningful environmental reservoir are limited. In

contrast, most dairy herds are managed indoors for at least some part of the year, most dairies in Europe and North America calve year-round, and many use individual animal calving pens creating differing opportunities for environmental disease transmission. One recent model of MAP transmission in Irish dairy herds did consider the impact of compact spring calving (31) highlighted the differences from year-round calving and the need for testing and culling prior to calving to reduce exposure to highly infectious cows present during the calving season. Previous research in beef cattle has identified Johne's disease suspect animals in the calving area as a strong risk factor for herd status (35).

The challenges of obtaining data for modeling MAP in beef herds was described in the first beef model identified (15). Data from recent regional observational studies in cow-calf herds were used in the current cow-calf model to inform many of the input parameters. For example, the initial within-herd prevalence as well as the initial prevalence in purchased animals incorporated in the model were based on a 2019 cross-sectional study aimed at estimating the prevalence of Johne's disease in Canadian cow-calf herds (5). The authors of the Johnson, McLeod (5) study used the Bayesian latent class model technique to estimate the sensitivity and specificity of ELISA, individual fecal PCR and pooled fecal PCR in both eastern and western Canadian cow-calf herds. The resulting diagnostic test performance estimates in western Canadian cow-calf herds were used as model input parameters in the current study. Although previous studies used other data from the literature to inform model input parameters, the data was commonly older, derived from parameter estimates reported by previous models, or not specific to the population of interest or the tests and laboratories being considered by the model (15, 23, 27, 30-32, 36). The present model integrates current and emerging research and surveillance data to inform Johne's disease management and control decisions on commercial cow-calf operations for western Canada; however, the model can be adapted for other regions by modifying parameter values.

While many of the previous models used similar types of epidemiological input parameters to the current model, there were some key differences. The current model captured vertical transmission from dam to calf *in utero* as well as via fecal-oral transmission either directly from the dam or indirectly from other infectious cattle through the environment. Most of the differences focus on the pathways for MAP exposure to the calf from fecal contamination. The probability of *in utero* transmission was based on stage of infection and a range of values from peer-reviewed publications based on dairy cattle (11). Many previously reported models considered the potential for *in utero* transmission as a fixed probability based on the stage of infection (15, 21, 24-26, 28) and with some referencing the meta-analysis by Whittington and Windsor (11) as either the sole source of the parameters (24, 25) or an influential source (23, 31).

The probability of transmission from the calf to the dam for the current model was derived from a calibration optimization exercise as existing data were based on dairy herds and calves where typically only remain with their dam for hours or days. Beef cows remain with their calves until weaning at 6-7 months providing more opportunities for dam to calf transmission.

This value, referred to in a recent review paper (13, 29) as pseudovertical transmission, was not included in the previous two beef models (15, 32). Dairy ABMs that included this risk typically focused on transmission at the time of birth and transmission through milk or colostrum (23-28, 30, 31).

Indirect transmission of infection was captured as a risk from the global environment depending on the prevalence of infectious animals and a contact parameter. The frequency of infective contact parameter reflecting the risk of MAP infection for preweaning calves given contact with MAP from infectious cattle was also based on a calibration exercise to estimate a value that would best reflect the longitudinal prevalence data from infected herds in western Canada (37). This estimate of the transmission risk associated with contact with other cattle and the environment is the most context specific of all model parameters and was not one where current literature could provide appropriate estimates. This value was then adjusted by previously reported estimates of the differential susceptibility of postweaning calves and adults (13) to reflect the rate of horizontal transmission in these age groups given contact with MAP from infectious cattle. The susceptibility adjustment for adults was based on an inverse function of age with the reported distribution apply to cattle at 1 year of age and decreasing to less than 1% in the oldest cows.

The value of the contact parameter will be highly dependent on the intensity of the management system and resulting opportunities for contact among animals and with contaminated environ2ments. Due to the number of management groups, pastures and pens used by a typical beef herd in western Canada throughout the production cycle and the varying density of animals within these areas and associated environmental conditions, there was no attempt to specifically model risk from a single or series of specific environmental reservoirs as has been reported in many of the dairy ABMs (23-25, 31).

The original source of the most analogous transmission parameters in the cited dairy models varies greatly. The transmission parameters range from assumptions, to values extracted from other observational studies in dairies, to calibrations that are either not specifically described or were reported as manual exercises, to values calibrated in other models and, in one case, to optimization of selected parameters using a random-forest classifier (21, 23, 24, 27, 28). One compartmental model (38) included calibration of unknown transmission rate parameters for specific environments by comparing model prevalence over time to published field data corrected for test characteristics. The resulting transmission rates were later cited in a series of ABMs (22, 23, 31). Another leveraged a unique dataset from 102 random farms with no control actions against MAP, generated a 3D parameter space and then visually identified the set of parameters that most closely resulted in a stable prevalence (24).

Although a calibration-based parameter estimation technique does suffer from limitations, we believe that it was a technique well suited for estimating the infective contact parameter and associated probability of infection from dam to calf before weaning for this model. The automated procedure used in this instance offers substantial advantages over manual calibration exercises in its optimization algorithms and capacity to manage stochastics and

uncertainty in other model inputs. For each proposed parameter value in the current model, 25 repetitions were completed for 4000 iterations to capture the impact of model stochastics and distributions of other parameters defined by the literature. While the authors (39-43) and others (23, 44-46) have previously contributed computational statistics and machine learning techniques that offer greater sophistication and can excel in supporting automated parameter estimation via sampling in higher dimensional parameter spaces, the current results were well supported by the optimization-based approach used here.

Most traditional calibration procedures matching to a single data source are best suited to estimating a single parameter. However, published longitudinal MAP data was very difficult to identify for infected beef herds. One unique longitudinal data source was developed in collaboration with industry and used in the actual calibration experiment for the present study. Further, the results of simulations for both the no testing and other limited ELISA-based testing scenarios were compared to two recent cross-sectional studies reporting within herd prevalence for cow-calf herds with evidence of MAP infection (5, 9). The range of apparent prevalence reported for positive herds in these studies ranged up to 15% to 45%.

While most of the previous models accounted for both vertical and horizontal direct transmission, fewer models describe age-specific environmental transmission as an infection pathway (15, 23-26, 28, 31). While calf to calf transmission has been documented in dairy herds (47), the relative importance of this pathway has been discounted in a previous modeling study. However, calf-to-calf transmission continues to be incorporated into some ABMs (23, 28, 31). In beef herds, where calves prior to weaning at six to seven months are typically grazed with large groups of cow-calf pairs, any potential impact of calf-to-calf transmission on MAP control efforts would be indistinguishable from the greater risk of transmission from either the dam or other infected cows or bulls in the management group. Calf-to-calf transmission was not explicitly modeled here as it would not have been feasible to estimate parameters for a third relatively minor contributor to the MAP transmission.

The period during which cattle are susceptible to infection is widely acknowledged as being an important source of uncertainty in modeling (16). Most researchers deemed animals to be resistant to MAP infection after 1 year of age (23, 26, 28, 31, 32, 36, 48) while some, including the present model and one of the beef papers, considered resistance to increase with age or acknowledged the potential for adult infections (27, 30, 49-51). The potential for adult transmission was retained in the current model based on the differences in productive lifespans for beef and dairy cattle. Cows greater than 10 years of age are relatively common in western Canadian cow-calf herds (7), while for dairy herds the productive lifespan has been reported to be less than 5 years (52). As such there is more opportunity for beef cows infected after 1 year of age to start to shed later in life regardless of whether they eventually develop clinical signs or not.

Model disease states were commonly split into the following categories: susceptible, latent, subclinical (moderate shedding), and clinical (high shedding), with the infection pressure stemming from the number of shedding animals in the herd, as in the present model, or

density of bacteria in the environment. Some models allowed for transient shedding in groups of calves (23, 31) and others also accounted for factors that have been shown to influence infection and disease onset, such as age at exposure (32, 48) or stressful events including calving or changes in feeding (26). In the current model, a distribution of transition times was used to inform progression of infection to shedding MAP and then apparent clinical disease. Other dairy ABMs reported latent periods consistent with the minimum values used in the present model, and that were consistent subclinical durations (23, 31). Shorter latent periods have previously been associated with dairy herds reporting higher herd prevalence (53). Other dairy models reported rates for these transitions that when converted to time to event were consistent with the values used in this model (21, 24, 25, 27, 30).

A variety of different diagnostic tests were used in previous models to identify infected animals. These tests included milk and serum ELISAs, and fecal culture used either separately or in combination. The identified ABMs included a single test sensitivity value for each stage of infection and an overall test specificity value based on previous research ((23, 28, 30-32, 36, 48, 49). In the present model, three diagnostic testing options were considered: serum ELISA, fecal PCR and pooled fecal PCR. Each test was described by a pert distribution for sensitivity and specificity for each stage of infection (infected, subclinical and clinical) and an overall distribution specificity value (5). This feature more completely reflects the uncertainty regarding the variation in test performance depending on the stage of infection than using a single value.

Fecal PCR has rarely been reported as a testing option in simulation studies (54). The peer-reviewed data describing the sensitivity of the commercial PCR test protocol are limited, but most of the existing reports of PCR sensitivity are lower than the value used here. Given that the original study from which the currently used value was derived was based on a relatively small number of PCR positive cows from infected herds (5), an independent sensitivity analysis was undertake to evaluate the impact of using the only other previously reported sensitivity values for the commercial PCR test used in the regional laboratory (VetAlert Johne's Real-Time PCR kit (Tetracore, Rockville, MD): 77.6% (95%CI 73.2-82.0) (55, 56) and 72% (**results section fits best with raw data*) (*60% reported in abstract*) (55, 56). However, when the values reported in the other papers (pert 77.6% (60%, 82%)) were used in place of the estimates generated for this population and laboratory, the individual PCR continued to perform better than pooled PCR in 75% of simulations for testing every 12 months.

The development of simulation models is based on available data from previous research. However, there are still many gaps in the literature related to the epidemiology and prevalence of Johne's disease, especially in beef cattle. Therefore, assumptions must be made for certain parameters where existing quantitative data is lacking. In the current model, assumptions were made about the minimum age at which animals can become infectious as well as the relative amount of infectious material shed from subclinical as compared to clinical cows in this environment. In examples of the most recently published ABMs some of the most common

disease associated parameters influenced by expert opinion included persistence of bacteria in the environment and impact of cleaning (23, 31), reduction of exposure due to calf rearing improvements, chance of getting infected from the environment (27), relative MAP shed in different disease states (26), and time in various disease states (24). In previous beef models, Humphry, Stott (15) assumed the bacterial survival rate in the winter months to be 10 times higher than the bacterial survival rate in the summer. Model building provides an opportunity to identify areas where current knowledge is lacking and allows these gaps to be highlighted for future research endeavors.

The objective of the present model was to identify strategies that were both effective and had low direct costs of testing for reducing the within-herd prevalence of Johne's disease in beef herds. The model described by Bennett, McClement (32) was designed to determine the effects of testing and culling and improved management practices on Johne's disease prevalence in UK beef herds, as well as the associated costs of implementing control measures. Bennett, McClement (57) examined the impact of improving the sensitivity of the ELISA test studied, but did not explore any other strategies. A dairy ABM examining the role of testing and culling with serum ELISA found that increasing testing frequency from every 2 years to every year was associated with increasing control success (23), and that this strategy was particularly effective in high prevalence herds. Only one compartment model was identified that examined twice yearly testing with serum ELISA. Approximately 75% of simulation runs at the 23rd year had a prevalence of zero when frequency of testing was increased from annual to semi-annual and the probability of fadeout within 25 years increased (36). Another model using serum ELISA and fecal culture in series, examined the impact of testing and culling based on parity (28), but did not find an overall economic benefit. Other ABMs examined the impact of milk ELISA which is not a realistic option for extensively managed beef herds (24, 26, 30). The current beef model is unique in the combination of test types, frequencies of testing and strategies for adopting risk-based testing or reducing testing numbers compared.

This model was built using an agent-based modelling approach to account for individual animal behaviors and interactions that occur within the broader herd environment. Previous studies in beef cattle (15, 32) involved the development and use of herd-level simulation models to determine the prevalence of Johne's disease, however, the resulting outputs did not demonstrate the changes in herd dynamics and infection status that occur over time. Herd structure and management practices along with diagnostic test performance specific to western Canadian beef herds are incorporated into the current model to provide a more accurate representation of MAP infection and control within the population of interest.

Emergence

The prevalence of MAP is the key result that emerges from the model and is a combination of testing choices, testing performance, and herd replacement decisions. As a result of changes in infection prevalence, testing choices and performance, the proportion of cows culled due to Johne's disease will also vary over time. These values also vary based on initial prevalence of

infection and the variability in time to progress through different stages of the infection, differing susceptibility based on age, varying risks of in utero transmission and stochastics associated with transmission rates.

Infection prevalence and the resulting number of clinical cases impacts body condition score, which in turn impacts the probability of a cow becoming pregnant and herd pregnancy rates.

In contrast, the total numbers of cows and bulls are governed by model mechanisms to ensure herd size either remains constant or increases as specified at a particular time. However, pregnancy success and weaning weights are to some extent emergent properties as the probability of pregnancy varies based on BCS which is impacted by Johne's clinical status. Similarly, calf weaning weights are lower for cows that are in the subclinical and clinical stage of infection, making weaning weight an emergent outcome. As well higher culling rates can lead to a larger proportion of bred replacement heifers which can lead to lower pregnancy rates, higher calf mortality and lower weaning weights.

In most scenarios, testing numbers are governed by model mechanisms. However, with risk-based testing where testing numbers are based on the number of animals where the dam is positive, the cow is purchased, or there is a fixed number of previous negative test results are emergent in response to the prevalence of infection. Similarly, the number of fecal samples that must be individually tested following pooled testing is an emergent result in response to the prevalence of infection and resulting number of positive pools.

Adaptation

There is very limited adaptation in this model. In almost all cases, cow agents simply follow rules that reproduce observed behaviors. These rules govern which animals become pregnant, which calves survive until weaning, which calves are infected by the dam with Johne's disease, and which animals are correctly classified by testing based on the sensitivity and specificity of the test.

Objectives

The primary objective measure is prevalence which is typically calculated as the proportion of animals that are infective (or potentially detectable by diagnostic tests). These include the animals that are in the subclinical and clinical stages of infection. The denominator is the total number of females greater than 1 year of age. The prevalence of animals that are infected but not yet infectious is also monitored.

Learning

Learning is not implemented in the model.

Prediction

Prediction is not implemented in the model.

Sensing

Sensing is not implemented in the model.

Interaction

Interaction occurs primarily through disease transmission. Calves born to an infective cow have a probability of being infected from *in utero* transmission based on a random draw for each simulation from a pert distribution. The probability of infection after birth is a reflection of the dam's status but is also impacted by the prevalence of infective animals in the herd. The prevalence of infection in the herd directly influences the rate of infection for all animals in the herd with the risk varying based on animal age and the extent of shedding dependent on the stage of infection within infective animals.

Risk-based testing and culling of daughters of positive dams depends on a memory of calves born to each cow and the capacity to use this information in decision making.

Stochasticity

There are several examples of random assignment and stochastics in this model. Inherent stochastics in ABMs and distributions included for parameter values in the model reflect the roles of chance, inherent biological variation and uncertainty due to limitations in the existing research that could impact predictions of MAP transmission, disease progression in individual animals and diagnostic test performance. For example, the rate of MAP transmission from direct fecal transmission or the environment was characterized as exponentially distributed for individual events within a simulation. The probability of *in utero* infection was drawn from a pert distribution as was the relative susceptibilities of calves after weaning and animals more than one year of age. Disease progression between progressive stages of infection and disease was based on a series of time delays randomly drawn from pert distributions and then implemented as stochastic processes for each animal where the selected value acted as the mean of an exponential distribution governing the time to transition in each animal. Diagnostic test sensitivity and specificity values were also randomly drawn from a pert distribution with each unique model run. Furthermore, the assignment of animal age and infection status at model initialization was random, as were calf sex and calf survival to weaning. Pregnancy status was an emergent combination of a random probability and the impact of Johne's disease on body condition score.

One of the beef models and most other previous dairy ABMs examining control options reported elements of randomness and stochasticity as well. The primary stochastic parameter in the compartmental model presented by Humphry, Stott (15) included vertical transmission from dam to calf *in utero*. The model by Robins, Bogen (27) described age and disease at initialization, successful calving, mortality, MAP transmission and progression and diagnostic test performance as stochastic processes. Other models specifically discussing sources of stochasticity note random initial starting conditions and stochastics associated with transmission rates (23, 30, 31). The present model is somewhat unique in that no other models were identified that incorporated uncertain parameter values as distributions in all

simulations. Rather most addressed uncertainty in important values through targeted local and global sensitivity analyses.

Collectives

The one example of a collective in this model is the listing of calves born to a particular cow.

Observation

The animation of animal movement on main allows confirmation of animal movements, timing of important events in the reproductive cycle including bull exposure to the cows, calving, weaning, retaining replacement heifers, purchase of replacements and calving.

Graphical output of the databases includes results confirming correct implementation of herd size stability and management options, culling and replacement options and reasons for culling, infection statistics including numbers of animals at each stage of infection for cows and bulls, source of infection, test numbers and performance, and prevalence of total infected and prevalence of infective animals. This output can be viewed as the model is running to validate the code is working as intended and to observe emergent outcomes.

5 Initialization

When the model is initialized on April 1st the pregnant cows and heifers calve. At initialization, the model simulates a herd size based on the specified number of cows and replacement yearling heifers with a bull cow ratio of 1 bull per 20 cows. The model time unit is 1 month, and the length of the time horizon is 10 years. The model parameters can be specified, through user interface at a simulation setup page.

The age structure of the cow herd is established using a customized database distributing the female herd between 14.3% yearling replacement heifers (in our example 50 replacement heifers/ (300 mature cows plus 50 replacement heifers) and then equally among ages from 2 years to 12 years. This has the effect of having a relatively high proportion of culls due to age early in the model but starts to level out over time. The BCS of cows at model initialization is then assigned based on custom distributions specific to cow age.

Cow and bull infection status at model start is based on initial prevalence of infected, subclinical and clinical animals and based on animals being of sufficient age to meet minimal time from infection to subclinical for subclinical infections, and minimum times to clinical infection for animals showing clinical signs. Data to support initial herd prevalence is based on observational studies from Canadian beef herds (9). Distributions of minimum times to specific stages of infection are supported by the available literature (17-20, 58).

6 Input Data

Weight and BCS distribution for different ages were employed to initialize cows' weight and BCS for both cows present at model initiation and for purchased cattle. The distribution of birth weights for calves and weaning weights for male calves from mature healthy cows were available in data as a distribution, which was then supplemented in other areas of the model with expected differences for dams of different ages, female calves and calves from cows with subclinical and clinical Johne's disease. The datasets are placed in Data agents, which are shareable to all components of the model. Data also contains custom datasets to inform the age and BCS distribution for purchased cows assuming these will be slightly younger and in better BCS than the animals being replaced in the herd.

Model Parameters

Name	Value	Reference
Initialize herd and infection status		
# Females at least 2 years old at start up	300	Expert opinion
# Yearling heifers retained for breeding at start up	50	Expert opinion
% Latent Infected (but not infectious) at Start (Latent Stage)	5.0%	Johnson, McLeod (5)
% Subclinical at Start (Subclinical Stage)	5.0%	Johnson, McLeod (5)
% Clinical at Start (Clinical Stage)	1.0%	Expert opinion
Cow to Bull Ratio (number of cows for every bull) (expert opinion for upper end of bulls on most farms)	20	Waldner, Kennedy (59) and expert opinion
% Heifer Calves Eligible as Replacements	50%	Expert opinion
Percent of cows pregnant in body condition 3.0-4.0/5.0	93.2%	Waldner, Parker (3)<br><br>Waldner, Parker (4)
Pregnancy rates for cows with BCS 2.5/5.0 and 2.0/5.0	90%, 78%	Calculated in the model from odds ratios reported in Waldner and Garcia Guerra (6)
Percent of heifers pregnant in body condition 3.0-4.0/5.0	90.3%	Waldner, Parker (3)<br><br>Waldner, Parker (4)

Name	Value	Reference
Pregnancy rates for heifers with BCS 2.5/5.0 and 2.0/5.0	86%, 71%	Calculated in the model from odds ratios reported in Waldner and Garcia Guerra (6)
% Calves from Cows Died Birth to Weaning – Cows	5.4%	Waldner, Parker (3)<br>Waldner, Parker (4)
% Calves from Heifers Died Birth to Weaning – Heifers	7.8%	Waldner, Parker (3)<br>Waldner, Parker (4)
Parameters impacting weaning weights		
Avg 205 Day Weaning Weight for Male Calves from Cows (lbs) 5 to 10 years	600 lbs (SD 20 lbs)	Expert opinion
Adjustment to expected 205 day weaning weight based on cow age	Calculating 205-day Self Adjusted Weaning Weights	BCRC (60)
- Female vs Male Calves: Weaning Weight Difference (lbs)	50 lbs	Expert opinion
- Dam has subclinical Johne's then calves have lower weaning weight (lbs)	90 lbs	Bhattarai, Fosgate (61)
- Dam has clinical Johne's then calves have lower weaning weight (lbs)	129 lbs	Bhattarai, Fosgate (61)
Parameters impacting cow BCS and weight		
Target weight for mature cows	1400 lbs (SD 60 lbs)	Expert opinion
Target weight for heifers at first bull exposure	65% of mature body weight	
Target weight for heifers at first calving	85% mature body weight	
Weight Change For Every BCS Change Of Cow	200 lbs	BCRC (62)

Name	Value	Reference
Planned herd size increases		
Option to increase herd size at some point during the experiment	False	
# of Years After Start to Increase Herd Size	0	
New target number of cows to calve after herd size increase	N/A	
Information on purchased cattle		
% Latent Infected (but not infectious) Purchased Bulls	1.0 %	Johnson, McLeod (5)
% Subclinical Infection Purchased Bulls	1.0 %	Johnson, McLeod (5)
% Latent Infected (but not infectious) Purchased Cows	1.0%	Johnson, McLeod (5)
% Subclinical Infection Purchased Cows	1.0%	Johnson, McLeod (5)
% Pregnant Heifers Purchased as Compared to % Purchased Pregnant Cows	0.0%	
Information on infection transmission		
Probability Subclinical Dam to Calf Transmission *In Utero*	0.09* (0.06,0.14)	Whittington and Windsor (11)
Probability Clinical Dam to Calf Transmission *In Utero*	0.39* (0.20,0.60)	Whittington and Windsor (11)
Coefficient representing shedding in subclinical cows as compared to clinical	0.2	Expert opinion
Probability of dan to calf transmission from birth to weaning from clinical cow	0.557**	Calibrated result<br><br>But similar to report by Windsor and Whittington (13)
Probability of dam to calf transmission from birth to weaning from subclinical cow	0.2 × 0.557**	

**Name**	**Value**	**Reference**
Frequency Of Infective Contact (effective contacts per year) = contact rate × probability of infection given infectious contact for preweaning calves (calves 0 to 7 mo)	46.89/year**	Calibration
Weighted prevalence of simulated MAP shedding intensity = (# subclinical cows × 0.2 + # clinical cows) / total cows > 2 years of age	--	
Infection rate / year = frequency of infective contact × weighted prevalence	--	
Minimum Age Infectious Months	18.0	Weber, Kogut (63)
Max Age Clinical Disease Years	10 years	Tiwari, VanLeeuwen (18)
Post Weaning Calf Intensity Modifier – reduced risk of infection after weaning age	0.50* (0.30,0.70)	Windsor and Whittington (13)
Adult Intensity of Infection Modifier – reduced risk of infection after 1 year of age	0.19* (0.10,0.32) × (1/current age in years)	
Infected Duration (Latent infection – not shedding)	Pert distribution (18.0, 60.0, 36.0) months	Elliott, Hough (58) Weber, Kogut (63)
Subclinical Duration (Low to moderate shedding) (With duration of latent infection leads to most animals developing clinical signs at 5 years with a range of 2.5 to 10 years.)	Pert distribution (6.0, 60.0, 24.0) months	Tiwari, VanLeeuwen (18)
Clinical Removal Duration (High shedding) (Time from development of clinical signs to removal – expert opinion for beef herds.)	Pert distribution (1.0, 4.0, 2.0) months	Expert opinion

Name	Value	Reference
Johne's management options		
Cull Daughters Of Positive Cows (includes cows that are clinical and cows that test positive if cows are tested)	False	
Management Impact Multiplier for Transmission Rate (Default set to 1, > 1 to increase risk, 0.1 to 1 to decrease transmission)	1.0	
Testing options		
Run Individual Fecal Tests	True/False	
Run Blood Tests	True/False	
Run Individual Fecal Tests	True/False	
Months after model start to test (April 1st)	7.0 months (Apr 1st + 210 days = Oct 28th)	
Test X Years After First Year (Initial Testing)	10.0	
Test X Years after Initial Testing (set to 0 if no reduced testing)	0.0	
# of Years Between Tests for Initial Testing Option	1.0	
# of Years Between Tests for Reduced Testing Option (set to 0 if no 2nd round of reduced testing)	0.0	
Factors limiting which cows are tested		
% of Eligible Cows to be Tested	100%	
Minimum Age to Test (years)	2.0	
Maximum Age to Test (years)	10.0	
Max BCS to test (1 very thin - 5 fat)	5	
Blood test characteristics		
Blood Test Infected Sensitivity Mode (Latent stage)	0	Whitlock, Wells (64)
Blood Test Infected Sensitivity Min	0	Whitlock, Wells (64)

Name	Value	Reference
Blood Test Infected Sensitivity Max	0	Whitlock, Wells (64)
Blood Test Subclinical Sensitivity Mode (Subclinical stage)	0.36	Johnson, McLeod (5)
Blood Test Subclinical Sensitivity Min	0.22	Johnson, McLeod (5)
Blood Test Subclinical Sensitivity Max	0.52	Johnson, McLeod (5)
Blood Test Clinical Sensitivity Mode (Clinical stage)	0.825	Bech-Nielsen, Jorgensen (65)
Blood Test Clinical Sensitivity Min	0.8	Billman-Jacobe, Carrigan (66)
Blood Test Clinical Sensitivity Max	0.87	Sweeney, Whitlock (67)
Blood Test Specificity Mode (All stages)	0.99	Johnson, McLeod (5)
Blood Test Specificity Min	0.98	Johnson, McLeod (5)
Blood Test Specificity Max	0.99	Johnson, McLeod (5)
Individual fecal test characteristics		
Fecal Test Infected Sensitivity Mode (Latent stage)	0	Whitlock, Wells (64)
Fecal Test Infected Sensitivity Min	0	Whitlock, Wells (64)
Fecal Test Infected Sensitivity Max	0	Whitlock, Wells (64)
Fecal Test Subclinical Sensitivity Mode (Subclinical stage)	0.96	Johnson, McLeod (5)
Fecal Test Subclinical Sensitivity Min	0.8	Johnson, McLeod (5)
Fecal Test Subclinical Sensitivity Max	1.0	Johnson, McLeod (5)
Fecal Test Clinical Sensitivity Mode (Clinical stage)	0.96	Johnson, McLeod (5)

Name	Value	Reference
Fecal Test Clinical Sensitivity Min	0.8	Johnson, McLeod (5)
Fecal Test Clinical Sensitivity Max	1.0	Johnson, McLeod (5)
Fecal Test Specificity Mode (All stages)	0.98	Johnson, McLeod (5)
Fecal Test Specificity Min	0.96	Johnson, McLeod (5)
Fecal Test Specificity Max	1.0	Johnson, McLeod (5)
Pool-based fecal test characteristics		
poolSize (# of samples per pool)	5	
Pool Based Fecal Test Infected Sensitivity Mode (Latent stage)	0	Whitlock, Wells (64)
Pool Based Fecal Test Infected Sensitivity Min	0	Whitlock, Wells (64)
Pool Based Fecal Test Infected Sensitivity Max	0	Whitlock, Wells (64)
Pool Based Fecal Test Subclinical Sensitivity Mode (Subclinical stage)	0.54	Johnson, McLeod (5)
Pool Based Fecal Test Subclinical Sensitivity Min	0.36	Johnson, McLeod (5)
Pool Based Fecal Test Subclinical Sensitivity Max	0.72	Johnson, McLeod (5)
Pool Based Fecal Test Clinical Sensitivity Mode (Clinical stage)	0.54	Johnson, McLeod (5)
Pool Based Fecal Test Clinical Sensitivity Min	0.36	Johnson, McLeod (5)
Pool Based Fecal Test Clinical Sensitivity Max	0.72	Johnson, McLeod (5)
Pool Based Fecal Test Specificity Mode (All stages)	1.0	Johnson, McLeod (5)

**Name**	**Value**	**Reference**
Pool Based Fecal Test Specificity Min	0.99	Johnson, McLeod (5)
Pool Based Fecal Test Specificity Max	1.0	Johnson, McLeod (5)
Risk based testing options		
Test All Bulls	False	
Test All Purchased Females	False	
Test Daughters of Positive Dams (Test Positive or Clinical Cases)	False	
Stop testing if X Previous Negative Test Results	False	
# Negative Tests To Stop Testing (Unlikely cow is infected with at least X negative tests)	4	
Options for testing high risk cows		
userControlledTestingFrequencyFlag	False (set to True to activate high risk specific options)	
highRiskifLessMaxBCS	False	
highRiskifIsBull	False	
highRiskifPurchased	False	
highRiskifDamPositive	False	
highRiskifNotMoreThanXNegativeTestResults	False	
LimitEligibilityForAllHighRiskTestingBasedOnNegativeTestingHistory	False	
percentageOfHighRiskBeingTested	100%	
ageOfFirstTestForHighRiskYears	2.0	
firstTestUntilForHighRisk	10.0	
secondTestUntilForHighRisk	0.0	
firstHighRiskTestingFrequency	1.0	

Name	Value	Reference
secondHighRiskTestingFrequency	0.0	
Options for testing low risk cows		
userControlledTestingFrequencyFlag	False (set to True for low-risk specific options)	
percentageOfLowRiskBeingTested	0%	
ageOfFirstTestForLowRiskYears	2.0	
firstTestUntilForLowRisk	10.0	
secondTestUntilForLowRisk	0.0	
firstLowRiskTestingFrequency	1.0	
secondLowRiskTestingFrequency	0.0	
poolBasedFecalTestFrequency	1 years	

*To simplify parameter calibration scenarios only the mean value was used. The impact of the distributions was then examined using the distributions in the Monte Carlo simulations and reported with the sensitivity analysis.

** Values used in comparison of testing and culling strategies.

7 Submodels

Production stage and cycle control submodels

The production stage and cycle control submodels are operationalized by state charts within cow considering the following factors:

- Weight and BCS (Body Condition Score) impact on female pregnancy rate
- Offspring inheritance of ADG characteristics from their mothers
- Population is maintained at a constant level unless instructions are received to increase herd size. Herd size is impacted each year by:
  - Removing open cows and cows > 12 years
  - Removing cows that are not pregnant
  - Removing bulls > 6 years
  - Weaning
  - Retaining home raised heifer calves for replacements
  - Purchase bull
  - Purchase cows
  - Purchase heifers

Ignored factors

- Limited tracking of males
  - Male calves are removed from the model at weaning after recording the number of calves and their average weight.
  - Bulls are purchased as yearlings and sold at 6 years of age.
- Limited tracking of individual interactions
  - Infection risk is not based on individual animal contacts other than between cows and their calves.
  - All animals after accounting for age are considered to be equally at risk of infection as animals are managed within the same environment.
- Twins are ignored.
  - We assume female cows always deliver one offspring each year. Calf mortality between birth and weaning varies between cows and heifers.
- Variation in event time between individuals
    - Day-differences among individuals are ignored for most events; for example, all caves are born at the same time

Exogeneous factors

- Time between states
  - The timeout between states (bull exposure, pregnancy testing, calving, weaning) is pre-defined.
- The number of bulls is fixed relative to the number of females.
  - There is 1 bull for every 20 cows. Bulls will be purchased to restore this number relative to culls or purchases to increase calving herd size.

- The maximum age for bulls and cows
    - Cows can reach a maximum of 12 years of age. Bulls are removed at a maximum age of 6 years old and then are replaced.
- Purchase decision
    - Purchase time is set for cows. All heifers are purchased pregnant following pregnancy testing. The default setting is to purchase all cows pregnant following pregnancy testing.
    - Ratio of purchase heifers relative to purchased cows. Given a certain total number of females need to be purchased to maintain or increase herd size, the user can define the proportion that are pregnant heifers. The default is 0%.
    - Target herd size after purchase (maintain herd size or increase herd size)
- Introduction of bulls and weaning of male calves.
    - Bulls for the breeding are purchased after an old bull is removed before the start of the next breeding season. All male calves are removed after weaning.

Endogenous factors

- Population Over time
    - The herd size is dynamic and depends on numerous factors, such as birth, removal by old age, infertility, purchase of cow and heifer, Johne's disease diagnosis, etc.) but is regulated by retaining heifers, purchasing cows and pregnant heifers, and selling pregnant heifers if needed to maintain herd size.
    - The resulting age distribution of the herd is endogenous.
- Culling and replacement of cows and bulls
    - Non-pregnant cows – numbers vary depending on proportion of heifers and Johne's prevalence impacting herd BCS
        - Heifers and cows in good body condition have pregnancy success of 0.903 and 0.932, respectively.
        - The probability of pregnancy decreases substantially for BCS < 3.0 and <2.5/5.0.
    - Old age – the proportion of cows culled for old age will vary over time and reflects establishing the herd with equal numbers in each age group as a simplifying assumption. It will also vary depending on culling cows prematurely due to Johne's disease (test results or clinical disease).
    - Positive Johne's test results – including true positives and false positives
    - Culling due to clinical Johne's disease
    - Numbers of weaned calves vary based on the number of cows and heifers calving
    - Purchase heifers, cows, and bulls

Johne's disease infection and progression model

The Johne's disease state chart simulates progression and spread of the disease by considering the following factors:

- Infection rate between cows and calves through fecal transmission, and direct infection from mother to calf (can be fecal transmission, contaminated milk or colostrum, or vertical transmission).
- Time in each state.

This module also informs many other aspects of the model through impacts on culling, BCS, and weaning weights.

Exogeneous factors

- Timeout between states; however, these are defined by distributions introducing variability among simulations.
- Probability of in utero infection – also defined by a distribution
- Probability of transmission from dam to calf from birth to weaning – Calibrated from SSGA data.
- Chance of infection from by animal age (pre-weaning and post weaning and after 1 year) relative to a preweaning calf < 7 months of age:
    - 0.5 – postweaning calf 7 months to ≤ 1 year
    - 0.2/(current age in years) – animals > 1 year
- Frequency Of Infective Contact
    - Values calibrated from SSGA data.
- Factors influencing weighted prevalence.
    - Subclinical Prevalence Modifier. The model assumes cows in subclinical state are 0.20 as likely to transmit infection than cows in clinical state.

Endogenous factors

- Number of cows in each state.
- Infection rate from fecal transmission.
    - The rate depends on prevalence of cows in clinical and subclinical, as well as rate of effective contact for fecal transmission (direct and indirect) as previously described.

Diagnostic Testing Model

The diagnostic testing module simulates fecal testing (individual and pooled) and blood testing to detect Johne’s disease. Testing is complicated because of the delay between infection and the time when the animal starts to shed the organism and is potentially detectable by diagnostic tests and also by the uncertainty regarding the performance of the diagnostic tests.

Exogeneous factors

- Test date, testing frequency, duration of testing
- Delay to get test result
- Delay to make removal decision based on test results.
- Testing sensitivity and specificity for each stage of infection.

Endogenous factors

- Cows diagnosed as true positive, false positives, true negatives and false negatives.

Weight and BCS dynamics

The weight and BCS module simulate the continuous change of daily weight gain and BCS. The module is encapsulated as an agent inside the cow agent, and acts as a key component to drive various key endogenous factors. The module considers the following factors:

- Age
- Gender
- Johne's disease state.
- ADG of offspring informed by characteristics of their dams

Ignored factors

- Daily weight gain for bulls is simulated until weaning.
- Cows do not gain weight due to growth after second calf.

Exogeneous factors

- BCS unit of decrease
    - For cows in Johne's clinical state, the BCS is dropped by 0.5 per month.
- Weight change for every BCS change.
    - Weight Change For Every BCS Change Of Heifer is 150 lbs (expert opinion).
    - Weight Change For Every BCS Change Of Cow is 200 lbs (62).
- Target weights at various ages. All the target weights are drawn from distributions.
    - Weight of calves at birth (expert opinion).
    - Weight of calves at 205 days of age for male calves from mature cows (age 5 to 10 years) (expert opinion and local sale weight data published by auction marts).
    - Weight of calves at 205 days of age variation based on cow age (60).
    - Weight of calves at 205 days of age if calf is a heifer vs a steer/bull.
    - Weight of calves at 205 days of age if the mother has subclinical Johne's disease.
    - Weight of calves at 205 days of age if the mother has clinical Johne's disease.
    - Weight of heifers at first exposure to bull.
    - Weight of heifers having first calf.
    - Weight of cows having second calf.

Endogenous factors

- Weight at specific time
- BCS at specific time

References

1. Grimm V, Berger U, Bastiansen F, Eliassen S, Ginot V, Giske J, et al. A standard protocol for describing individual-based and agent-based models. Ecological Modelling. 2006;198(1):115-26.

2. Grimm V, Railsback S, Vincenot C, Berger U, Gallagher C, Deangelis D, et al. The ODD Protocol for Describing Agent-Based and Other Simulation Models: A Second Update to Improve Clarity, Replication, and Structural Realism. Journal of Artificial Societies and Social Simulation. 2020;23.

3. Waldner CL, Parker S, Campbell JR. Correction: Identifying performance benchmarks and determinants for reproductive performance and calf survival using a longitudinal field study of cow-calf herds in western Canada. PLOS ONE. 2019;14(11):e0225401.

4. Waldner CL, Parker S, Campbell JR. Identifying performance benchmarks and determinants for reproductive performance and calf survival using a longitudinal field study of cow-calf herds in western Canada. PLoS One. 2019;14(7):e0219901.

5. Johnson P, McLeod L, Campbell J, Rousseau M, Larson K, Waldner C. Estimating the sensitivity and specificity of serum ELISA and pooled and individual fecal PCR for detecting Mycobacterium avium subspecies paratuberculosis in Canadian cow-calf herds using Bayesian latent class models. Front Vet Sci. 2022;9:937141.

6. Waldner CL, Garcia Guerra A. Cow attributes, herd management, and reproductive history events associated with the risk of nonpregnancy in cow-calf herds in Western Canada. Theriogenology. 2013;79(7):1083-94.

7. Waldner CL, Kennedy RI, Rosengren L, Clark EG. A field study of culling and mortality in beef cows from western Canada. Can Vet J. 2009;50(5):491-9.

8. Elghafghuf A, Stryhn H, Waldner C. A cross-classified and multiple membership Cox model applied to calf mortality data. Prev Vet Med. 2014;115(1-2):29-38.

9. Johnson P, Marfleet T, Waldner C, Parker S, Campbell J. Seroprevalence of Mycobacterium avium spp. paratuberculosis in cow-calf herds located in the prairie provinces of Canada. . Can Vet J. 2022;(In press).

10. Begg DJ, Whittington RJ. Experimental animal infection models for Johne's disease, an infectious enteropathy caused by Mycobacterium avium subsp. paratuberculosis. Vet J. 2008;176(2):129-45.

11. Whittington RJ, Windsor PA. In utero infection of cattle with Mycobacterium avium subsp. paratuberculosis: a critical review and meta-analysis. Vet J. 2009;179(1):60-9.

12. Hill BB, West M, Brock KV. An estimated prevalence of Johne's disease in a subpopulation of Alabama beef cattle. J Vet Diagn Invest. 2003;15(1):21-5.

13. Windsor PA, Whittington RJ. Evidence for age susceptibility of cattle to Johne's disease. Vet J. 2010;184(1):37-44.

14. Sweeney RW. Transmission of paratuberculosis. Vet Clin North Am Food Anim Pract. 1996;12(2):305-12.

15. Humphry RW, Stott AW, Adams C, Gunn GJ. A model of the relationship between the epidemiology of Johne's disease and the environment in suckler-beef herds. Vet J. 2006;172(3):432-45.

16. Marcé C, Ezanno P, Weber MF, Seegers H, Pfeiffer DU, Fourichon C. Invited review: Modeling within-herd transmission of Mycobacterium avium subspecies paratuberculosis in dairy cattle: A review. Journal of Dairy Science. 2010;93(10):4455-70.

17. Whitlock RH, Buergelt C. Preclinical and clinical manifestations of paratuberculosis (including pathology). Vet Clin North Am Food Anim Pract. 1996;12(2):345-56.

18. Tiwari A, VanLeeuwen JA, McKenna SL, Keefe GP, Barkema HW. Johne's disease in Canada Part I: clinical symptoms, pathophysiology, diagnosis, and prevalence in dairy herds. Can Vet J. 2006;47(9):874-82.

19. Truyers I, Jennings A. Management and control of Johne's disease in beef suckler herds. In Practice. 2016;38(7):347-54.

20. Mitchell RM, Schukken Y, Koets A, Weber M, Bakker D, Stabel J, et al. Differences in intermittent and continuous fecal shedding patterns between natural and experimental Mycobacterium avium subspecies paratuberculosis infections in cattle. Vet Res. 2015;46(1):1-10.

21. Al-Mamun MA, Smith RL, Schukken YH, Grohn YT. Modeling of Mycobacterium avium subsp. paratuberculosis dynamics in a dairy herd: An individual based approach. J Theor Biol. 2016;408:105-17.

22. Biemans F, Tratalos J, Arnoux S, Ramsbottom G, More SJ, Ezanno P. Modelling transmission of Mycobacterium avium subspecies paratuberculosis between Irish dairy cattle herds. Vet Res. 2022;53(1):45.

23. Camanes G, Joly A, Fourichon C, Ben Romdhane R, Ezanno P. Control measures to prevent the increase of paratuberculosis prevalence in dairy cattle herds: an individual-based modelling approach. Veterinary Research. 2018;49(1):60.

24. Kirkeby C, Graesboll K, Nielsen SS, Christiansen LE, Toft N, Rattenborg E, et al. Simulating the Epidemiological and Economic Impact of Paratuberculosis Control Actions in Dairy Cattle. Front Vet Sci. 2016;3:90.

25. Kirkeby C, Graesboll K, Nielsen SS, Toft N, Halasa T. Epidemiological and economic consequences of purchasing livestock infected with Mycobacterium avium subsp. paratuberculosis. BMC Vet Res. 2017;13(1):202.

26. Kudahl AB, Østergaard S, Sørensen JT, Nielsen SS. A stochastic model simulating paratuberculosis in a dairy herd. Prev Vet Med. 2007;78(2):97-117.

27. Robins J, Bogen S, Francis A, Westhoek A, Kanarek A, Lenhart S, et al. Agent-based model for Johne's disease dynamics in a dairy herd. Vet Res. 2015;46(1):68.

28. Verteramo Chiu LJ, Tauer LW, Al-Mamun MA, Kaniyamattam K, Smith RL, Grohn YT. An agent-based model evaluation of economic control strategies for paratuberculosis in a dairy herd. Journal of Dairy Science. 2018;101(7):6443-54.

29. Brock J, Lange M, More SJ, Graham D, Thulke HH. Reviewing age-structured epidemiological models of cattle diseases tailored to support management decisions: Guidance for the future. Prev Vet Med. 2020;174:104814.

30. Al-Mamun MA, Smith RL, Schukken YH, Grohn YT. Use of an Individual-based Model to Control Transmission Pathways of Mycobacterium avium Subsp. paratuberculosis Infection in Cattle Herds. Sci Rep. 2017;7(1):11845.

31. Biemans F, Ben Romdhane R, Gontier P, Fourichon C, Ramsbottom G, More SJ, et al. Modelling transmission and control of Mycobacterium avium subspecies paratuberculosis within Irish dairy herds with compact spring calving. Prev Vet Med. 2021;186:105228.

32. Bennett R, McClement I, McFarlane I. An economic decision support tool for simulating paratuberculosis control strategies in a UK suckler beef herd. Prev Vet Med. 2010;93(4):286-93.

33. Groenendaal H, Nielen M, Hesselink JW. Development of the Dutch Johne's disease control program supported by a simulation model. Prev Vet Med. 2003;60(1):69-90.

34. Roussel AJ. Control of paratuberculosis in beef cattle. Vet Clin North Am Food Anim Pract. 2011;27(3):593-8, vi.

35. Smith DR, Schomer TJ, Hinkley S, Clowser S, Galeota JA, Weiss JC, et al., editors. An Estimate of the Proportion of Beef Cattle Herds with Mycobacterium avium subspecies paratuberculosis-Infected Cattle and Associated Risk Factors. American Association of Bovine Practitioners Proceedings of the Annual Conference; 2005.

36. Lu Z, Schukken YH, Smith RL, Grohn YT. Stochastic simulations of a multi-group compartmental model for Johne's disease on US dairy herds with test-based culling intervention. Journal of Theoretical Biology. 2010;264(4):1190-201.

37. SSGA. Johne’s disease surveillance program 2022 [cited 2022 July 25]. Available from: https://skstockgrowers.com/johnes-disease-surveillance-program/.

38. Marce C, Ezanno P, Seegers H, Pfeiffer DU, Fourichon C. Within-herd contact structure and transmission of Mycobacterium avium subspecies paratuberculosis in a persistently infected dairy cattle herd. Prev Vet Med. 2011;100(2):116-25.

39. Duan L, Osgood N, editors. GPU Accelerated PMCMC Algorithm with System Dynamics Modelling. International Conference on Social Computing, Behavioral-Cultural Modeling and Prediction and Behavior Representation in Modeling and Simulation; 2021 July 4: Springer, Cham.

40. Li X, Keeler B, Zahan R, Duan L, Safarishahrbijari A, Goertzen J, et al., editors. Illuminating the Hidden Elements and Future Evolution of Opioid Abuse Using Dynamic Modeling, Big Data and Particle Markov Chain Monte Carlo. 11th International Conference on Social, Cultural, and Behavioral Modeling (SBP-BRiMS); 2018 Jul1 11-13; Washington, DC.

41. Osgood N, Liu J. Combining Markov chain Monte Carlo approaches and dynamic modeling. In: Rahmandad H, editor. Analytical methods for dynamic modelers. Cambridge, MA: MIT Press; 2015. p. 125-70.

42. Osgood N, Liu J, Dueck S, editors. Combining MCMC and Compartmental Modeling to Enhance Understanding of Chlamydia Control in Saskatchewan. 42nd Annual Meeting of the Statistical Society of Canada; 2014 May 28; Toronto, ON.

43. Safarishahrbijari A, Teyhouee A, Waldner C, Liu J, Osgood ND. Predictive accuracy of particle filtering in dynamic models supporting outbreak projections. BMC Infect Dis. 2017;17(1):648.

44. Lamperti F, Roventini A, Sani A. Agent-based model calibration using machine learning surrogates. Journal of Economic Dynamics and Control. 2018;90:366-89.

45. Platt D. A comparison of economic agent-based model calibration methods. Journal of Economic Dynamics and Control. 2020;113:103859.

46. Smith NR, Trauer JM, Gambhir M, Richards JS, Maude RJ, Keith JM, et al. Agent-based models of malaria transmission: a systematic review. Malar J. 2018;17(1):299.

47. van Roermund HJ, Bakker D, Willemsen PT, de Jong MC. Horizontal transmission of Mycobacterium avium subsp. paratuberculosis in cattle in an experimental setting: calves can transmit the infection to other calves. Vet Microbiol. 2007;122(3-4):270-9.

48. Groenendaal H, Nielen M, Jalvingh AW, Horst SH, Galligan DT, Hesselink JW. A simulation of Johne's disease control. Prev Vet Med. 2002;54(3):225-45.

49. Smith RL, Schukken YH, Gröhn YT. A new compartmental model of Mycobacterium avium subsp. paratuberculosis infection dynamics in cattle. Prev Vet Med. 2015;122(3):298-305.

50. Schukken YH, Whitlock RH, Wolfgang D, Grohn Y, Beaver A, VanKessel J, et al. Longitudinal data collection of Mycobacterium avium subspecies Paratuberculosis infections in dairy herds: the value of precise field data. Vet Res. 2015;46:65.

51. Pradhan AK, Mitchell RM, Kramer AJ, Zurakowski MJ, Fyock TL, Whitlock RH, et al. Molecular epidemiology of Mycobacterium avium subsp. paratuberculosis in a longitudinal study of three dairy herds. J Clin Microbiol. 2011;49(3):893-901.

52. De Vries A, Marcondes MI. Review: Overview of factors affecting productive lifespan of dairy cows. Animal. 2020;14(S1):s155-s64.

53. Fecteau ME. Paratuberculosis in Cattle. Vet Clin North Am Food Anim Pract. 2018;34(1):209-22.

54. Ly A, Sergeant ESG, Plain KM, Marsh I, Dhand NK. Simulation modelling to estimate the herd-sensitivity of various pool sizes to test beef herds for Johne's disease in Australia. Prev Vet Med. 2021;189:105294.

55. Alinovi CA, Ward MP, Lin TL, Moore GE, Wu CC. Real-time PCR, compared to liquid and solid culture media and ELISA, for the detection of Mycobacterium avium ssp. paratuberculosis. Vet Microbiol. 2009;136(1-2):177-9.

56. Laurin EL, Chaffer M, McClure JT, McKenna SL, Keefe GP. The association of detection method, season, and lactation stage on identification of fecal shedding in Mycobacterium avium ssp. paratuberculosis infectious dairy cows. J Dairy Sci. 2015;98(1):211-20.

57. Bennett R, McClement I, McFarlane I. Modelling of Johne's disease control options in beef cattle: A decision support approach. Livestock Science. 2012;146(2):149-59.

58. Elliott GN, Hough RL, Avery LM, Maltin CA, Campbell CD. Environmental risk factors in the incidence of Johne's disease. Crit Rev Microbiol. 2015;41(4):488-507.

59. Waldner CL, Kennedy RI, Palmer CW. A description of the findings from bull breeding soundness evaluations and their association with pregnancy outcomes in a study of western Canadian beef herds. Theriogenology. 2010;74(5):871-83.

60. BCRC. Record Keeping and Benchmarking: Level 3: Beef Cattle Research Council; 2020 [cited 2022 June 26]. Available from: https://www.beefresearch.ca/resources/recordkeeping/level-three.cfm.

61. Bhattarai B, Fosgate GT, Osterstock JB, Fossler CP, Park SC, Roussel AJ. Comparison of calf weaning weight and associated economic variables between beef cows with and without serum antibodies against or isolation from feces of Mycobacterium avium subsp paratuberculosis. J Am Vet Med Assoc. 2013;243(11):1609-15.

62. BCRC. Body Condition: Beef Cattle Research Council; 2022 [cited 2022 July 3]. Available from: https://www.beefresearch.ca/research/body-condition-scoring.cfm.

63. Weber MF, Kogut J, de Bree J, van Schaik G, Nielen M. Age at which dairy cattle become Mycobacterium avium subsp. paratuberculosis faecal culture positive. Prev Vet Med. 2010;97(1):29-36.

64. Whitlock RH, Wells SJ, Sweeney RW, Van Tiem J. ELISA and fecal culture for paratuberculosis (Johne's disease): sensitivity and specificity of each method. Vet Microbiol. 2000;77(3-4):387-98.

65. Bech-Nielsen S, Jorgensen JB, Ahrens P, Feld NC. Diagnostic accuracy of a Mycobacterium phlei-absorbed serum enzyme-linked immunosorbent assay for diagnosis of bovine paratuberculosis in dairy cows. J Clin Microbiol. 1992;30(3):613-8.

66. Billman-Jacobe H, Carrigan M, Cockram F, Corner LA, Gill IJ, Hill JF, et al. A comparison of the interferon gamma assay with the absorbed ELISA for the diagnosis of Johne's disease in cattle. Aust Vet J. 1992;69(2):25-8.

67. Sweeney RW, Whitlock RH, Buckley CL, Spencer PA. Evaluation of a commercial enzyme-linked immunosorbent assay for the diagnosis of paratuberculosis in dairy cattle. J Vet Diagn Invest. 1995;7(4):488-93.
