# Supplementary material for: Investigating effective testing strategies for the control of Johne's disease in western Canadian cow-calf herds using an agent-based simulation model
Source: Front Vet Sci. 2022 Nov 25;9:1003143. doi: 10.3389/fvets.2022.1003143 (PMC9732103; doi:10.3389/fvets.2022.1003143)
Supplement: Supplementary file 2 [file Data_Sheet_2.pdf]

Investigating effective testing strategies for the control of Johne's disease in western Canadian cow-calf herds using an agent-based simulation model

Supplementary material

Paisley Johnson, Lianne McLeod, Yang Qin, Nathaniel Osgood, Leigh Rosengren, John Campbell, Kathy Larson, Cheryl Waldner

Table A. Example of model validation output.

Baseline simulation with no Johne's disease - 10 years (5000 iterations per year)		
Examples of Reported Herd Metrics	Median (95% prediction interval)	IQR
total cows > 2 years of age to calve	263 (248, 281)	(258, 269)
total heifers to calve at 24 months	37 (20, 53)	(32, 43)
calves born total	300 (295, 305)	(300, 301)
calves born male	150 (133, 167)	(144, 156)
calves born female	150 (133, 167)	(144, 156)
calves died birth to wean	17 (10, 25)	(14, 20)
calves dead % total birth to wean	5.7 (3.3, 8.3)	(4.7, 6.6)
total calves weaned	283 (274, 292)	(280, 286)
male calves weaned and sold	142 (125, 159)	(136, 147)
avg weight steers weaned(lbs)	593 (589, 596)	(591, 594)
total females weaned	142 (125, 159)	(136, 147)
avg weight heifers weaned(lbs)	547 (544, 550)	(546, 548)
female calves weaned and sold	90 (64, 125)	(77, 104)
retained heifer calves after weaning	52 (22, 74)	(39, 64)
total cows exposed to bulls	300 (295, 305)	(300, 301)
total yearling heifers exposed to bulls	52 (23, 73)	(41, 63)
total females exposed to bulls	352 (323, 374)	(342, 364)
total bulls for breeding	19 (17, 20)	(18, 19)
cow to bull ratio	19 (18, 20)	(19, 20)
pregnant heifers purchased	0 (0, 0)	(0, 0)
pregnant cows purchased	0 (0, 24)	(0, 9)
cows sold by old age	19 (10, 32)	(15, 23)
cows and bred heifers sold not pregnant	29 (18, 40)	(25, 32)
total pregnancy rate	92 (89, 95)	, (91, 93)
pregnant heifers sold	3 (0, 30)	(0, 14)
females sold Johne’s test positive	0 (0, 0)	(0, 0)
females sold Johne’s clinical	0 (0, 0)	(0, 0)
purchased bulls	3 (0, 12)	(0, 5)
bulls sold old age	3 (0, 10)	(2, 4)
bulls sold Johne’s test positive	0 (0, 0)	(0, 0)
bulls sold Johne’s clinical	0 (0, 0)	(0, 0)
number tested blood	0 (0, 0)	(0, 0)
number tested fecal	0 (0, 0)	(0, 0)
number pools tested fecal	0 (0, 0)	(0, 0)

Supplementary Figures

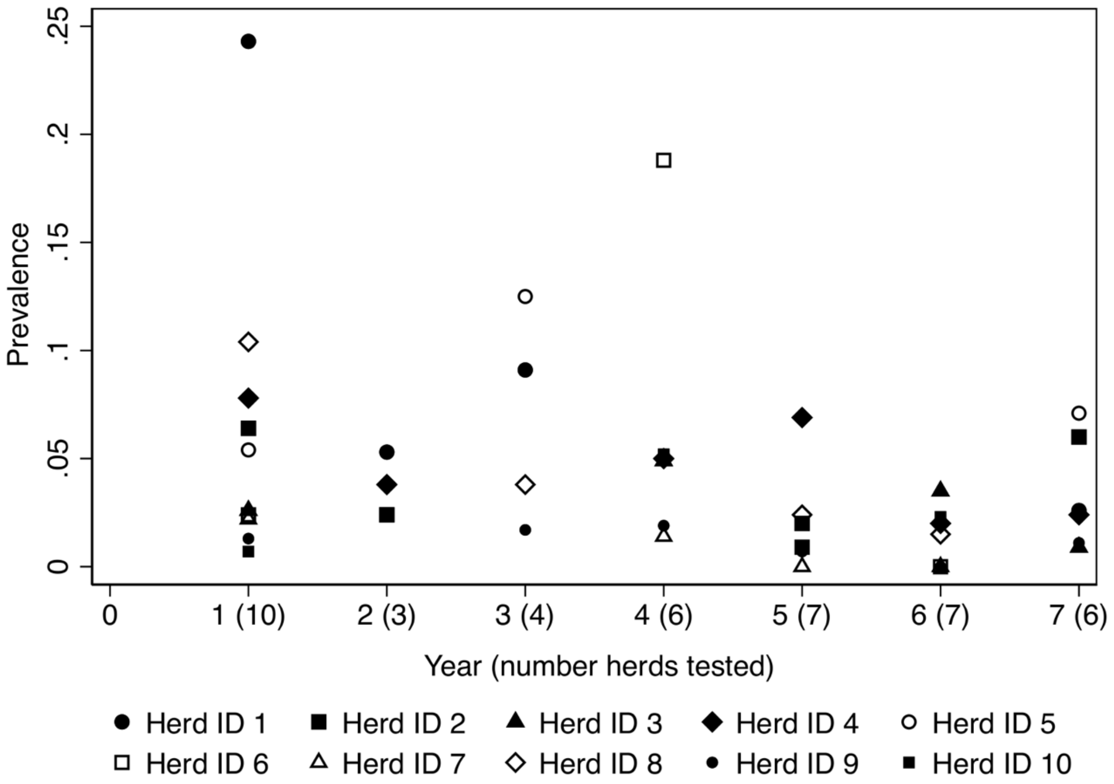

Figure A. Herd serum ELISA prevalence data from Johne's disease testing from 10 herds over 7 years used for model calibration (shared anonymously by the Saskatchewan Stock Growers Association; number of herds with data at each testing point in parentheses after each year).

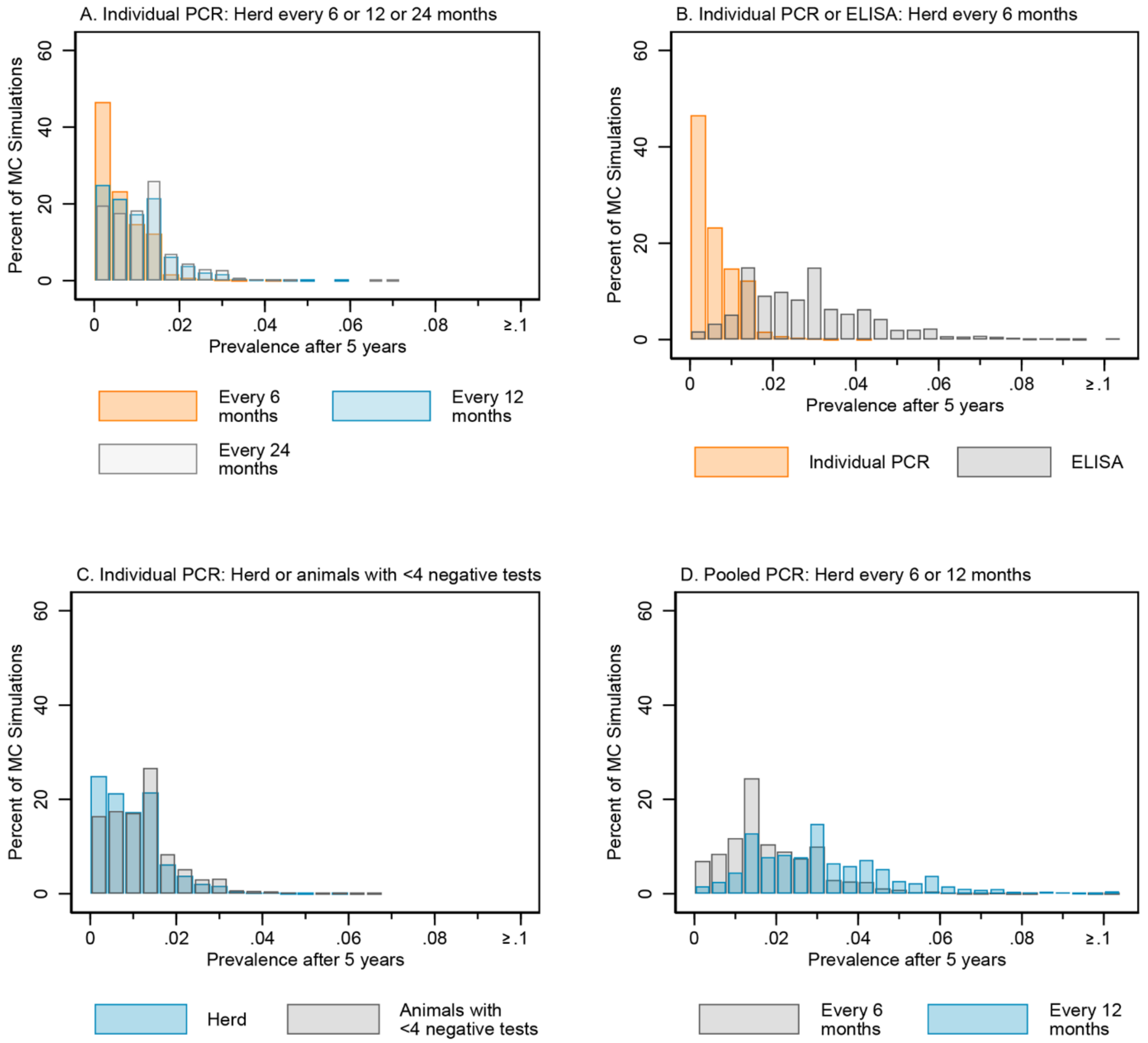

Figure B. Distribution of Monte Carlo simulated prevalence after 5 years for comparisons of seven key testing scenarios: individual fecal PCR testing every 6, 12, and 24 months (A), individual fecal PCR and serum ELISA every 6 months (B), individual fecal PCR for the whole herd and for animals with < 4 negative tests every 12 months (C), and pooled fecal PCR every 6 and 12 months (D).

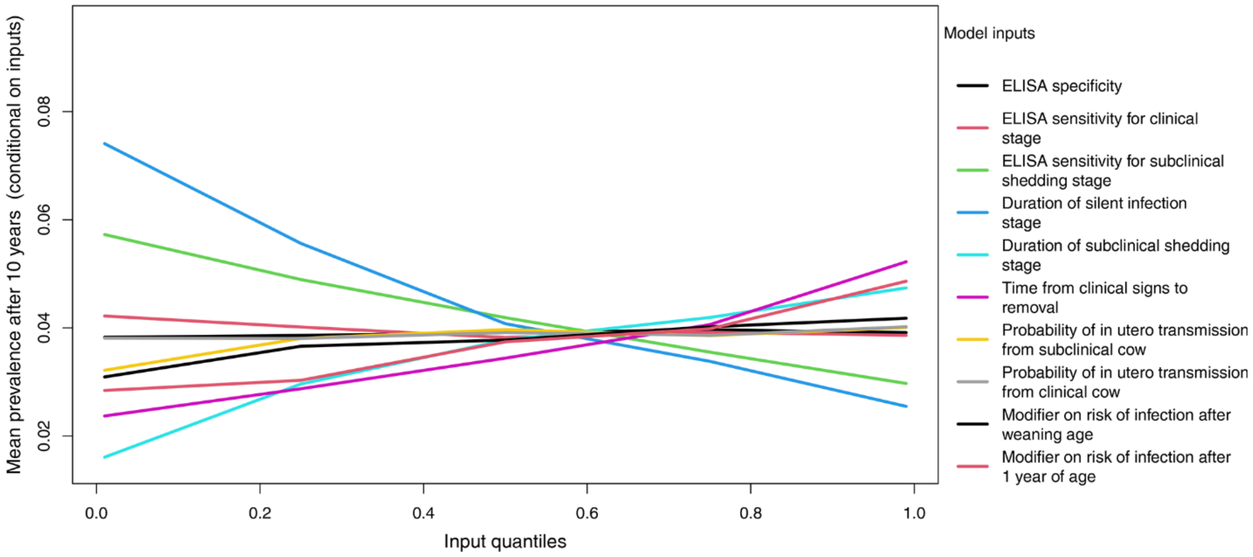

Figure C. Spider plot demonstrating the influence of all model inputs represented by distributions on variability of the mean MC simulated MAP prevalence after 10 years of whole herd testing with ELISA every 12 months. Inputs included the probability of *in utero* transmission to calves from clinical and subclinical cows, relative susceptibility of postweaning calves and adults, ELISA specificity, ELISA sensitivity for the subclinical shedding and clinical stages, the duration of the silent and subclinical shedding stages (months), and the time from detection of clinical signs to removal from the herd (months).

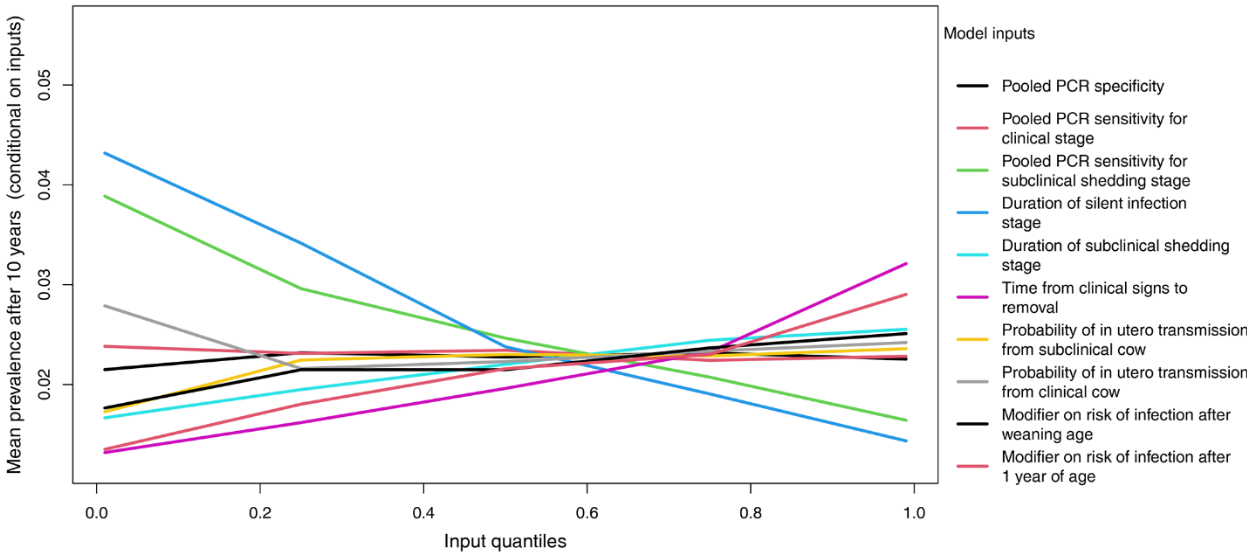

Figure D. Spider plot demonstrating the influence of all model inputs represented by distributions on the variability of the mean MC simulated MAP prevalence after 10 years of whole herd testing with pooled PCR every 12 months. Inputs included the probability of *in utero* transmission to calves from clinical and subclinical cows, relative susceptibility of postweaning calves and adults, pooled PCR specificity, pooled PCR sensitivity for the subclinical shedding and clinical stages, the duration of the silent and subclinical shedding stages (months), and the time from detection of clinical signs to removal from the herd (months).

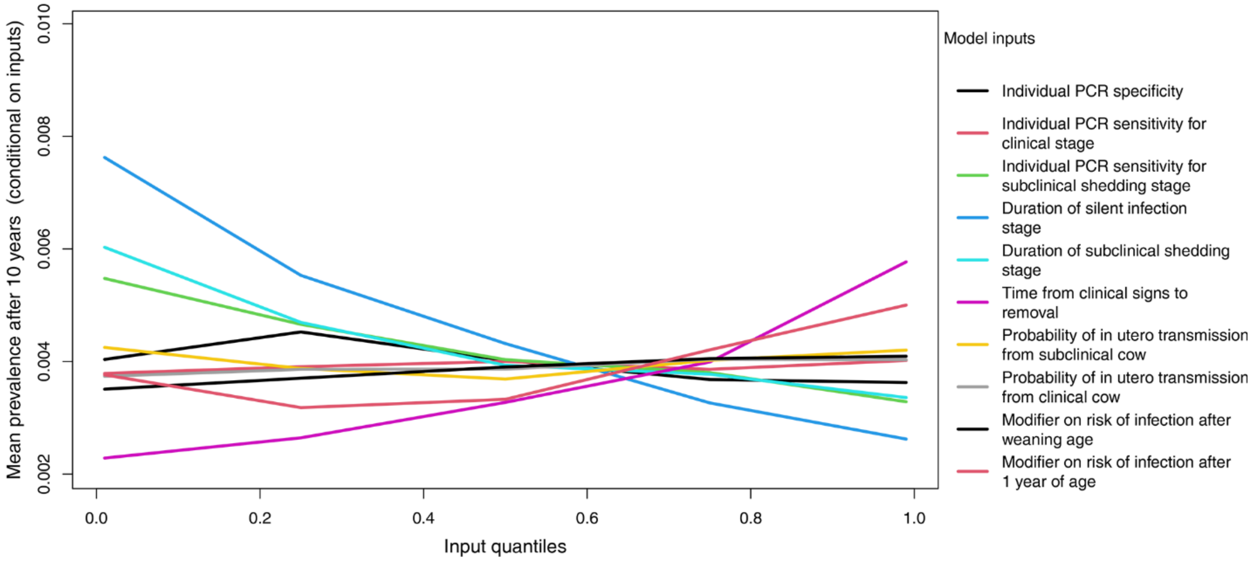

Figure E. Spider plot demonstrating the influence of all model inputs represented by distributions on the variability of the mean MC simulated MAP prevalence after 10 years of whole herd testing with individual PCR every 12 months. Inputs included the probability of *in utero* transmission to calves from clinical and subclinical cows, relative susceptibility of postweaning calves and adults, individual PCR specificity, individual PCR sensitivity for the subclinical shedding and clinical stages, the duration of the silent and subclinical shedding stages (months), and the time from detection of clinical signs to removal from the herd (months).
